# Molecular Switches at the Synapse Emerge from Receptor and Kinase Traffic

**DOI:** 10.1371/journal.pcbi.0010020

**Published:** 2005-07-29

**Authors:** Arnold Hayer, Upinder S Bhalla

**Affiliations:** 1 National Centre for Biological Sciences, Bangalore, India; 2 École Supérieure de Biotechnologie de Strasbourg, Strasbourg, France; University College London, United Kingdom

## Abstract

Changes in the synaptic connection strengths between neurons are believed to play a role in memory formation. An important mechanism for changing synaptic strength is through movement of neurotransmitter receptors and regulatory proteins to and from the synapse. Several activity-triggered biochemical events control these movements. Here we use computer models to explore how these putative memory-related changes can be stabilised long after the initial trigger, and beyond the lifetime of synaptic molecules. We base our models on published biochemical data and experiments on the activity-dependent movement of a glutamate receptor, AMPAR, and a calcium-dependent kinase, CaMKII. We find that both of these molecules participate in distinct bistable switches. These simulated switches are effective for long periods despite molecular turnover and biochemical fluctuations arising from the small numbers of molecules in the synapse. The AMPAR switch arises from a novel self-recruitment process where the presence of sufficient receptors biases the receptor movement cycle to insert still more receptors into the synapse. The CaMKII switch arises from autophosphorylation of the kinase. The switches may function in a tightly coupled manner, or relatively independently. The latter case leads to multiple stable states of the synapse. We propose that similar self-recruitment cycles may be important for maintaining levels of many molecules that undergo regulated movement, and that these may lead to combinatorial possible stable states of systems like the synapse.

## Synopsis

One of the key cellular changes that accompanies memory formation is a change in the efficacy of synaptic connections between nerve cells. Such changes may arise from long-lasting changes in the number of receptor ion channels at the synapse, and also from changes in their conductance. The authors ask how the cell maintains these changes despite molecular turnover, traffic, and biochemical noise. They use computer simulations as an “in silico” microscope to extrapolate biochemical and light microscopy measurements down to sub-synaptic volumes.

Based on these computer models, the authors propose that there is a self-sustaining switch involving the movement of receptors (AMPA receptors) to and from the synaptic membrane. The switch works because the presence of sufficient receptors at the membrane biases the trafficking machinery to recruit still more receptors. This switch has suggestive parallels with experimental observations of the conversion of synapses from silent to active, which involves AMPA receptor insertion. The authors show that yet more conductance states may arise through interactions with a biochemical switch involving a synaptic kinase (CaMKII).

This receptor switch illustrates how the cell may harness molecular turnover and traffic to maintain, rather than wash out, cellular structures and states.

## Introduction

Long-term storage of neuronal information is believed to occur through alterations in synaptic efficacy. Many mechanisms have been identified for changes in synaptic strength, including modulation of neurotransmitter release, conductivity changes in receptors, changes in numbers of receptors or active synapses, and structural alterations of the synapse. Among these, the insertion of glutamate receptors of the alpha-amino-3-hydroxy-5-methyl-4-isoxazole propionate (AMPA) subtype into the postsynaptic membrane and the modulation of receptor conductance by phosphorylation are key events in modulating synaptic efficacy. A fundamental issue challenges all of these mechanisms: how can they last a lifetime?

Synaptic memories can decay in at least three ways: turnover, diffusive exchange, and stochasticity. Turnover of major postsynaptic molecules ranges from periods of a few minutes to a few days and may be further enhanced by synaptic activity [1]. One solution to loss of memory due to molecular turnover is the concept of self-sustaining molecular switches [2]. These typically involve some form of molecular feedback giving rise to chemical systems, which can stably settle into one of two states. Such two-state, or bistable, systems can store information in a binary manner. Provided there is a steady supply of replacement molecules, molecular turnover can be tolerated, since newly synthesised, naïve molecules become entrained to the current state of the system. Some current proposals for such bistable synaptic switches include the calcium calmodulin type II kinase (CaMKII) hypothesis [3], a mitogen-activated protein kinase (MAPK) feedback loop [4,5], and, recently, the mammalian target of rapamycin (mTOR) protein synthesis loop [6]. Of these, the CaMKII model has been posed in the most detail with the most complete structural correlates. According to this model, CaMKII at the synapse can undergo autophosphorylation, which leads to activation of the kinase. The activated kinase molecules catalyze the phosphorylation of yet more CaMKII molecules, resulting in a self-sustaining cycle. The MAPK feedback loop model also involves a self-sustaining cycle, but in this case several intermediate molecules participate in the loop. The protein synthesis loop model is based on the observation of local protein translation machinery associated with synapses. Messenger RNA for several proteins, including the ribosomes themselves, is also present. Thus, high local protein synthesis creates the machinery for maintaining high levels of synthesis. This protein synthetic loop is regulated by mTOR.

The second mechanism for decay of synaptic memory is diffusive exchange of synaptic proteins, leading to washout of specific states in the synapse. Extrapolations from free diffusion constants suggest that diffusive exchange between the synaptic spine and dendrite is likely to be rapid, under 10 s even for proteins [7]. The postsynaptic density (PSD) is an elaborate cytoskeletal and signalling complex that provides anchors for synaptic proteins close to the region of presynaptic neurotransmitter release. This anchoring solves the problem of free diffusion and washout of active molecules, but introduces the problem of regulating the insertion of molecules into the correct locations. There is considerable evidence for targeted trafficking of molecules to and from the PSD. One such trafficking cycle is the insertion and removal of glutamate receptors of the AMPA subtype into the synaptic membrane [8]. A striking and physiologically important example of receptor insertion is the conversion of silent synapses, lacking AMPA receptors (AMPARs), into active synapses with a full complement of receptors (reviewed in [8]). The delivery of AMPARs to the synaptic membrane involves two streams: a constitutive pathway involving glutamate receptor heteromers 2 and 3 (GluR23), and an activity-dependent pathway involving GluR12 [9]. Based on current evidence, the activity-dependent insertion of GluR12 into the synaptic membrane is stimulated by phosphorylation on Ser845 [10,11]. There is also evidence for such phosphorylation being implicated in synaptic plasticity [12,13]. CaMKII also translocates to the PSD upon calmodulin (CaM) binding and stimulation [14]. Thus, in addition to their known involvement in synaptic plasticity, AMPARs and CaMKII have mechanisms for activity-dependent recruitment to the PSD in a manner that acts counter to washout processes [15]. This combination of attributes makes these molecules interesting candidates for analysing molecular memory mechanisms. Nevertheless, over the long term, even anchoring events are reversible and additional processes must be considered for stability.

A third important obstacle to stable memory formation is biochemical stochasticity. This causes uncertainty (noise) in the outcome of biochemical reactions involving small numbers of molecules. Such fluctuations are severe at the synapse, where many important signalling molecules are present in low numbers, that is, less than 100 molecules. In a typical synaptic volume of 0.1 fl [16] there are an estimated five free Ca^2+^ ions. Under stochastic conditions, there is a finite probability of spontaneous state flips in bistable molecular switches [7,17]. The lifetime of the stable states depends both on reaction rates and on the number of molecules. For example, the proposed MAPK switch does not fare well in synaptic volumes, and spontaneously flips state on the time scale of minutes [7]. Nevertheless, these time estimates are highly dependent on assumptions about diffusion, anchoring, and the levels of noise in other synaptic pathways.

Putting these themes together, a plausible synaptic memory mechanism might look like a bistable molecular switch that is resistant to turnover, incorporates traffic of molecules to and from the PSD, and is unlikely to spontaneously flip state even when small synaptic molecule numbers are taken into account. In this study we report a novel glutamate (AMPA) receptor–based switch that emerges from a consideration of its traffic and satisfies these criteria. We also examine a possible CaMKII switch in the context of these criteria. Finally, we integrate these switches to explore how multiple synaptic states may arise [18].

## Results

Our study proceeded in three stages: model construction and exploration, then examination of regulation and bistability, and finally consideration of interactions between the two forms of bistability.

### Model Construction

**Figure 1:**
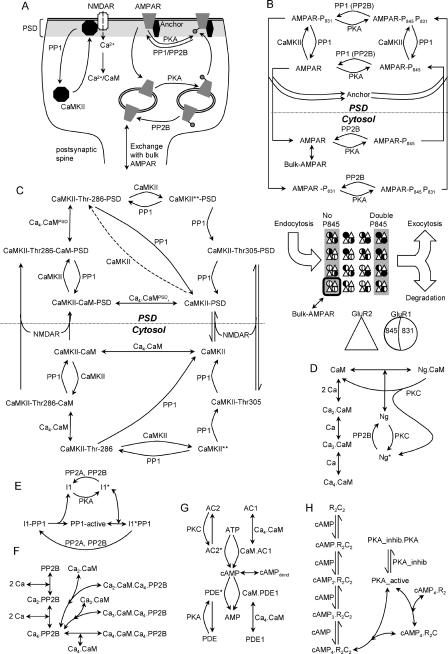
Model Structure (A) Overview of model, indicating key trafficking steps for AMPAR and CaMKII. (B–H) Chemical reaction schemes for pathways in model. Curved lines with arrows are enzymatic reactions catalyzed by molecules at the curves. Straight lines represent binding or unimolecular reactions. (B) Details of AMPAR model. The modelled AMPAR is a tetramer with two subunits each of GluR1 (circles) and GluR2 (triangles). There are 16 phosphorylation states each in the cytosol and synaptic membrane. These are represented in expanded form in the lower portion of (B), which shows the internalised pools of receptors. Black filling of the left half of the GluR1 circle indicates phosphorylation of Ser845, and of the right half indicates phosphorylation of Ser831. Endocytosis occurs for the receptors with no GluR1-Ser845 phosphorylation, and exocytosis and degradation occur for the receptors with both GluR1 subunits phosphorylated on the Ser845 site. Exchange of receptors with the bulk AMPAR pool occurs for the unphosphorylated state only, outlined in black. (C) CaMKII model. The dashed line for phosphorylation of CaMKII–PSD is applicable only for the bistable CaMKII models described in Figure 7. (D) CaM activation. (E) PP1 activation. (F) PP2B (calcineurin) activation. (G) cAMP formation. The unstimulated phosphodiesterase molecules (PDEs) also degrade cAMP, but at a lower rate than the activated forms illustrated. In the cAMP model we include diffusive exchange of cAMP with a dendritic compartment. (H) PKA activation. AMP, adenosine monophosphate; ATP, adenosine triphosphate; I1, inhibitor of PP1; Ng, neurogranin; PKA_inhib, inhibitor of PKA; PKC, protein kinase C; PP2A, protein phosphatase 2A.

The key molecules in the simulation were CaMKII, AMPAR, and their main regulators: protein kinase A (PKA), protein phosphatase 2B (PP2B, also known as calcineurin), and protein phosphatase 1 (PP1). As discussed in the methods, parameters for these pathways were derived from previously published models available in the DOQCS database [19] and were refined using known synaptic parameters. Resting Ca^2+^ concentrations were 80 nM in all models, and at these levels there is very little PP2B or CaM activation. We expanded the models to include translocation steps using simple binding steps with synaptic anchor proteins. These binding steps are assumed to include diffusive movement of molecules between the cytosol and PSD. As shown in Figure 1, activation of CaMKII or its Thr286-phosphorylated state through CaM binding causes it to move toward the PSD. Likewise, phosphorylation of AMPAR on Ser845 causes it to move from internal pools to the synaptic membrane.

Our simulations use six models.

#### Model 0.

**Figure 2:**
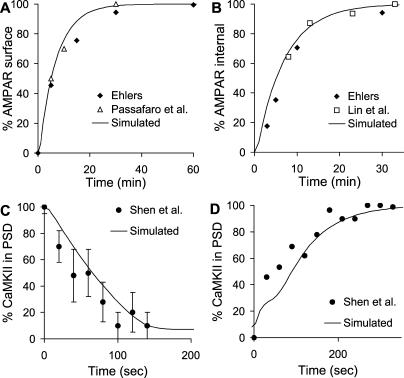
Matching Models to Trafficking Time Course (A) AMPAR exocytosis time course; experiments from [10,20]. (B) AMPAR endocytosis time course; experiments from [10,21]. (C) CaMKII internalisation time course; experiments from [14]. (D) CaMKII traffic to PSD; experiments from [14].

**Figure 3:**
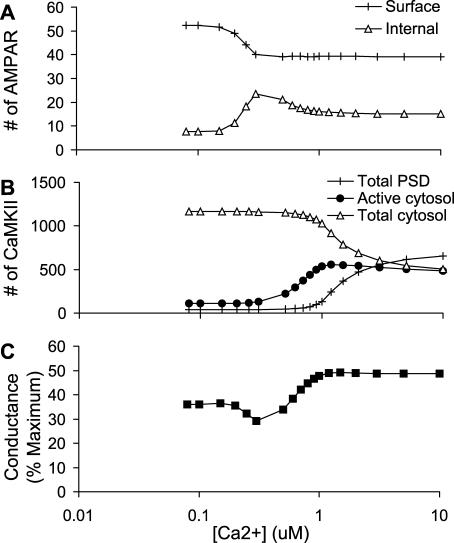
AMPAR and CaMKII Trafficking and Dependence on Steady Ca^2+^ Concentrations (A) Number of AMPARs in internal and synaptic membrane pools; AMPARs complexed to enzymes are not counted. (B) Number of CaMKII molecules in the cytosol and PSD. The activity in the cytosol and PSD starts to rise at about 0.5 μM Ca^2+^, but translocation occurs around 1 μM. (C) Conductance of membrane-inserted AMPARs. Receptor conductance is calculated by assuming that CaMKII phosphorylation of a single GluR1-Ser831 of the tetramer gives 1.5-fold basal conductance, and of two Ser831 gives 2-fold basal conductance. The conductance dips at around 300 nM Ca^2+^, when PP2B is active but CaMKII has yet to become fully active.

This model includes all the reactions in Figure 1, with three exceptions: (1) the CaMKII autophosphorylation step shown by the dashed line in Figure 1C, (2) the exchange of synaptic AMPAR with the bulk AMPAR in the dendrite in Figure 1B, and (3) the receptor degradation in Figure 1B. This model was used to fit the receptor trafficking curves, and to explore basic regulatory mechanisms.

#### Model 1: AMPAR bistability.

This model includes all reactions in Figure 1, with the exception of the CaMKII autophosphorylation step in the dashed line in Figure 1C. All parameters are identical to those in model 0. This is the reference bistable model.

#### Model 2: Skeletal version of AMPAR bistability.

This model is used to understand bistability mechanisms.

#### Model 3: CaMKII bistability.

This model includes reactions from Figure 1C–1F. It explicitly includes the CaMKII autophosphorylation step in Figure 1C. Rates for a few of the CaMKII reactions are somewhat modified compared to model 1.

#### Model 4: Combined AMPAR and CaMKII bistability.

This model combines models 1 and 3, using the model 3 parameters where there are differences. The concentration of bulk AMPAR is reduced slightly. This version uses the same PP1 enzyme for AMPAR and CaMKII in the PSD.

#### Model 5: Combined AMPAR and CaMKII bistability.

This model combines models 1 and 3, using model 3 parameters where there are differences. The concentration of bulk AMPAR is reduced slightly. This version uses distinct PP1 enzymes for AMPAR and CaMKII in the PSD.

Following the initial model construction based on existing models, we wished to parameterize receptor trafficking rates. We represented movement of AMPAR to the PSD as a single binding reaction with an anchor protein. The reverse movement was modelled as a similar reaction releasing AMPAR from the anchor. These simple reactions are approximations to several cellular events, presumably including binding to anchor and diffusive or active movements of the receptor–anchor complex. As we did not have direct biochemical rates for these steps, we fit the reaction rates to published observations of AMPAR movement from labelling and microscopy studies in live-cell preparations. We used model 0 for these calculations since we wished to model AMPAR movement only between the internal and surface-membrane-anchored states. Model 0 does not consider exchange of AMPAR with the bulk, or receptor degradation, and is therefore easier to match to the AMPAR recycling experiments.

To represent the experimental pulse-labelling of receptor, we needed to monitor the movement of a small amount of receptor (the pulse) without perturbing the activity levels of the regulatory molecules in the AMPAR trafficking cycle. Experimentally this is possible because the labelled receptors seamlessly participate in the same reactions as the unlabelled ones. In the simulations, however, a pulse of receptors would result in a displacement from steady state. We therefore simulated these experiments by first computing the steady-state levels of all molecules interacting with the receptor: CaMKII, PKA, PP1, and anchor proteins. We then numerically fixed the levels of each of these interacting molecules to their steady-state levels. Finally, we introduced the test pulse of receptors into the PSD or internal compartment and simulated its movement time course. Since the interacting molecules were held fixed at their steady-state levels, this procedure had the same effect as a pulse of labelled receptors that did not perturb the steady state.

We first matched rates for surface targeting of GluR12 subunits from experimental studies, shown in Figure 2A. The original experiments used fluorescent antibodies [20] and biotinylation [10] to monitor levels of surface and internalised receptors and hence to compute recycling rates. Despite these divergent techniques, the experimental curves for receptor exocytosis matched each other well, and the simple AMPAR movement model was a good fit to both curves.

In a similar manner, we matched rates for receptor internalisation from Ehlers [10] and Lin [21], shown in Figure 2B. Again, we were able to use a simple model of translocation to obtain a good fit to both sets of experiments.

A similar process was used for CaMKII to match the time course of activity-dependent PSD translocation and removal as monitored by light microscopy [14]. In the case of CaMKII, the role of the anchor protein was served by the N-methyl-D-aspartate receptor (NMDAR) (see Figure 1C). Again, a simple binding reaction was sufficient to fit the experimental data points (Figure 2C and 2D).

### Model Exploration

At this point we had a model (model 0) that fit biochemical and cell-biological observations on AMPAR and CaMKII trafficking. This model was descriptive in that it was effective in replicating the data that had been used to set it up in the first place. We then asked whether the model was sufficiently complete to match more complex system effects that had not been used to set parameters for the model. This is often a valuable way to assess whether a model is likely to be a reasonable representation of a complex system.

We first considered AMPAR behaviour at different concentrations of steady Ca^2+^ (Figure 3A). The resting level of Ca^2+^ was 80 nM. From the simulations, we found that there was a small decline in the number of AMPA receptors at the synapse when Ca^2+^ exceeded 300 nM. This was due to the activation of calcineurin, which dephosphorylates the receptor on the PKA site, and hastens its return to the cytosol.

We also computed a similar calcium-dependent curve for CaMKII (Figure 3B). Here the cytoplasmic activity of the kinase rose, followed by translocation to the PSD. The initial dip due to PP2B activity was not present in our model of CaMKII. This is because we had very little autophosphorylated CaMKII in the PSD in the basal state, so there was little substrate present for PP2B-activated PP1 to act upon.

AMPAR conductance is a function both of the number of receptors at the surface membrane, and of their phosphorylation state (see Materials and Methods). In Figure 3C we computed conductance. At low Ca^2+^, it closely tracked the number of membrane-inserted AMPARs. At near 1 μM Ca^2+^ the conductance of the receptor rose, because CaMKII was activated and phosphorylated the receptor on Ser831. The net effect of these competing events was that AMPAR conductance first declined below baseline, and then rose above baseline. This is consistent with the theoretical Bienenstock-Cooper-Munro (BCM) curve [22], experiments using electrical stimuli [23], and Ca^2+^-induced plasticity [24]. Thus, model 0 was consistent with a number of experimental observations for which it had not been tuned. However, model 0 did not have the capacity to retain these output changes when the inputs returned to resting levels.

We then used model 0 to analyse AMPAR responses to sustained changes in four parameters that might act as sites of regulation of AMPAR trafficking. The parameters were the activities of CaMKII, PKA, and PP1, and recycling rates of receptor to and from surface membrane. Each of these parameters is implicated in synaptic change, and is a possible upstream control signal for AMPAR conductance. It is known that appropriate stimuli can lead to changes in synaptic conductance over a range of approximately 50% to 200% of basal synaptic transmission levels [25]. We asked which of these parameters could control AMPAR conductance over this range.

**Figure 4:**
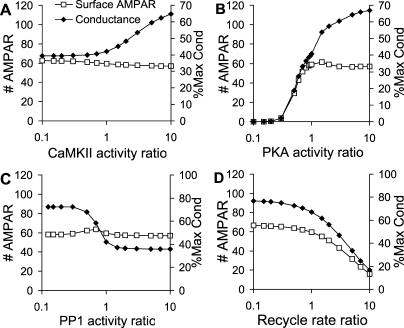
AMPAR Synaptic Membrane Localisation and Conductance in Response to Sustained Inputs Each panel is computed from a series of steady-state calculations where the activity of the selected input pathway was scaled with respect to its basal activity. The *x*-axis is this scaling ratio. The conductance is calculated as in Figure 3 and is expressed as the secondary *y*-axis, as a percentage of maximal conductance (see Materials and Methods). (A) Changing activity of CaMKII leads to small changes in synaptic membrane localisation of AMPARs, but phosphorylation of GluR1 on Ser831 gives a doubling of synaptic conductance when CaMKII activity is scaled above basal levels. (B) Low concentrations of PKA result in reduced exocytosis of AMPAR. Basal concentrations of PKA (ratio ~ 1) are required to localise AMPAR to the synaptic membrane, and higher concentrations cause a conductance increase. This occurs because of phosphatase saturation leading indirectly to a rise in Ser831 phosphorylation due to CaMKII. The net effect is that changes in PKA activity can lead to a large change in AMPAR conductance in either direction. (C) Changes in PP1 concentrations have little effect on AMPAR localisation. However, low PP1 leads to high phosphorylation of GluR1-Ser831 by CaMKII, and hence high conductance. (D) Lower rates of receptor recycling to the internal pool lead to a small increase in synaptic membrane localisation. High rates bring most of the receptor to the internal pool.

In our simulated experiment, we scaled each of the four parameters from 0.1 to ten times its basal value, one at a time. In the case of CaMKII, PKA, and PP1, this scaling was done by numerically buffering the level of the active form of the enzyme to the desired value. In the case of the recycling rates, we scaled endocytosis and exocytosis rates as described below. In each case, the concentrations of the remaining molecular parameters (CaMKII, PKA, and PP1) were allowed to settle to new steady-state concentrations. In biological terms, this would correspond to applying inhibitors or activators of the selected parameter. We recorded the resulting steady-state number of synaptic AMPARs (Figure 4). As in Figure 3, we also computed the AMPAR conductance as a percent of the maximal conductance. The maximal conductance is the conductance if all the receptors were in the membrane in the doubly Ser831-phosphorylated state. In each of these calculations we maintained the total number of internal plus synaptic membrane receptors at 80 molecules, and Ca^2+^ concentration was at its resting level of 80 nM.

The plots in Figure 4 show the results over the entire range of scaled active inputs, from a ratio of 0.1 to ten times the basal concentration of the input. CaMKII and PP1 (Figure 4A and 4C) had rather little effect on synaptic membrane localisation of receptor. Instead they acted in a complementary manner in changing synaptic conductance through phosphorylation and dephosphorylation of GluR1 on Ser831.

PKA (Figure 4B) had the largest total effect on synaptic conductance, spanning a range from nearly zero to a conductance of nearly 70% of maximal. At low PKA activity there was little insertion of AMPARs into the synaptic membrane, so the conductance was small. At high PKA activity, most of the receptors were inserted. Additionally, the PKA active input indirectly activated CaMKII, leading to receptor phosphorylation on Ser831, resulting in a further increase in conductance. This indirect activation occurs through two successive inhibitory steps. First PKA inhibits PP1 because phosphorylated inhibitor 1 of PP1 binds to and blocks PP1. Second, PP1 itself inhibits CaMKII by dephosphorylation of the kinase (see Figure 1E).

Receptor recycling has been suggested as a mechanism for altering synaptic conductance [9,10]. In the simulations we scaled the AMPAR endocytosis rate by the specified recycling ratio, and simultaneously the exocytosis rate by its inverse. Thus, a ratio of 0.1 would have an endocytosis rate of 0.1 times basal, and an exocytosis rate of ten times basal. Interestingly, it was not easy to drive more receptors into the synaptic membrane (Figure 4D). This was partly because the model already had most of its receptors in the membrane. At high values of receptor recycling rate, the endocytosis rate was greater than the exocytosis rate, so the amount of synaptic-membrane-bound receptor was strongly depleted.

Thus, as an initial prediction, our simulations pointed to either PKA or some combination of CaMKII, PP1, and recycling as being a sufficient long-term control signal to account for bidirectional AMPAR changes, even in a regime where receptor counts did not change. These control effects were not surprising, as these interactions with AMPAR recycling were specifically included into our model. Nevertheless, the model did illustrate the amount of AMPAR insertion or removal to be expected from different manipulations. A similar combination of regulatory inputs has been implicated in learning (e.g., [26]). However, the simulations at this stage did not address the question of how such long-term control signals might be maintained.

### AMPAR Bistability

The above explorations of model responses had suggested that the model was reasonably consistent with a range of experimental findings, including several that it had not been tuned for. However, these initial tests used model 0, which did not consider molecular turnover. How might the inclusion of receptor synthesis and degradation alter AMPAR trafficking?

In preliminary simulations (not shown) we added or removed AMPAR molecules from model 0 at the time of starting the model, and asked how the receptors redistributed when the model was run out to steady state. The AMPAR molecules were added to the doubly Ser845-phosphorylated internal pool of receptors, but the site of addition did not affect the final steady-state distribution. Unexpectedly, we found that the addition of receptors to model 0 actually decreased the number of native receptors. Native receptors are defined as unphosphorylated receptors in the endocytosed pool. This suggested that receptors were being redistributed in the model synapse in a manner that might lead to two stable states. We proceeded to test this using a series of simulations on the complete model including AMPAR synthesis and degradation, that is, model 1. Details of model 1 parameters are in Protocol S1.

**Figure 5:**
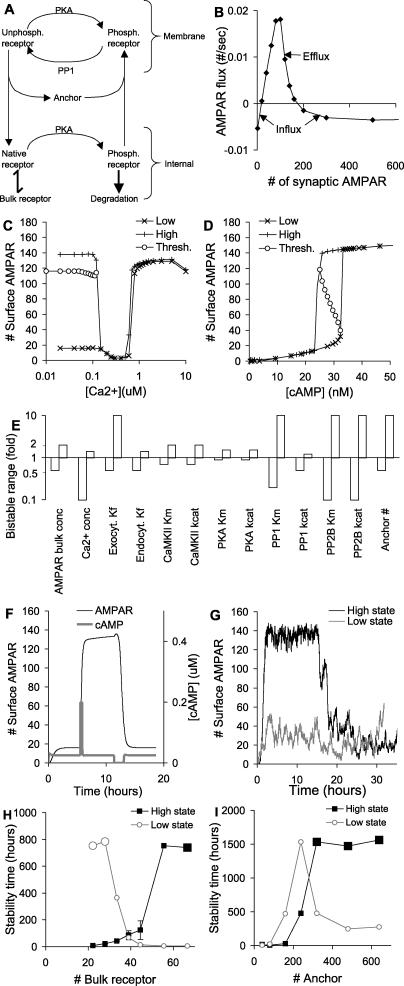
AMPAR Translocation and Bistability for Model 1 (A) Simplified schematic of receptor recycling. (B) Bistability analysis. The flux of AMPARs from the bulk AMPAR pool to the native AMPAR pool is plotted against the total number of synaptic receptors. Receptor influx into the spine occurs both at very low and at high numbers of synaptic AMPARs. (C) States of the system as a function of Ca^2+^ concentration. Upper curve is obtained by starting system in state with high numbers of AMPARs in the synapse; lower curve with low numbers of AMPARs. The intermediate threshold curve is calculated using successive bisection as described in the Materials and Methods. Bistability is present when Ca^2+^ concentration is less than 0.12 μM. Between 0.12 and 0.6 μM the system settles to a state of low AMPAR numbers. Above 0.8 μM the system is in the high state. (D) States of the system as a function of cAMP concentration. Steady-state number of AMPARs is calculated as in (C). There is hysteresis as the high and low states coexist for the bistable region of the curve. Threshold points (open circles) complete the characteristic S-shaped curve for a bistable system. (E) Parameter sensitivity analysis. The bars represent the range of parameter values over which the system remains bistable. Other than key regulators, the system tolerates a 2-fold or greater range of most parameters without losing bistability. (F) Time course of AMPAR showing two stable states. A pulse of 0.2 μM cAMP is applied for 1,000 s to trigger translocation of AMPARs to the synaptic membrane. Following this, cAMP is restored to resting levels, and the system settles to the state of high membrane AMPAR. The “off” stimulus is provided by reducing cAMP to 0 μM for 6,000 s. Following this, the system settles back to the basal state of AMPAR. (G) Stochastic runs in low and high states. The high state is triggered by an initial cAMP pulse from *t* = 0 to 4,000 s. The state spontaneously turns off at around 20 h in this run, but the low state does not flip. (H) Average stability time of low and high states for different numbers of bulk receptors (mean ± standard error of the mean). Twenty-four simulations for each state were run, as in (G). Stability time is calculated as total simulation time in selected state, divided by number of transitions out of that state. Large symbols represent cases where no transitions occurred over the entire set of simulations. As expected, a higher level of bulk receptor increases the likelihood that the spine will spontaneously turn “on”, and vice versa. (I) Stability time in low and high states for different numbers of anchor proteins. As the number of anchor proteins increases the stability time for both states also rises. At very large numbers of anchor proteins the synapse occasionally turns “on” spontaneously. Symbols and calculations as in (H).

In model 1 we assumed that newly synthesised AMPARs are present in the dendrite (referred to as bulk AMPAR), and that they exchange with the native receptors in the spine (Figures 1A, 1B, and 5A). We also assumed that there is a slow degradation of the doubly Ser845-phosphorylated receptor pool. Model 1 has 164 anchor proteins located in the PSD. We performed several tests on model 1 to examine whether it indeed exhibited two stable states with different numbers of receptors inserted into the synaptic membrane. We examined influx of receptors into the spine under different conditions. We then performed steady-state analyses, including a parameter sensitivity analysis, to show bistability. We simulated state changes of the model in response to stimuli and stochasticity. These results are described below and in Figure 5, and cumulatively characterise the bistable properties of the model.

We first asked whether there were two states in which there was an influx of receptors into the spine. This influx would be a necessary condition to offset degradation, and the presence of two such states would be an indication of bistability. We computed receptor flux between the dendrite and the native receptors in the spine as a function of the total number of AMPARs in the synapse (Figure 5B). We defined the total number of synaptic AMPARs as the sum of AMPARs in the internal synaptic pool and in the synaptic membrane (Figure 5A). In order to compute the flux we performed the following manipulation: AMPAR molecules were added to the doubly Ser845-phosphorylated internal pool of receptors. Receptor exchange with bulk AMPAR was disabled to allow the system to settle to steady state for 5,000 s without loss of receptors. Then receptor exchange was re-enabled and the simulation was run for a further 1,000 s to settle. The flux of receptors between bulk AMPAR and the native receptors was calculated at this time point to obtain Figure 5B. This calculation confirmed our preliminary observations. We found that there were two distinct and widely separated regions of receptor influx, one where there were fewer than 20 total synaptic AMPARs, and one where there were 180 or more.

This suggested that there were indeed two stable regimes where receptor influx from the dendrite might balance out receptor degradation. In the regime of low numbers of synaptic AMPARs, there was a simple equilibration between the bulk AMPA receptor pool and the native receptor pool. In the high-number regime, the receptors in the spine were redistributed to the membrane so that the native receptor pool was depleted, again leading to receptor influx. The presence of two regimes of receptor influx, depending on the number of synaptic AMPARs, may be an experimentally testable prediction.

To confirm that this formed a bistable system, we computed stable states of the system under different regulatory conditions (Figure 5C and 5D). We used Ca^2+^ and cyclic adenosine monophosphate (cAMP) as regulatory inputs. We obtained the stable states by running model 1 to steady state (120,000 s), starting from either a low state (low numbers of synaptic AMPARs) or a high state (high numbers of synaptic AMPARs). In cases where there was only one stable state, the two runs converged to the same steady value. In cases where there were distinct stable states, the two runs settled to their respective high or low stable states. We also found the unstable fixed point (the threshold for state switching) by using an iterative bisection method described in Materials and Methods.

These simulations give us dose-dependence curves that illustrate the bistable nature of the system. The Ca^2+^ dose dependence of the switch is interesting and unusual (Figure 5C). In the low Ca^2+^ regime, the system is bistable. The system settles into either a high or low state depending on initial conditions. In the 0.12 to 0.6 μM range, the system goes into a single low state of activity because of the action of calcineurin (PP2B). Calcineurin is activated at these concentrations of Ca^2+^, and is able to rapidly dephosphorylate AMPAR. The unphosphorylated receptor moves into the native receptor pool, and then out to the dendrite. At Ca^2+^ concentrations over 0.8 μM, the system goes into a single state of high activity because of CaM activation. The activity of CaM leads to increased AMPAR phosphorylation both through PKA and CaMKII. CaM-activated adenylyl cyclase (see Figure 1) produces cAMP, increasing PKA activity. CaM also directly activates CaMKII. These events are similar to those described for Figure 3.

In this manner, applied Ca^2+^ can flip the state of the switch in either direction, depending on Ca^2+^ amplitudes. The switch is unusual because both states are reached by an increase in the regulatory concentrations of Ca^2+^. As considered below for cAMP, it is much more common for one state to be triggered by low regulator concentrations, and the other state by high regulator concentrations. This bidirectional regulation by increases in Ca^2+^ is also observed in the findings shown in Figure 3A, but is not as striking. It is possible that there may be a very narrow bistable region at around 0.7 μM Ca^2+^ in the model, as suggested by the small separation between the low and high curves, but this was not within the numerical resolution of our calculations. We do not expect that such a fine separation would be biologically relevant. We also estimated the thresholds for the bistable switch (open circles in Figure 5C). These were not very dependent on Ca^2+^ concentrations. In biological terms, the synapse could be switched to the low state by raising Ca^2+^ to the low regime (0.12 to 0.6 μM), allowing the flux of receptors to be initiated, and rapidly lowering Ca^2+^ back to the bistable regime. To attain the high state, the Ca^2+^ input should be over 0.8 μM for long enough for the switch to settle, and then Ca^2+^ should rapidly fall below 0.1 μM into the bistable region. As we discuss later, the details of biological Ca^2+^ dynamics are beyond the scope of our current steady-state models.

The cAMP dose dependence of the switch was more conventional. It took the shape of a simple hysteresis curve, where the low state resulted from a decrease in cAMP and the high state from an increase (Figure 5D). The bistable region of the switch is in an intermediate range of cAMP. In this case the synapse switches to the low state if cAMP is reduced below 20 nM, and to the high state if cAMP is raised above 35 nM. To complete the analysis, we found the unstable fixed points of the bistable switch (open circles in Figure 5D). These points can be interpreted as thresholds for switching from one state to the other. As expected for a bistable system, these unstable fixed points curve back toward the limits of the hysteresis cycle in an S-shaped curve (Figure 5D).

How “robust” is the bistability of model 1? One measure of this is to ask whether the bistable effects persist when important model parameters are varied. We systematically varied important model parameters and looked for bistability. To test for bistability, we started the model off in either the upper or lower state, then ran it out to steady state with the altered parameters. If the model switched state it was no longer bistable. We found that model 1 retained its bistable behaviour over a wide range of most parameters, illustrated in Figure 5E. The key “sensitive” parameters were exactly those identified in Figure 4 as key regulators of steady-state synaptic conductance: CaMKII, PKA, recycling, and Ca^2+^. Most parameters were able to scale a factor of at least two up or down without losing bistability.

As a simpler signature of bistability we ran a time-course simulation in which the model explicitly switched between two steady states (Figure 5F). Here the model first settled to the low state where there were few AMPARs in the synaptic membrane. Following a cAMP pulse (0.2 μM, 1,000 s), the model switched to the high state, with many AMPARs inserted into the synaptic membrane. The system was switched back to the low state by numerically reducing cAMP to zero (6,000 s). While this switching of the number of membrane-inserted AMPARs is possibly a testable prediction, it only indicates the presence of bistability and does not shed much light on the mechanism, which is analysed below.

The time course of switching was slow, of the order of an hour. This was consistent with the receptor trafficking rates in the model, which were derived from steady-state measurements. More rapid transient rates may be applicable during synaptic stimulation, as we consider in the Discussion.

Another manifestation of robustness is the ability of the model to retain state information despite stochasticity. Stochasticity in the synapse originates from the probabilistic occurrence of reactions among small numbers of molecules and gives rise to apparent biochemical noise. This noisiness imposes severe constraints on the reliability of any proposed synaptic signalling mechanism [7]. In particular, bistable biochemical systems are subject to spontaneous state flips because of biochemical noise [7,17]. We tested stochastic responses by simulating model 1 using the Gillespie exact stochastic method [27]. The entire model was simulated stochastically, including all molecules in the dendrite, spine head, and PSD (details in Materials and methods). We started the model in the low state, where few AMPARs were inserted into the synaptic membrane (Figure 5G). In half the runs we applied a cAMP stimulus to switch the model to the high state at around 1 h (black line in Figure 5G). We then simulated the model for a period of 120,000 s (>33 h) to test its resistance to spontaneous switching from either state. In the example in Figure 5G, the low state (gray line) did not change, but the high state (black line) spontaneously turned off at around 16 h.

Based on our analysis shown in Figure 5B, the stability of each state should be a function of the concentration of bulk AMPAR. If the concentration of bulk AMPAR is high, then receptor influx increases. Under these conditions, a relatively small fluctuation should push the system past the efflux regime into the upper influx regime (Figure 5B). Conversely, at low bulk AMPAR it should be easy to flip from the high to the low state. We repeated our stochastic simulations for a range of bulk AMPAR concentrations while otherwise retaining the parameters of model 1. At each concentration of bulk AMPAR we repeated the simulations at least 24 times to build up a profile of the switching times (Figure 5H). At our reference range of 11.11 nM of bulk AMPAR, the off state was stable for more than 360 h on average, and the on state was stable for about 42 h. As we discuss below, other synaptic processes may take over the job of maintaining state information, within this time frame.

At low bulk AMPAR the high state was very short-lived, but the low state did not flip at all during the entire duration of our simulations (indicated by the large symbols in Figure 5H). Conversely, at high bulk AMPAR, the low state was unstable but the high state lasted for very long times.

We then considered how the lifetime of model states might scale with the number of anchor proteins in the PSD. As the number of anchor proteins sets the maximum number of receptors that can be inserted into the membrane, this parameter is important for the robustness and stability of the model. We simulated the stability time of the switch for a range of anchor protein numbers (Figure 5I). The default number of anchor proteins in the model was 164. At lower anchor protein numbers the switch lifetime was rather short, of the order of a few hours. At higher anchor numbers the switch lifetime increased rapidly for both states. The lifetime of the high state continued to rise and exceeded 2 mo when there were more than 320 anchor proteins (large symbols in Figure 5I indicate that the model did not change state during the entire duration of our simulations). The lifetime of the low state was greatest (approximately 2 mo) at 240 anchor proteins, and then declined to around 200 h when more anchor proteins were present. This may have occurred because the presence of additional anchor proteins biased the movement of the receptor toward the spine. Biologically, an increase in anchor protein number may correlate with the size of the spine head. Thus, our model predicts that larger spines should be more stable, an observation that has some experimental support [28,29].

At this stage of the study we had extensively analysed the properties of the bistability of model 1 with respect to the number of AMPARs at the synapse. We had considered state dependence on flux and regulators. We had shown that the bistability was robust, and in particular had shown how long the model could retain state information when stochasticity was taken into account. Based on these calculations, we suggest that the model might be a candidate for retaining synaptic state information for hours to months.

**Figure 6:**
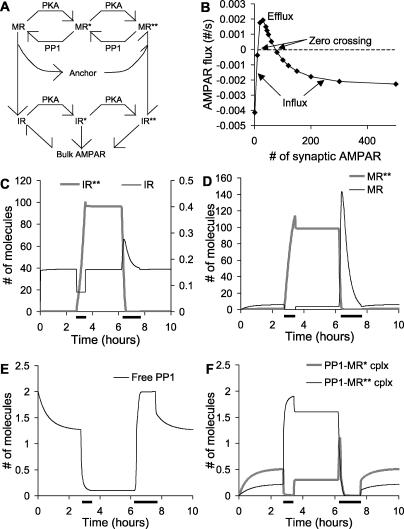
Simplified AMPAR Bistable Model IR represents internal receptor, MR represents synaptic-membrane-localised receptor and asterisks indicate phosphorylation at Ser845. PKA is protein kinase A. (A) Complete reaction diagram. (B) Bistability analysis for simplified model. AMPAR flux between the bulk AMPAR and the IR state from (A) is plotted against the total number of synaptic receptors (IR + IR* + IR** + MR + MR* + MR**). As seen in the complete AMPAR model in Figure 5B, there are two regions of receptor influx into the spine, at low and high numbers of synaptic AMPARs. The zero crossings are stable states where there is no net flux of receptor. (C–F) Time courses of key molecules in simple model. After an initial settling period, PKA is raised to 40 molecules for 2,400 s to trigger receptor influx. After the system settles into the high state, PKA is set to zero molecules for 3,600 s, to return the system to basal levels. These stimuli are indicated by horizontal bars along the time axis. (C) Internal receptor numbers. The number of receptors in the unphosphorylated form (IR) remains very close to the bulk receptor level except during transitions, when receptors enter or leave the system. (D) Synaptic-membrane-bound receptor levels. (E) Numbers of free PP1 decline sharply during the high state, because of enzyme saturation. (F) Numbers of PP1 complexes with substrates. The high amount of PP1–MR** complex is complementary to the decline in free PP1, showing the saturation of the phosphatase.

How does the bistability arise? Model 1 is quite complex and involves 16 phosphorylation states of AMPAR each in the internal pool and in the synaptic membrane. For simplicity, we made a skeletal model with the same general topology. The skeletal model retained only two phosphorylation/dephosphorylation steps involving AMPAR (Figure 6A). This is called model 2. We used the same parameters as for the full model, with the exception of lower concentrations of PP1 (0.333 μM). The concentration of PP1 was reduced as its other substrate, CaMKII, was not present in model 2. The *K*_m_ for PKA and PP1 was halved as compared to model 1, as each receptor phosphorylation site in model 2 corresponds to two receptor phosphorylation sites in model 1. For model simplicity we had the degradation steps feeding into the bulk AMPAR pool, which was numerically buffered to a steady value. The full parameters for this model are presented in Protocol S1. We used this simpler model to analyse the mechanistic basis of this form of bistability.

We repeated the analysis of receptor flux as a function of the number of total synaptic receptors. Model 2 also had two regions of AMPAR influx separated by a region of efflux from the synapse (Figure 6B). This indicated that it shared the same mechanism for bistability as model 1. We then performed a simulation of the time course of stimulus-triggered transitions between the stable states of the model. We took advantage of the smaller number of molecular species in model 2 to monitor all the molecular concentrations during PKA-triggered transitions between the low and high states. In this simulation the steady-state amount of PKA was one molecule. The switch was turned on using a stimulus of 40 active PKA molecules for 2,400 s, and turned off using zero active PKA molecules for 5,000 s. The responses of several molecules are illustrated in Figure 6C–6F. The low stable state (0 to 3 h) is characterised by low numbers of all forms of the receptor, and consequently little saturation of PP1. During the switch to the high state at 3 h, the concentration of unphosphorylated internal receptor drops, leading to a large influx of receptors. These are rapidly phosphorylated by the high numbers of PKA, and the number of IR** (see Figure 6 legend for explanation of abbreviations) rises sharply.

As the IR pool exchanges rapidly with bulk AMPAR, its concentration rapidly returned to basal levels after the PKA stimulus ended. Once in the IR** state, the receptor translocated to the synaptic membrane, into the MR** pool. Due to the large numbers of MR**, the PP1 became saturated (Figure 6E and 6F). Thus, the combination of PKA phosphorylation of MR, and translocation from the IR** pool, formed MR** at a greater rate than the PP1 could dephosphorylate it.

At 7 h we switched the system back to the low state by removing all PKA. This allowed PP1 to complete dephosphorylation of the phosphorylated receptor pools. There was a large transient rise in MR due to the dephosphorylation of MR** and MR*, and the slow traffic back to the IR pool. Finally, when we restored active PKA to its resting level the system settled back into the lower stable state.

The translocation step appears to be important—we were unable to obtain bistability without it—but it was not possible to completely explore parameter space so it was not clear whether this is an absolute requirement. We were also able to obtain bistability with a single phosphorylation step, provided that the translocation step was second order in the receptor (data not shown). In all these processes, it was assumed that the bulk AMPAR was constant, that is, that the balance of synthesis and degradation was sufficient to rapidly add and remove receptors from the spine. In the biological system the situation is more complex and synthesis itself may be activity dependent [30–32].

In summary, the simple model retains the fundamental features of the AMPAR translocation-based bistability and facilitates an analysis of its mechanism. The low state of this form of bistability occurs when few synaptic AMPAR molecules are present, so that PKA can act on only a few substrate molecules, and PP1 is not saturated. Therefore, few internal AMPARs are in the phosphorylated state and only a few AMPARs are translocated to the surface. The upper state of activity is characterised by high numbers of AMPARs in the phosphorylated states both internally and in the synaptic membrane. This state persists because of the higher basal activity of PKA as compared to PP1. This leads to PP1 saturation. The final step in maintaining the upper state is the translocation of phosphorylated receptor to the membrane. This removes receptors from the internal pools and keeps the native receptor (internal receptor) at sufficiently low levels that receptor influx is favoured. A similar PP1 saturation effect is seen in the complete model (model 1) when it is in the high state (data not shown). This is a possible testable prediction of the model.

### CaMKII Bistability

**Figure 7:**
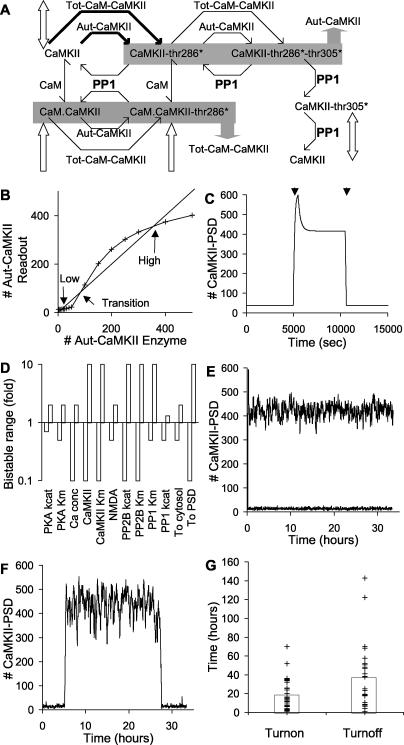
Bistability in the CaMKII Model CaMKII-thr286* indicates Thr286-phosphorylated CaMKII, and CaMKII-thr286*-thr305* indicates Thr286/Thr305-phosphorylated CaMKII. (A) Schematic of CaMKII autophosphorylation in the PSD. Reactions (in bold) and PP1 concentrations are altered in the PSD from the basal model to achieve bistability. Block arrows indicate the CaMKII states that translocate. There are two shaded sets of molecules, indicating states that are summed to give rise to an enzyme activity. The Tot-CaM-CaMKII activity is the sum of concentrations of CaM-CaMKII and CaM–Thr286-phosphorylated CaMKII. The Aut-CaMKII activity is the sum of the calcium-autonomous states Thr286-phosphorylated and Thr286/Thr305-phosphorylated CaMKII. (B) Bistability analysis. The Aut-CaMKII molecular species has been bifurcated into Aut-CaMKII enzyme, and Aut-CaMKII readout. The enzyme activity is buffered numerically (*x*-axis). The readout (*y*-axis) remains the sum of Thr286-phosphorylated and Thr286/Thr305-phosphorylated CaMKII. Fixed points are given by the intersection points of the amount of Aut-CaMKII with the 45° line. These fixed points indicate the number of Aut-CaMKII molecules that would exactly sustain their own activity through autophosphorylation. The upper and lower points are stable, and the middle point is the transition point. (C) Time course of bistable response. The first arrow is a Ca^2+^ stimulus of 2.7 μM for 500 s that switches on the CaMKII bistable loop. The second arrow is a 5-fold increase in *k*_cat_ of PSD-localised PP1 for a period of 500 s, which switches CaMKII off. (D) Parameter sensitivity analysis. Key parameters are scaled up and down and the model is tested for bistability. Most parameters can be varied 2-fold or more in either direction without the model losing bistability. (E) Stochastic run showing stability in both high and low states, when PKA is buffered. (F) Stochastic run showing spontaneous state flips in either direction, with the complete PKA model. (G) Statistics of spontaneous state flips with the complete PKA model. Average turn on and turn off times are both over 15 h.

Having characterised AMPAR translocation bistability, we wished to examine the well-studied CaMKII autophosphorylation system in the context of translocation. Although model 1 included CaMKII translocation, the CaMKII portion of the model was not bistable. A key assumption for CaMKII bistability is that upon binding to NMDAR, the CaMKII becomes susceptible to autophosphorylation even in the absence of bound CaM [33]. In our model, as in others (e.g., [34]), this assumption is also linked to PP1 saturation as we now describe. In order to analyse the CaMKII responses in a simpler context, we derived a reduced model from the basic model discussed above. This reduced model contained only CaMKII in the cytosol and PSD, and its immediate regulators, PP1, CaM, and PP2B. PKA was included only as the active enzyme, without regulatory steps. We modified this reduced model of CaMKII by including the autophosphorylation in the absence of CaM. These changes are illustrated by the dashed line in Figure 1C and the bold lines in Figure 7A. This model is called model 3.

Despite the simplicity of our CaMKII model as compared to previous work [34,35], we were able to obtain bistability. To do so we used somewhat different phosphorylation and dephosphorylation rates in the PSD as compared to the model 1 (see Protocol S1). Since CaMKII phosphorylates itself, we had to adapt our previous analysis, which relies on separate input and output molecules [4]. We did this by numerically bifurcating the autonomously active CaMKII–PSD (Aut-CaMKII; Figure 7A) into two simulated molecular pools: Aut-CaMKII enzyme and Aut-CaMKII readout. We set the number of Aut-CaMKII enzyme pools to specified values, and monitored the number of molecules in the Aut-CaMKII readout pool (Figure 7B). This manipulation was facilitated because the level of Aut-CaMKII is computed as the sum of the autonomously active states of Thr286-phosphorylated and Thr286/Thr305-phosphorylated CaMKII, indicated in gray in Figure 7A. So our enzyme assignment bypassed this summation, and directly set the number of Aut-CaMKII molecules. Our readout number was the sum of Thr286-phosphorylated and Thr286/Thr305-phosphorylated CaMKII.

The results of these calculations for a range of Aut-CaMKII enzyme values are shown in Figure 7B. The intersection points of this curve with the 45° line are stable points of the system, because at these points the enzyme and readout activities of Aut-CaMKII are identical. In other words, at these points the autonomous CaMKII would exactly sustain its own activity. The upper and lower intersection points define the stable numbers of Aut-CaMKII, and the intermediate point is a transition point. This behaviour can be seen by considering a small increase in the number of Aut-CaMKII enzyme above one of the stable points. The resulting Aut-CaMKII readout (read off from the *y*-axis) would be smaller than the new input number. This would tend to restore the CaMKII activity toward the stable point. A similar argument applies to small negative deflections from the stable points. Around the transition point the situation is reversed: any small deflection will be amplified until the system converges to either the upper or lower stable point.

We confirmed the presence of bistability by simulating a time series in which the system was turned on with a calcium pulse of 2.7 μM for 500 s and later turned off by raising the *k*_cat_ of the PSD-localised PP1 by 5-fold for 500 s (Figure 7C). Two distinct stable states were observed, which corresponded to the upper and lower intersection points in Figure 7B. There is a small offset between the two calculations because Figure 7C reports all forms of CaMKII in the PSD, whereas Figure 7B refers only to Aut-CaMKII.

We evaluated the robustness of the CaMKII model (model 3) using the same approach as for the AMPAR model (model 1). We found that the CaMKII bistability was highly robust with respect to variation of parameters (Figure 7D). Many parameters could be varied from 0.1 to ten times the reference value without losing bistability. Most of the remaining parameters could be varied from 0.5 to two times the reference value, and only PP1 and PKA were more sensitive. This sensitivity reflects the key role of PP1 in dephosphorylating CaMKII, and the role of PKA in controlling the activity of PP1.

We checked the robustness of model 3 in synaptic volumes by simulating it using stochastic numerical methods. Model 3 was resistant to spontaneous switching and we did not observe any switches to either state in over 300 cumulative hours of simulation time. A 33-h sample of the high and low states is shown in Figure 7E. This state stability turned out to be an artefact of our reduced model for CaMKII, which only used the final active concentration of PKA as one of the key regulatory inputs. The PKA pathway model output was quite noisy in small volumes [36]. When we incorporated the full PKA pathway into model 3, we found it introduced a considerable amount of additional stochasticity into the system and did lead to bidirectional state flips (Figure 7F). We repeated these simulations 50 times and found that the off state endured for 17.3 ± 2.5 h, and the on state for 37.2 ± 6.9 h (Figure 7G). Thus, there may be a marked reduction in bistable state lifetimes when noisy inputs are taken into account. This issue is considered in the Discussion.

Overall, our CaMKII translocation model (model 3) also exhibited bistability coupled with translocation, such that the active state led to accumulation of CaMKII in the PSD. Under stochastic conditions, the lifetime of stable states in the model was sensitive to noise from the PKA input pathway.

### Bistability Interactions: Tight Coupling

**Figure 8:**
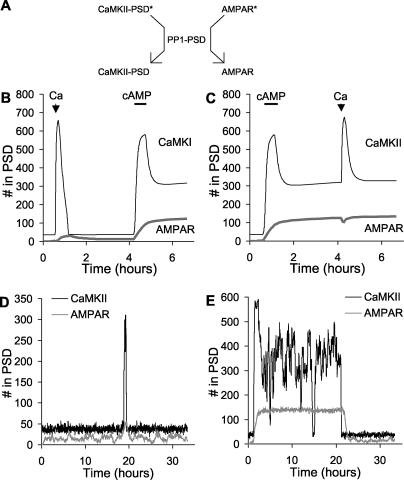
Bistability for Tightly Coupled Switches (A) Schematic of PSD-localised PP1 acting on both CaMKII and AMPAR substrates in the PSD. The asterisks on CaMKII and AMPAR represent phosphate groups. (B) Time course of response to Ca^2+^ (2.7 μM, 500-s duration), then cAMP (0.108 μM, 2,000-s duration) stimuli. The initial Ca^2+^ stimulus turns on CaMKII transiently, but it eventually returns to baseline. The subsequent cAMP stimulus turns on both switches. (C) Time course of response to cAMP (0.108 μM, 2,000-s duration), then Ca^2+^ (2.7 μM, 500-s duration) stimuli. The initial AMPAR stimulus (cAMP elevation) is sufficient to turn both the AMPAR and the CaMKII switches on. (D) Stochastic run in the low state. The figure illustrates a transient event that did not result in complete turn on. (E) Stochastic run in the high state. There is a spontaneous turn off, but the average on time is over 100 h.

At this point we had a reasonably constrained model of AMPAR and CaMKII trafficking, and had shown that under certain conditions both molecules could be bistable. In the final part of the study we considered interactions between these two forms of bistability. We first made model 4 by merging model 1 and model 3, while sharing the same PP1 molecule in the PSD (Figure 8A). This scenario assumes that the PP1 is free to move between its CaMKII and AMPAR substrates while remaining in the PSD. Thus, there is a tight coupling between the two forms of bistability, mediated both by PP1 and by CaMKII phosphorylation of Ser831 of AMPAR, as in model 1. We asked how the system would respond to stimuli designed to activate the AMPAR and CaMKII switches independently.

In our first test we stimulated the model 4 with Ca^2+^ (2.7 μM, 500 s) then allowed the system to settle for 3 h, then stimulated it with cAMP (108 nM, 2,000 s) (Figure 8B). Following the Ca^2+^ stimulus, the CaMKII switch turned on transiently, but soon returned to baseline. The AMPAR switch did not turn on until the cAMP stimulus was applied, and at this time CaMKII also turned on. When the cAMP stimulus was applied first, it rapidly turned on both the AMPAR and CaMKII switches (Figure 8C). Together, these simulations show that in model 4 the two forms of bistability function in lockstep. That is, sustained activation of CaMKII is contingent upon the activity of AMPAR. If AMPAR is activated, it saturates PP1, and this leads to activation of the CaMKII bistable switch. As shown in Figure 8C, CaMKII is also activitated by the cAMP stimulus independently of the PP1-mediated cross-activation, leading to rapid turn on. This is because cAMP activates PKA, which relieves the PP1 inhibition of CaMKII. We ran separate simulations (not shown) that showed that even in the absence of this cAMP activation of CaMKII, the activation of AMPAR caused CaMKII to turn on as well.

We ran model 4 using stochastic methods to test its propensity to spontaneously change state. The off state was very stable though it did exhibit occasional transient spikes of activity (Figure 8D). The model spontaneously turned on only once in a cumulative total of over 1,000 h of simulation time. As before, we tested high-state durations by applying an initial cAMP stimulus at about 1 h to turn the system on, and then ran the simulation for about 33 h. We repeated these runs 24 times to obtain the distribution. The high state was not as stable, and was subject to occasional spontaneous flips to the off state with an average time of 101 ± 79 h (mean ± standard error of the mean). As expected from the lockstep mechanism, both CaMKII and AMPAR underwent a state flip at nearly the same time (Figure 8E).

### Bistability Interactions: Weak Coupling

**Figure 9:**
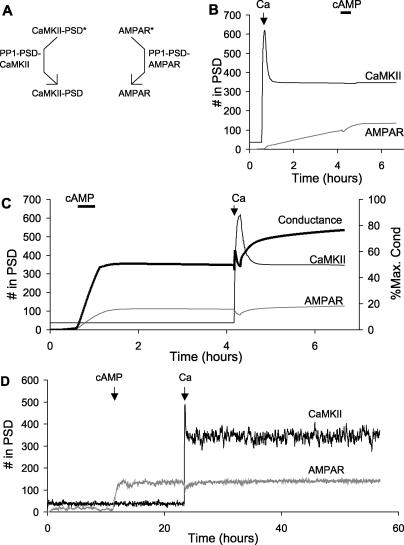
Nested Bistability for Weakly Coupled Switches (A) Schematic of independent PSD-localised PP1 enzyme activities for CaMKII and AMPAR. The two PP1 activities are labelled PP1-PSD-CaMKII and PP1-PSD-AMPAR, respectively. The asterisks represent phosphorylation. (B) Time course of system response to Ca^2+^ (2.7 μM, 500-s duration), then cAMP (0.108 μM, 2,000-s duration) stimulus. The initial activation of CaMKII leads to a slow turn on of the AMPAR system. (C) Time course of system response to cAMP (0.108 μM, 2,000-s duration), then Ca^2+^ (2.7 μM, 500-s duration) stimulus. First the AMPAR system turns on, then, following the Ca^2+^ stimulus, the CaMKII turns on. The conductance of the synapse has different levels in each of these states. (D) Stochastic run for 60 h, showing resting, AMPAR only, and AMPAR + CaMKII activity states.

In our final model (model 5), we considered the situation where CaMKII and AMPAR interacted only weakly. This is in contrast to model 4, where CaMKII and AMPAR were tightly coupled through a shared pool of PP1. In model 5, we combined the CaMKII and AMPAR bistable models (models 1 and 3) while keeping an independent pool of PP1 for each (Figure 9A). Such a scenario might arise if PP1 were bound to distinct scaffold proteins for each of its targets, and were restricted in its mobility across targets. The CaMKII-coupled pool of PP1 was treated as independent of PKA activity. There was one indirect form of coupling still present from CaMKII to the AMPAR bistability, since CaMKII phosphorylates AMPAR on Ser831. While this does not alter traffic rates directly, the Ser831 is a substrate for the AMPAR-associated pool of PP1 in model 5. Thus, CaMKII activity did contribute to the saturation of the AMPAR-associated PP1.

As before, we examined the interactions between the CaMKII and AMPAR switches using stimuli designed to turn each switch on independently of the other. When we first turned on the CaMKII switch alone using a Ca^2+^ stimulus, we observed a slow activation of the AMPAR switch (Figure 9B). The Ca^2+^ stimulus indirectly increased AMPAR insertion through the following steps: Ca^2+^ → CaM → CaMKII → phosphorylation of Ser831 → saturation of AMPAR-specific PP1 → reduced endocytosis of AMPAR. We did not model any changes in recycling or internalisation rates due to receptor phosphorylation on Ser831.

A particularly interesting effect was seen when the AMPAR switch was activated first using cAMP (Figure 9C). This stimulus turned on the AMPAR switch without affecting the state of CaMKII. At about 4 h we applied a Ca^2+^ stimulus that turned on the CaMKII switch as well. Thus, in model 5, the two switches were able to coexist in three combinations of states: both off, only AMPAR on, or both on. The fourth possible combination, of CaMKII on and AMPAR off, was not stable because of weak coupling between CaMKII and AMPAR, which slowly turned the latter on as shown in Figure 9B. The weak coupling is due to the phosphorylation of GluR1 on Ser831 by CaMKII. While this does not directly affect translocation, it does engage the AMPAR-specific pool of PP1, leading to eventual phosphatase saturation and turn on of the AMPAR switch.

Overall, this model exhibited nested bistability. The fundamental switch took place when AMPAR turned on or off. Nested within this was the capacity for CaMKII to turn on or off. A possible physiological outcome of the nesting of CaMKII activation is that the phosphorylation state and hence the conductance of the synapse can settle to three levels: (1) no AMPAR, (2) AMPAR with low levels of Ser831 phosphorylation, and (3) AMPAR with high Ser831 phosphorylation and consequently a higher conductance (Figure 9C). As shown in Figures 3 and 4, the equivalent conductance is expressed in terms of the number of unphosphorylated AMPAR channels that would have the same conductance.

The three states of the model were quite stable under stochastic conditions (Figure 9D). The only state that showed any transitions over the entire cumulative duration of simulations tested was that in which AMPAR was on and CaMKII was off (time to switch was 24.8 ± 3.5 h). We were particularly interested in the long-term stability of the states with both switches off, and both switches on. To examine these we performed several hundred independent stochastic simulation runs on a cluster, each representing 120,000 s (33 h) of simulated time. No state transitions were observed in either direction, over a cumulative duration of over a year of simulated time for each state.

### Transient Responses of Models

How does the model respond to stimuli that induce changes in synaptic efficacy? We tested two synaptic plasticity protocols on each of the models 1, 3, 4, and 5 (Figures S1 and S2). These tests were only qualitative, as the models in the current study were parameterized using steady-state rather than transient experiments. Nevertheless, they are useful in showing model behaviour under transient conditions. The first protocol had been used to elicit long-term potentiation (LTP) of synaptic efficacy and consisted of three bursts of 100 impulses at 100 Hz, each separated by 600 s. The second protocol was used to induce long-term depression (LTD) at the synapse, and consisted of 900 impulses at 1 Hz. We represented each stimulus as a computed calcium transient with an exponential build-up and decay of Ca^2+^ using the formulation of Zhabotinsky [34] (Figure S1). The LTP stimulus gave calcium peaks of 12 μM, and the LTD stimulus had peaks of 0.5 μM. We found that the LTP stimulus was able to cause a switch to the on state only in the CaMKII model (model 3) that incorporated the PKA activation pathway (Figure S2). This is interesting, as it suggests that even in its current form the CaMKII model is reasonably sensitive to physiological stimuli that may play a role in synaptic plasticity. The LTD stimulus did not turn off any of the models, indicating that the current models are missing some key interactions. These tests highlight some of the unknowns in our models, in particular, the specific transient interactions that are needed to trigger the steady-state effects we have analysed. As discussed below, more experimental detail will be needed to extend the models to include transient response characteristics.

### Summary of Bistable Behaviour

In summary, we analysed four bistable synaptic trafficking models (models 1, 3, 4, and 5). The remaining two models in this study (models 0 and 2) were used to characterise model 1. Model 3 included CaMKII alone, but models 1, 4, and 5 included both AMPAR and CaMKII. As described above, we performed a number of steady-state and transient tests to characterise the stable states of each model. Properties of these models are summarised in Table 1.

**Table 1:**
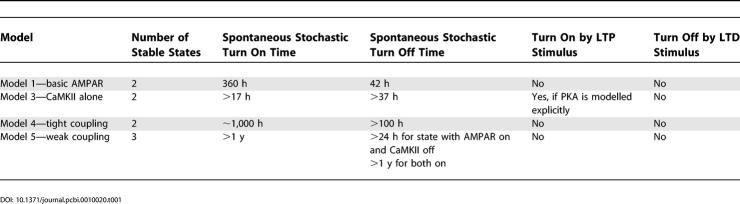
Summary of Model Properties

## Discussion

In this study we have developed a model of the movement of a glutamate receptor (AMPAR) and a calcium-activated kinase (CaMKII) to and from the synaptic membrane, using steady-state trafficking rates as a major experimental constraint. We find that the AMPAR trafficking cycle may lead to a form of switching or bistability where the presence of sufficient receptors at the synapse leads to recruitment of more receptors. This process may be a candidate for the transition from silent to active synapses, and early phases of their subsequent maintenance. When combined with a previously proposed mechanism for a CaMKII molecular switch, we observe interesting interactions between these two forms of synaptic bistability. Depending on the degree of coupling between these switches, we predict that AMPAR recruitment and CaMKII activation may either occur in a tightly coordinated manner, or nearly independent of each other. The latter may give rise to multiple stable synaptic states. Stochastic calculations suggest that these stable states persist for many hours, and in some cases over a year, despite biochemical fluctuations due to small numbers of molecules in the synapse.

### Structural Bistability

Long-term storage of information at the synapse is intimately connected to structural changes [8,15]. Such changes arise from the insertion, removal, and reorganisation of synaptic molecules. For example, many types of glutamatergic synapses initially lack postsynaptic AMPARs and are unresponsive to moderate synaptic input. These “silent synapses” become active when AMPARs are inserted. The change from silent to active synapses is a major mechanism for increases in synaptic efficacy [8]. Similarly, formation of dendritic spines is facilitated by the presence of the GluR2 subunit of the AMPAR [29]. Several signalling events are known that may affect these structural processes, but it is not always clear how persistent changes may be maintained. A single brief pulse of insertion of molecules, or formation of a synaptic spine, will have a transient effect unless some self-sustaining mechanism is also available to keep the changes in place. Formally, bistable systems are a possible mechanism for such structural memory, as such systems can withstand molecular turnover [2]. The AMPAR model analysed in the current paper shows that synaptic membrane insertion (a form of structural change) can be self-sustaining even when molecular turnover and noise are considered.

There are several proposed forms of synaptic bistability, and each has some structural correlates. The classical form of synaptic bistability is the CaMKII autophosphorylation system (reviewed in [3]). This is a biochemical bistability that relies on autophosphorylation leading to self-activation of CaMKII. This self-activation leads to bistability if PP1 is present at sufficiently low levels that it can be saturated. The involvement of PP1 saturation is also an important aspect of our model for AMPAR bistability. CaMKII activation has several structural correlates, including translocation to the PSD and formation of complexes with NMDA and other PSD proteins [33]. There is also recent evidence that CaMKII activation leads to an increase in AMPAR numbers [37]. Another synaptic biochemical bistability involving a MAPK feedback loop has been proposed [4] and tested in a fibroblast model system [38]. MAPK is known to be important in synaptic plasticity and may also play a role in structural changes at the synapse [39,40]. However, the MAPK bistable feedback mechanism is vulnerable to biochemical noise and may not be plausible in synaptic volumes [7]. A more recent proposal for a self-sustaining synaptic plasticity mechanism involves the mTOR system and local protein synthesis. mTOR phosphorylation increases protein synthesis at the dendrites, and the synthesis machinery itself is one of the products. Several other proteins have also been identified that may participate in such a feedback loop [6]. Such a protein-synthesis-dependent bistability would be very interesting for structural change at the synapse, as it could account for increased availability of many synaptic and PSD proteins.

In the current study we propose a novel form of synaptic bistability involving self-recruitment of AMPARs to the synapse. The mechanism is particularly interesting for the synapse in three ways: (1) it has parallels with the conversion of silent to active synapses, (2) it works at basal levels of activity of synaptic enzymes, and (3) it intimately involves a translocation and synaptic membrane insertion process. As analysed in Figures 5 and 6, this form of bistability is a function of the number of molecules of receptor in the synapse, rather than biochemical activation. The bistability involves phosphatase saturation of PP1 due to its action on AMPAR in the PSD. This is a specific prediction of this study. However, the prediction of AMPAR-specific PP1 activity is yet to be tested in detail. We discuss the issue of PP1 access to other substrates below.

It should be stressed that this mechanism for synaptic state maintenance is by no means exclusive. We discuss several other possible mechanisms above. There are also important details about AMPAR cycling that invite further analysis. For example, the AMPARs in Ser831/Ser845 double phosphomutant mice would not sustain this form of bistability. Nevertheless, these animals form synapses and retain some degree of synaptic plasticity [12]. Furthermore, our model considers activity-dependent changes in GluR12, and does not account for a presumed hand-over of synaptic state to some long-term process involving GluR23 insertion. Mechanisms for maintenance of GluR23 levels are still poorly understood, but we speculate that this too involves a self-recruiting bistable process.

Phosphatase saturation is a key part of our self-recruitment model. This has parallels with a distinct form of bistability analysed for the MAPK system by Markevich et al. [41]. In this MAPK model, there are two stable states of MAPK activity. The high state is sustained in part because of the saturation of phosphatases that reverse its activity. The distinction, again, is that the AMPAR bistability involves translocation without sustained biochemical activation whereas the Markevich model involves biochemical activation without intrinsic structural effects.

In a broader context, the AMPAR structural bistability could be generalised as a state-dependent translocation of molecules coupled to a saturable interconversion between these states. Many cellular trafficking events have a similar general form, including the Rab-mediated system of small GTPases and nuclear transport control. By our analysis in Figures 5 and 6, translocation bistability should exhibit two clearly separated regimes in which traffic occurs into one of the compartments, and a regime in which addition of the translocated molecule actually decreases its number in one of the cellular compartments. This might be an experimentally accessible signature for such behaviour in the cell.

### Stochasticity and Robustness

The typical synapse has a volume of 0.1 fl [16], and contains rather small numbers of key signalling molecules. This introduces fluctuations in reactions taking place in the synapse. We have previously analysed small-volume signalling for several pathways using simple assumptions about scaling and diffusion, and find that stochastic effects are so severe that some conventional signalling mechanisms simply do not work in these volumes [7,36]. Stable retention of synaptic state is a potential victim of stochasticity, as biochemical noise can lead to spontaneous state transitions. Bialek [17] and Miller et al. [42] have previously analysed synaptic bistability and suggest that in principle an autophosphorylation mechanism can give molecular stability of the order of hundreds of years even with a small number of CaMKII holoenzymes. Our current study is both coarser and more detailed than these analyses. Our representation of CaMKII does not consider individual holoenzymes, but on the other hand we explicitly represent translocation and several important signalling interactions at the synapse. In some models, and for some states, we did not observe any transitions over a year of simulation time. For other states the predicted lifetime is of the order of tens of hours (Table 1). Thus, in terms of state stability, our models are quite robust.

While it is heartening that our models are stable for many hours, it is clear that this may change in either direction if additional interactions are considered. For example, we ignore the diffusive exchange of most spine molecules with the PSD and dendrite. Over the time scales of our simulations, all the spine molecules would in fact exchange or degrade. This introduces further challenges to synaptic stability mechanisms that are beyond the scope of this study. A further illustration of the importance of context on stability comes from the CaMKII analysis in Figure 7. Here the inclusion of a more detailed PKA signalling model reduces the predicted lifetime of CaMKII states from hundreds of hours to tens. Additional input pathways may contribute to increased noise, but scaffold anchoring of enzymes such as PKA may create local signalling environments that could potentially reduce stochastic effects. Direct experimental measurements of synaptic stochasticity are difficult to perform [43], but such measurements would be invaluable for comparison with our simulated estimates of stochasticity.

Another commonly used measure of robustness is to ask whether the model retains some critical attribute over a wide range of parameters. In our analysis, we use bistability as the attribute of interest. We perform parameter sensitivity analyses by varying key reaction rates and enzyme parameters over a 100-fold range. Our parameter sensitivity analyses for the AMPAR and CaMKII systems suggest that our models are fairly robust by this measure. Most parameters can be varied at least 0.5- to 2-fold their original values without the model losing bistability (see Figures 5 and 7).

A particularly stringent measure of the robustness of a mechanism is to see how well essential features are preserved as reaction details are changed. We have performed a rather severe pruning of the AMPAR bistable model to a bare skeleton (see Figure 6) and with very little parameter tuning the simplified model is also bistable. Thus, by several measures, the bistable processes we have analysed are resistant to stochasticity, parameter uncertainty, and even changes in reaction mechanisms.

### Coupled Bistable Switches

Individual synapses are likely to exist in many states [18]. Given the short life of synaptic molecules discussed above, it seems possible that one mechanism for stabilising such states might be to associate bistable switches with them. Multiple states may be achieved if the individual switches are coupled loosely, so that combinations of states become possible. Here we have shown (model 5; Figure 9) that distinct forms of bistability may coexist to give rise to three possible synaptic states. The AMPAR switch is the major one, as it brings the receptors to the synaptic membrane in the first place. The CaMKII switch is nested within this as it can fine-tune the conductance of the receptors. This situation of nested bistable states is possible if the mobility of PP1 at the synapse is limited so that each PP1 molecule has exclusive access either to CaMKII or to AMPAR. The alternate assumption (model 4) is that PP1 is mobile within the PSD, and can access both CaMKII and AMPAR. This assumption causes the two forms of bistability to function in lockstep, where the activation of the AMPAR switch causes the CaMKII switch to turn on. This occurs because the two switches share the same pool of PP1 enzymes, and phosphatase saturation resulting from the AMPAR switch activates CaMKII. It is likely that the biological situation involves different degrees of PP1 mobility between multiple possible synaptic targets, and may even differ for the same synapse in different contexts. In our study, we obtain distinct outcomes for two cases of PP1 mobility and targeting. This sensitivity of synaptic state to PP1 mobility is a testable prediction and highlights the possible importance of subtle details of PSD anchoring on synaptic function.

An alternative proposal for multiple levels of synaptic activation is that the CaMKII–NMDAR complex may act in a highly modular manner (reviewed by Lisman and McIntyre [3]). In this scenario, each CaMKII–NMDAR complex can independently persist in a high or low state of activity. This situation would make it possible for an individual synapse to present graded levels of conductance depending on the number of active CaMKII complexes. Our model of CaMKII is at the bulk rather than holoenzyme level, and is too coarse-grained to address this possibility. The CaMKII phosphorylation of AMPAR plays two roles in our study. First, it directly increases the conductance of individual receptor tetramers. Second, it indirectly leads to an increase in the number of receptors at the synapse, by producing additional phosphorylation states of AMPAR for PP1 to act on. In the model, this leads to further saturation of the phosphatase and ultimately to an increase in surface AMPAR. There is recent evidence that CaMKII activity may also affect AMPAR numbers [37]. This would provide another mechanism for coupling between our proposed bistable mechanisms.

In addition to forming multiple synaptic states, our simulations show that coexisting bistable mechanisms may function to “hand-over” information about synaptic state from one switch to another. For example, in model 5 (weakly coupled synaptic switches), a rather brief Ca^2+^ input is sufficient to activate CaMKII, which can then turn on the AMPAR switch over a timescale of hours (Figure 9B). This is loosely analogous to different forms of computer information storage, where information is initially stored in fast but volatile form (e.g., RAM) and is later transferred to slow but stable forms of memory (e.g., hard disk).

Our study illustrates how two mechanisms for synaptic bistability may coexist to give rise to multiple possible synaptic states. We propose that the synapse may exhibit a combinatorial set of states through the interactions of several molecular switches. These may include local protein synthesis feedback loops involving mTOR, self-assembly processes at the synapse, or presynaptic switches. From the cell-biological perspective, we have considered synaptic recruitment mechanisms for only two of the hundreds of postsynaptic molecules. All these molecules undergo turnover, and many experience regulated movement similar to that in the switches we have analysed. We suggest that there are many forms of self-recruitment, coordinated self-assembly, and other potentially switch-like processes that contribute to the maintenance of different constituents and states of the synapse.

## Materials and Methods

### Model development.

Our model was developed to closely tie with experimental observations and to build on existing, well-documented, and experimentally constrained models. Two molecular trafficking cycles form the core of the model: (1) the trafficking of AMPARs, and in particular GluR12, between internal vesicular pools and the synaptic membrane associated with the PSD and (2) the movement of CaMKII to and from the spine cytosol and the PSD (see Figure 1). As elaborated below, we developed the models using published experimental observations on these trafficking processes, and considerable specific data on the biochemistry of the phosphorylation of these molecules. A few regulatory pathways were also modelled to provide signalling input and context. Reactions in the model take place in two compartments: the PSD and the bulk cytosolic volume of the spine. The receptors are membrane-associated, so the PSD-associated synaptic membrane is included in the PSD compartment. Likewise, the internalised, vesicular pool of receptors is included in the cytosolic compartment. The PSD volume is taken as 0.01 fl, and the spine head volume as 0.09 fl. There is a third, dendritic compartment of 5 fl that is occupied only by diffusible cAMP and by a bulk AMPAR pool. The bulk AMPAR pool is assumed to be at a steady level and is meant to represent synthesis and degradation of the receptor. No reactions occur in the dendritic compartment as it is meant only to couple diffusively with the spine.

### AMPAR model.

Due to a combinatorial proliferation of states, the reaction diagram of the AMPAR steps appears complex. As described below, we modelled 16 phosphorylation states of the receptor each in the internal and synaptic-membrane-associated pools. However, most of the reactions involving these 16 states were symmetric as they involved independent phosphorylation sites. This simplifies the model definition. We assumed that symmetric reactions had the same rates, so our model relies on only a few trafficking and phosphorylation parameters. AMPARs occur as tetrameric structures [44] with most AMPARs composed of two subunits each of GluR1 and GluR2 (GluR12) or GluR2 and GluR3 (GluR23). GluR23 receptors show constitutive trafficking and are responsible for basal synaptic transmission whereas GluR12 receptor insertion can be altered by stimuli [10,20]. We considered only GluR12 receptors in the model to focus on activity-stimulated events. The dynamics of GluR12 AMPAR trafficking were determined by kinase/phosphatase activities at the Ser845 sites of the two GluR1 subunits in the tetrameric GluR12 complex. Dephosphorylation of these sites by phosphatases triggers endocytosis whereas phosphorylation by PKA is required for synaptic membrane targeting [10,11]. We modelled the phosphorylation/dephosphorylation as a two-step reaction, where phosphorylation or dephosphorylation of both GluR1 subunits is necessary for synaptic membrane targeting or internalisation, respectively. Through simulations we found that basal activities of PP1 and PKA can account for the constitutive cycling of receptors in our model, consistent with experimental studies [8,45]. We assumed that PKA and PP1 were the relevant enzymes, but the model does not exclude the possibility that the same cycling effects might be mediated by other phosphorylation enzymes.

There is evidence that the membrane-associated PP1 dephosphorylates AMPARs only in the PSD, as loading neurons with active PP1 does not alter basal synaptic strength transmission [46]. Hence, we assumed that PP1 acts on GluR1 only in the PSD whereas PKA phosphorylates GluR1 in both compartments. In both compartments, Ser845 of GluR1 was also dephosphorylated by PP2B [10,47,48], which itself is inactive at basal Ca^2+^ concentrations.

Phosphorylation of Ser831 of the GluR1 subunit by CaMKII alters channel properties of the receptor in that the phosphorylation increases channel conductance approximately 2-fold [49]. As for the Ser845 sites, we modelled Ser831 phosphorylation of GluR1 so that both sites of a tetrameric complex could be phosphorylated individually by CaMKII. Dephosphorylation of Ser831 was modelled to occur only in the PSD, as internalisation was reported not to alter the phosphorylation state of AMPARs at Ser831 [10].

In the bistable models we explicitly modelled protein turnover through activity-dependent degradation [1]. The activity dependence was introduced by restricting the turnover to the doubly Ser845-phosphorylated states in the internal pool of AMPARs (see Figure 5A). There are around 150 receptors in an active dendritic spine [50]. We represented this constraint in the model as an anchor protein (possibly GRIP [8]) required for AMPAR insertion into the synaptic membrane.

The synaptic AMPAR conductance is a function both of the number of synaptic membrane receptors, and of their phosphorylation state. We assumed that if a single GluR1 subunit was phosphorylated on the CaMKII site (Ser831), the channel conductance was 1.5 times the basal level, and if two GluR1 subunits were phosphorylated the channel conductance doubled. In the figures, conductances are expressed as percent maximal conductance. We obtained the maximal conductance by considering that all the anchor protein was occupied, and that all the AMPARs were doubly phosphorylated and hence had double the basal conductance.

### CaMKII model.

The CaMKII model was derived closely from a previously developed single-compartment model of CaMKII activity [4,51]. This model is duplicated for the cytosol and the PSD and trafficking steps included. There is evidence that PP2A dephosphorylates CaMKII in the cytosol and PP1 in the PSD [52–54]. Because of limited data about the PP2A activity, we represented the cytosolic dephosphorylation step as involving a distinct phosphatase from the PP1 in the PSD, but using the same kinetics as PP1.

Binding of Ca^2+^/CaM is necessary and sufficient for the kinase to translocate to the PSD [55], where it binds to the NMDAR [56,57]. As we lacked direct association constants between CaMKII and NMDAR, we used time course information to constrain translocation of CaMKII to the PSD [14]. NMDAR was modelled as a putative binding site within the PSD [58]. Robust translocation away from the PSD occurs upon removal of the Ca^2+^ stimulus, and phosphorylation of Thr305 is required in this process [14]. However, only simultaneous dephosphorylation at Thr286 is sufficient for effective dissociation of CaMKII from the PSD [14,59].

### Other pathways.

There are numerous regulatory inputs, which are taken from a pre-existing library of signalling pathway models ([19]; the DOQCS database [http://doqcs.ncbs.res.in]). The parameters of these models are substantially the same, with the exception of PP2B (calcineurin), the cAMP pathway, and some scaling of phosphatase activities.

In the case of PP2B we did not vary any rates, but we eliminated the catalytic activity of two substates (Ca2.CaM.Ca4.CaN and Ca3.CaM.Ca4.CaN) as their contribution to the total was small (data not shown), and since the inclusion of these additional phosphorylation steps for all states of AMPAR would have substantially increased the number of reactions in the model.

In the case of cAMP we increased cyclase concentrations by a factor of approximately 4-fold, to get integral numbers of molecules in the model and to compensate for the reduction in assumed ATP concentrations from earlier model values of 5 mM to 2 mM. Phosphodiesterase concentrations have also been scaled up to maintain effective cAMP concentrations. A diffusion step is modelled for cAMP exchange with the dendrite, using a diffusion constant from frog olfactory neurons [60].

The phosphatase rates were scaled to obtain correct steady-state phosphorylation levels of inhibitor 1 of PP1 and CaMKII. PP1 rates and concentrations were also scaled, as described in the Results section, for the model of CaMKII bistability.

Most molecules in the simulation were modelled as independent pools for the PSD and cytosol. Only PKA was assumed to have access to both the cytosolic and PSD volumes. The adenylyl cyclase pathway was modelled only in the cytosolic volume. Because of its rapid diffusion, cAMP was modelled as exchanging between the spine head cytosol and the dendrite. We make an implicit assumption that the concentration of spine head constituents is maintained over the long periods of our simulations, through unspecified trafficking or other processes.

### Computations.

Simulations were performed on Linux workstations and on a Linux cluster (Atipa Technologies, Lawrence, Kansas, United States) for stochastic calculations. Models were implemented using Kinetikit/GENESIS [61], and solved using the Exponential Euler method [61]. Enzyme reactions were modelled with an explicit enzyme–substrate complex, with the exception of the adenylyl cyclase activity (see Figure 1G), which used the Michaelis-Menten form to improve numerical stability.

Stochastic calculations were done using an adaptive stochastic method [62] and using the Gillespie exact stochastic method as implemented in GENESIS 3/MOOSE [27]. The exact stochastic calculations used the Mersenne Twister random number generator [63]. When using the exact stochastic method, the entire model was simulated with the Gillespie method. Thus, all reactions led to integral changes in the numbers of the variable molecules. A few molecules in the model are buffered. The numbers of these buffered molecules were folded into the corresponding rate terms for efficiency. For example, if we have the reaction A <==> B and A is buffered, then the propensity of formation of B is d*n*_B_/dt = kf.*n*_A_ − kb.*n*_B_, where *n*_A_ and *n*_B_ are the numbers of molecules of A and B respectively. Since A is buffered, the value of *n*_A_ is fixed and we replaced this equation with d*n*_B_/dt = kf′ − kb.*n*_B_, where kf′ = kf.*n*_A._ This substitution also meant that it was possible to use nonintegral numbers for buffered molecules. This was meant to represent situations where the chemical buffering system on average gave rise to a nonintegral number of molecules.

### Stochastic transition time calculations.

Several lengthy stochastic runs were performed to estimate transition times between states of the models. In order to obtain longer samples, we set off many independent simulations in parallel on a cluster using distinct random number seeds, typically for a simulation time of 120,000 s (approximately 33 h) each. Transition times were estimated for a set of independent simulations as follows. Let *T* be the time to first transition in a given run, or total time of the run if there were no transitions. Let *N* be the number of runs where there were transitions. Then transition time for the entire sample is Σ*T*/*N*. In some cases there were no transitions at all, even for a large sample of runs. In these cases *N* was zero, so we could only set a lower bound to the transition time to be of the order of Σ*T*.

In some cases (e.g., Figure 7G) we estimated individual transition times by summing *T* for successive runs until the first run that had a transition. This sum gave the estimated transition time. Then the sum was reset to zero and the process repeated for the next transition.

### Estimation of thresholds (unstable fixed points).

Thresholds for transition between lower and upper states of AMPAR in the spine were estimated using an iterative bisection method. The range of possible values was known from the upper and lower steady states of the bistable model (model 1). These were set as the upper and lower limits *U* and *L,* respectively. The first estimate *E* of the threshold was halfway between *U* and *L: E* = (*U* + *L*)/2. The model was equilibrated at *E* receptors by blocking the AMPAR exchange with the bulk and AMPAR degradation. Then the exchange and degradation reactions were unblocked, and the model was run out for 10,000 s. Depending on whether *E* was above or below the actual threshold point, the model settled toward *U* or *L,* respectively. If *E* was high, then *U* was reassigned to *E*. If *E* was low, then *L* was reassigned to *E*. This process was repeated seven times to obtain an approximately 1% accurate estimate of the threshold.

### Model and simulator availability.

Complete model parameters and reaction schemes are presented in Protocol S1. All models, demonstration simulations, and the GENESIS/Kinetikit simulator are freely available at http://www.ncbs.res.in/~bhalla/AMPAR_switch/index.html. Models 0 to 5 (including the buffered PKA version of model 3) are deposited in the DOQCS database (http://doqcs.ncbs.res.in) as accession numbers 59 to 65.

## Supporting Information

Figure S1 Transient Stimuli and Responses of Model 0 with the Total Number of AMPARs Set to 80 (51 KB PDF)

Figure S2 Responses of Different Bistable Models to Transient Inputs (60 KB PDF)

Protocol S1 Model Equations and Parameters (240 KB PDF)

## Abbreviations

AMPA: alpha-amino-3-hydroxy-5-methyl-4-isoxazole propionate
AMPAR: alpha-amino-3-hydroxy-5-methyl-4-isoxazole propionate receptor
Aut-CaMKII: autonomously active CaMKII–PSD
CaM: calmodulin
CaMKII: calcium calmodulin type II kinase
cAMP: cyclic adenosine monophosphate
GluR12: glutamate receptor heteromer of subtypes 1 and 2
GluR23: glutamate receptor heteromer of subtypes 2 and 3
LTD: long-term depression
LTP: long-term potentiation
MAPK: mitogen-activated protein kinase
mTOR: mammalian target of rapamycin
NMDAR: N-methyl-D-aspartate receptor
PKA: protein kinase A
PP1: protein phosphatase 1
PP2B: protein phosphatase 2B
PSD: postsynaptic density
