# Supplementary material for: Molecular Switches at the Synapse Emerge from Receptor and Kinase Traffic
Source: PLoS Comput Biol. 2005 Jul 29;1(2):e20. doi: 10.1371/journal.pcbi.0010020 (PMC1185646; doi:10.1371/journal.pcbi.0010020)
Supplement: Figure S1 — (51 KB PDF) [file pcbi.0010020.sg001.pdf]

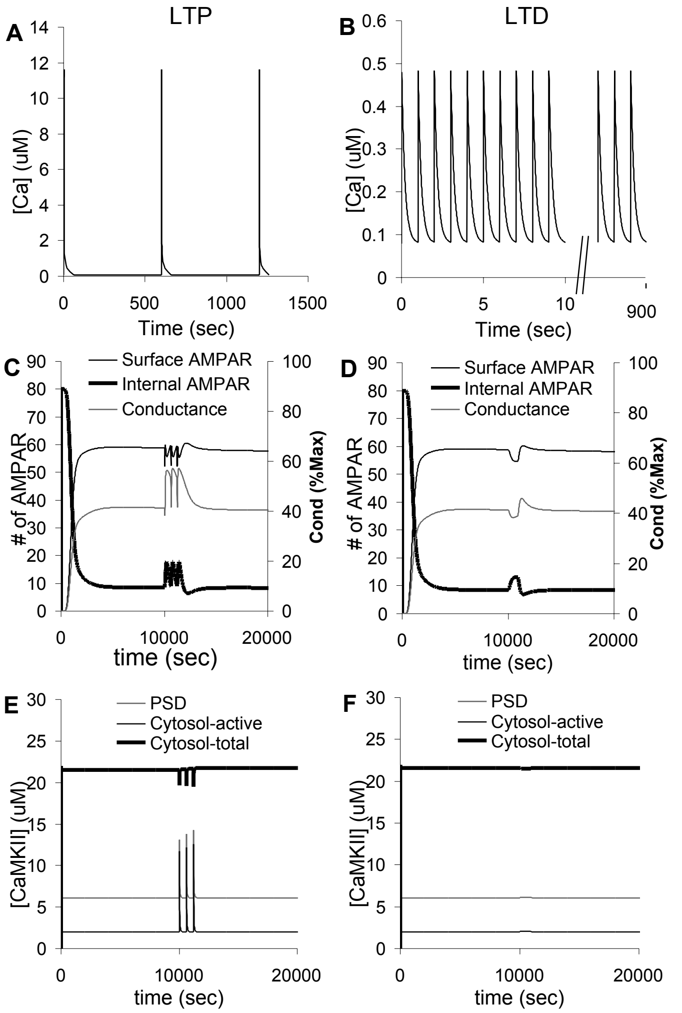

Supplementary Figure 1. Transient stimuli and responses of model 0 with the total number of AMPAR set to 80. A: Calcium waveform for LTP stimulus with 3 strong tetani of 100 Hz, 1 second, separated by 600 seconds. B. Calcium waveform for LTD stimulus with 900 pulses at 1 Hz. C. AMPAR responses to LTP stimulus. Levels of receptor in the internal and membrane pools are shown. Conductance is computed as a percentage of the value if all the AMPAR were in the membrane and doubly phosphorylated. Phosphorylation of a single GluR1-Ser831 is assumed to give 1.5x unphosphorylated conductance, and phosphorylation of both GluR1-Ser831 subunits gives 2x the unphosphorylated conductance. D. AMPAR responses to LTD stimulus. E. CaMKII responses to LTP stimulus, showing movement of kinase between cytosolic and PSD fractions. F. CaMKII responses to LTD stimulus.
