# Supplementary material for: Molecular Switches at the Synapse Emerge from Receptor and Kinase Traffic
Source: PLoS Comput Biol. 2005 Jul 29;1(2):e20. doi: 10.1371/journal.pcbi.0010020 (PMC1185646; doi:10.1371/journal.pcbi.0010020)
Supplement: Figure S2 — (60 KB PDF) [file pcbi.0010020.sg002.pdf]

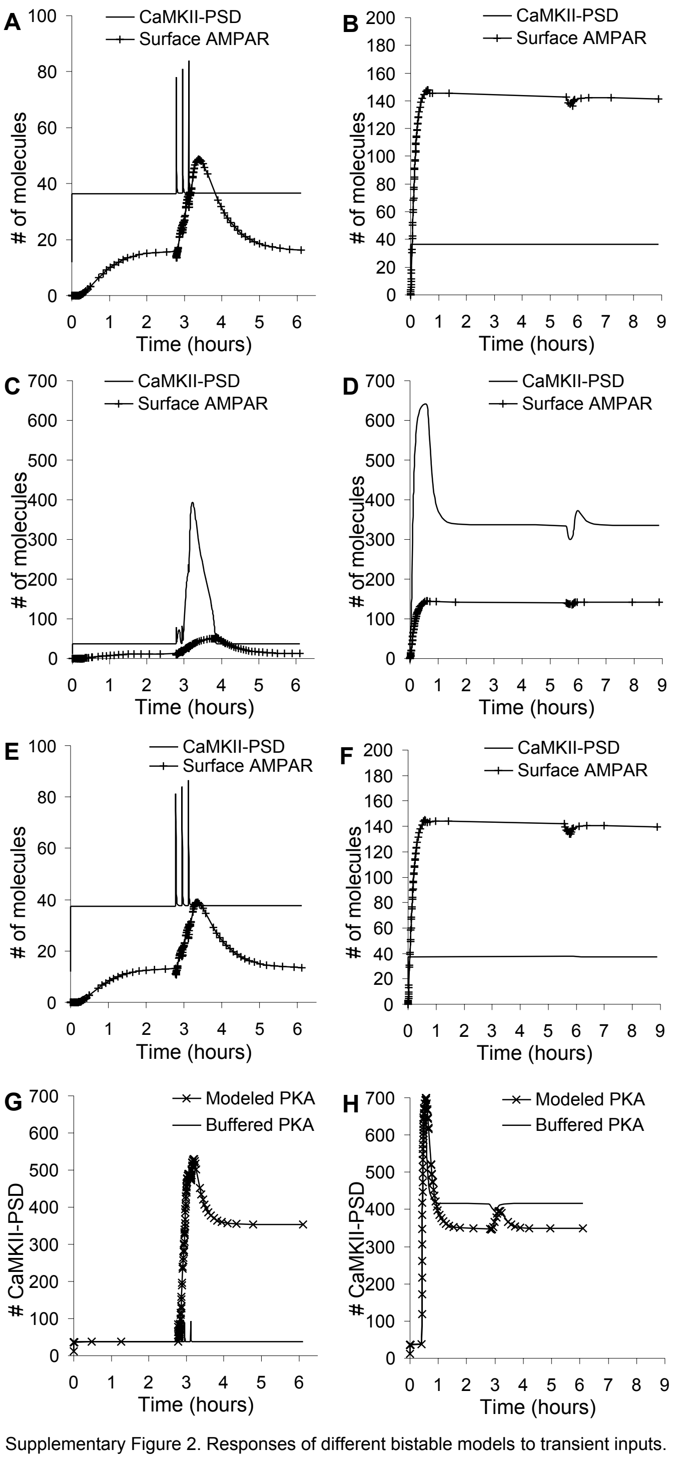

Supplementary Figure 2. Responses of different bistable models to transient inputs. Left column (A,C,E,G) received LTP input as in Supplementary Figure 1. Right column (B,D,F,H) received LTD input after having settled into active state following a 1 uM cAMP stimulus for 2000 sec at the start of the simulation. A, B: Model 1. Neither stimulus causes a state change. CaMKII is not bistable in this model, so it does not have a high state. C, D. Model 4. Neither stimulus causes a state change. E, F. Model 5. Neither stimulus causes a state change. G, H. CaMKII bistability (Model 3). LTP stimulus switches CaMKII on only for the case where the entire PKA pathway is modeled, including CaM-activated AC. The LTD stimulus failed to cause a state change in any of the models.
