# Supplementary material for: Molecular Switches at the Synapse Emerge from Receptor and Kinase Traffic
Source: PLoS Comput Biol. 2005 Jul 29;1(2):e20. doi: 10.1371/journal.pcbi.0010020 (PMC1185646; doi:10.1371/journal.pcbi.0010020)
Supplement: Protocol S1 — (240 KB PDF) [file pcbi.0010020.sd001.pdf]

A. Model Parameters for basic synaptic traffic model, Models 0 and Model 1. Almost all reactions are identical. Five reactions indicated in the AMPAR secton have zero rates in model 0.
Main pathways: AMPA receptor (AMPAR), Calcium Calmodulin type II Kinase (CaMKII), Calmodulin (CaM), Inhibitor 1 (I1), Protein phosphatase 2 B (PP2B), Adenylyl Cyclase (AC
Protein Kinase A (PKA).
Concentration units: uM (micromolar) for rate constants presented as Kf, Kb, Km
#/cell for rate constants presented as kf, kb, k1, k2, k3. This formulation of rates may depend on cellular volume.
Some reactions represent traffic between compartments of different volumes. In these cases, any concentration units should be used with care. The #/cell units are preferable in such cases as they are unambiguous.
Time units: Seconds in all cases.
Total Volume of Synapse = 0.1 femtoliters (fl)
Volume of cytosolic portion = 0.09 fl
Volume of Postsynaptic Density (PSD) = 0.01 fl
The enzyme rates are related as follows:
Km = (k2 + k3)/k1 (after conversion of units)
Kcat = k3.
Ratio = k2/k3

Initial concentrations (CoInit) are mostly zero, except for a few key molecules.
There is a flag for 'buffered' in the molecule concentration table. When this flag is zero the molecule concentrations are computed according to the reaction equations. If the flag is one the molecule concentration is held fixed to its initial concentration.

For clarity, the model is organized into 'groups' which roughly correspond to individual pathways. The '/kinetics' group is a set of shared molecules interacting with more than one pathway. The entire model scheme is then repeated as composite tables for molecules, reactions and enzymes.

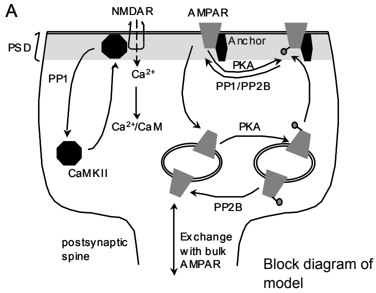

Block diagram of model

Concentration units: uM
Time units: sec
Default Volume (m^3) : 9e-20

Equations for group /kinetics
Reactions for group /kinetics

Reaction	kf	kb	Kf	Kb
PKC-control <===> PKC-active	2.5 s^-1	2.5 s^-1	2.5 s^-1	2.5 s^-1
Ca_control_cyt <===> Ca	100 s^-1	100 s^-1	100 s^-1	100 s^-1
Ca_control_PSD <===> Ca-PSD	100 s^-1	100 s^-1	100 s^-1	100 s^-1

Enzymes for group /kinetics

Enzyme-reaction	k1	k2	k3	Km	kcat	ratio
neurogranin ---PKC-active--> neurogranin*	0.0018889 #^-1.s^-1	2.34 s^-1	0.58 s^-1	28.627 uM	0.58 s^-1	4.0345
neurogranin-CaM ---PKC-active--> CaM + neurogranin*	0.0011333 #^-1.s^-1	1.4 s^-1	0.35 s^-1	28.596 uM	0.35 s^-1	4
AC2 ---PKC-active--> AC2*	0.011111 #^-1.s^-1	16 s^-1	4 s^-1	33.334 uM	4 s^-1	4
neurogranin_PSD ---PKC-active--> neurogranin*_PSD	0.0018889 #^-1.s^-1	2.34 s^-1	0.58 s^-1	28.627 uM	0.58 s^-1	4.0345
neurogranin-CaM_PSD ---PKC-active--> CaM-PSD + neurogranin*_PSD	0.0011333 #^-1.s^-1	1.4 s^-1	0.35 s^-1	28.596 uM	0.35 s^-1	4

Pools for group /kinetics

name	InitialConc	buffered	Volume
Ca	0.08 uM	0	0.09 fl
Ca-PSD	0.08 uM	0	0.01 fl
PKC-active	0.1 uM	0	0.09 fl
PKC-control	0.1 uM	1	0.09 fl
Ca_control_cyt	0.08 uM	1	0.09 fl
Ca_control_PSD	0.08 uM	1	0.01 fl

CaMKII reactions

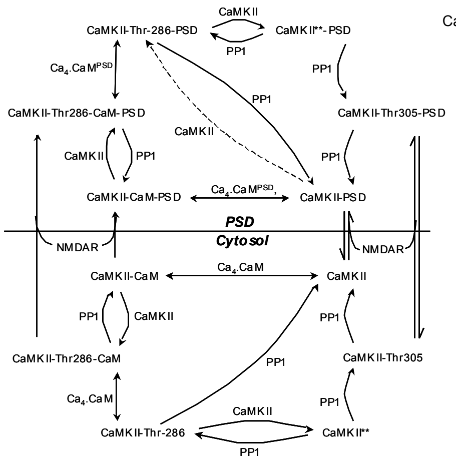

Reactions for group /kinetics/CaMKII

Reaction	kf	kb	Kf	Kb	
CaM-Ca4 + CaMKII <====> CaMKII-CaM	0.92592 #^-1.s^-1	5 s^-1	50 uM^-1.s^-1	5 s^-1	
CaMKII-thr286 + CaM-Ca4 <====> CaMKII-thr286*-CaM	18.522 #^-1.s^-1	0.1 s^-1	1000.2 uM^-1.s^-1	0.1 s^-1	
CaMKII-thr305-PSD <====> CaMK-thr305 + NMDAR	0.3 s^-1	1e-05 #^-1.s^-1	0.3 s^-1	6e-05 uM^-1.s^-1	Traffic Reacn
CaMKII-PSD <====> CaMKII + NMDAR	0.3 s^-1	1e-05 #^-1.s^-1	0.3 s^-1	6e-05 uM^-1.s^-1	Traffic Reacn
CaMKII-CaM + NMDAR <====> CaMKII-CaM-PSD	2e-05 #^-1.s^-1	0 s^-1	0.00108 uM^-1.s^-1	0 s^-1	Traffic Reacn
CaMKII-thr286*-CaM + NMDAR <====> CaMKII-thr286-CaM-PSD	2e-05 #^-1.s^-1	0 s^-1	0.00108 uM^-1.s^-1	0 s^-1	Traffic Reacn
basal_CaMKII_PSD_control <====> basal_CaMKII_PSD	1 s^-1	1 s^-1	1 s^-1	1 s^-1	
CaMKII-CaM-PSD <====> CaM-Ca4-PSD + CaMKII-PSD	5 s^-1	0 #^-1.s^-1	5 s^-1	0 uM^-1.s^-1	
CaMKII-PSD + CaM-Ca4-PSD <====> CaMKII-CaM-PSD	8.3333 #^-1.s^-1	0 s^-1	50 uM^-1.s^-1	0 s^-1	
CaMKII-thr286-PSD + CaM-Ca4-PSD <====> CaMKII-thr286-CaM-PSD	166.67 #^-1.s^-1	0.1 s^-1	1000 uM^-1.s^-1	0.1 s^-1	

Enzymes for group /kinetics/CaMKII

Enzyme-reaction	k1	k2	k3	Km	kcat	ratio
CaMKII-thr286 ---tot_CaM_CaMKII--> CaMKII***	0.0048904 #^-1.s^-1	24 s^-1	6 s^-1	113.6 uM	6 s^-1	4
CaMKII-CaM ---tot_CaM_CaMKII--> CaMKII-thr286*-CaM	0.00040753 #^-1.s^-1	2 s^-1	0.5 s^-1	113.6 uM	0.5 s^-1	4
CaMKII-thr286 ---tot_autonomous_CaMKII--> CaMKII***	0.0031746 #^-1.s^-1	24 s^-1	6 s^-1	175 uM	6 s^-1	4
CaMKII-CaM ---tot_autonomous_CaMKII--> CaMKII-thr286*-CaM	0.00026456 #^-1.s^-1	2 s^-1	0.5 s^-1	174.99 uM	0.5 s^-1	4
CaMKII-thr286-PSD ---tot-auto-PSD--> CaMKII***-PSD	0.04 #^-1.s^-1	24 s^-1	6 s^-1	125 uM	6 s^-1	4
CaMKII-CaM-PSD ---tot-auto-PSD--> CaMKII-thr286-CaM-PSD	0.0033333 #^-1.s^-1	2 s^-1	0.5 s^-1	125 uM	0.5 s^-1	4
A845*_B845* ---actCaMKII-PSD--> A831*845*_B845*	0.0046296 #^-1.s^-1	2 s^-1	0.5 s^-1	90.001 uM	0.5 s^-1	4
A_B845* ---actCaMKII-PSD--> A831*_B845*	0.0046296 #^-1.s^-1	2 s^-1	0.5 s^-1	90.001 uM	0.5 s^-1	4
A845*_B ---actCaMKII-PSD--> A831*845*_B	0.0046296 #^-1.s^-1	2 s^-1	0.5 s^-1	90.001 uM	0.5 s^-1	4
A_B ---actCaMKII-PSD--> A831*_B	0.0046296 #^-1.s^-1	2 s^-1	0.5 s^-1	90.001 uM	0.5 s^-1	4
A845*_B845* ---actCaMKII-PSD--> A845*_B831*845*	0.0046296 #^-1.s^-1	2 s^-1	0.5 s^-1	90.001 uM	0.5 s^-1	4
A_B845* ---actCaMKII-PSD--> A_B831*845*	0.0046296 #^-1.s^-1	2 s^-1	0.5 s^-1	90.001 uM	0.5 s^-1	4
A845*_B ---actCaMKII-PSD--> A845*_B831*	0.0046296 #^-1.s^-1	2 s^-1	0.5 s^-1	90.001 uM	0.5 s^-1	4
A_B ---actCaMKII-PSD--> A_B831*	0.0046296 #^-1.s^-1	2 s^-1	0.5 s^-1	90.001 uM	0.5 s^-1	4
A845*_B831*845* ---actCaMKII-PSD--> A835*845*_B835*845*	0.0046296 #^-1.s^-1	2 s^-1	0.5 s^-1	90.001 uM	0.5 s^-1	4
A_B831*845* ---actCaMKII-PSD--> A831*_B831*845*	0.0046296 #^-1.s^-1	2 s^-1	0.5 s^-1	90.001 uM	0.5 s^-1	4
A845*_B831* ---actCaMKII-PSD--> A831*845*_B831*	0.0046296 #^-1.s^-1	2 s^-1	0.5 s^-1	90.001 uM	0.5 s^-1	4
A_B831* ---actCaMKII-PSD--> A831*_B831*	0.0046296 #^-1.s^-1	2 s^-1	0.5 s^-1	90.001 uM	0.5 s^-1	4
A831*845*_B845* ---actCaMKII-PSD--> A835*845*_B835*845*	0.0046296 #^-1.s^-1	2 s^-1	0.5 s^-1	90.001 uM	0.5 s^-1	4
A831*_B845* ---actCaMKII-PSD--> A831*_B831*845*	0.0046296 #^-1.s^-1	2 s^-1	0.5 s^-1	90.001 uM	0.5 s^-1	4
A831*845*_B ---actCaMKII-PSD--> A831*845*_B831*	0.0046296 #^-1.s^-1	2 s^-1	0.5 s^-1	90.001 uM	0.5 s^-1	4
A831*_B ---actCaMKII-PSD--> A831*_B831*	0.0046296 #^-1.s^-1	2 s^-1	0.5 s^-1	90.001 uM	0.5 s^-1	4
CaMKII-thr286-PSD ---tot-CaM-CaMKII-PSD--> CaMKII***-PSD	0.061599 #^-1.s^-1	24 s^-1	6 s^-1	81.17 uM	6 s^-1	4
CaMKII-CaM-PSD ---tot-CaM-CaMKII-PSD--> CaMKII-thr286-CaM-PSD	0.0051333 #^-1.s^-1	2 s^-1	0.5 s^-1	81.169 uM	0.5 s^-1	4

Pools for group /kinetics/CaMKII

name	InitialConc	buffered	Volume
CaMKII	20 uM	0	0.09 fl
CaMKII-CaM	0 uM	0	0.09 fl
CaMKII-thr286*-CaM	0 uM	0	0.09 fl
CaMKII***	0 uM	0	0.09 fl
CaMKII-thr286	0 uM	0	0.09 fl
tot_CaM_CaMKII	0 uM	0	0.09 fl
tot_autonomous_CaMKII	2 uM	0	0.09 fl
CaMK-thr305	0 uM	0	0.09 fl
tot_CaMKII_cyt	22 uM	0	0.09 fl
act_CaMKII_cyt	2 uM	0	0.09 fl
basal_CaMKII_cyt	2 uM	1	0.09 fl
basal_CaMKII_PSD_control	2 uM	1	0.01 fl
CaMKII-thr305-PSD	0 uM	0	0.01 fl
CaMKII***-PSD	0 uM	0	0.01 fl
CaMKII-PSD	0 uM	0	0.01 fl
NMDAR	120 uM	0	0.01 fl
CaMKII-thr286-PSD	0 uM	0	0.01 fl
CaMKII-CaM-PSD	0 uM	0	0.01 fl
CaMKII-thr286-CaM-PSD	0 uM	0	0.01 fl
tot-auto-PSD	2 uM	0	0.01 fl
basal_CaMKII_PSD	2 uM	0	0.01 fl
tot_CaMKII_PSD	2 uM	0	0.01 fl
actCaMKII-PSD	2 uM	0	0.01 fl
tot-CaM-CaMKII-PSD	0 uM	0	0.01 fl
286P-PSD	0 uM	0	0.01 fl

CaM reactions

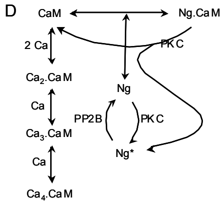

Equations for group /kinetics/CaM
Reactions for group /kinetics/CaM

Reaction	kf	kb	Kf	Kb	
CaM + 2 Ca <===> CaM-TR2-Ca2	0.024691 #^-2.s^-1	72 s^-1	71.999 uM^-2.s^-1	72 s^-1	
CaM-TR2-Ca2 + Ca <===> CaM-Ca3	0.066667 #^-1.s^-1	10 s^-1	3.6 uM^-1.s^-1	10 s^-1	
neurogranin + CaM <===> neurogranin-CaM	0.0055556 #^-1.s^-1	1 s^-1	0.3 uM^-1.s^-1	1 s^-1	
neurogranin* <===> neurogranin	0.005 s^-1	0 s^-1	0.005 s^-1	0 s^-1	
CaM-PSD + 2 Ca-PSD <===> CaM-TR2-Ca2-PSD	2 #^-2.s^-1	72 s^-1	72 uM^-2.s^-1	72 s^-1	
CaM-TR2-Ca2-PSD + Ca-PSD <===> CaM-Ca3-PSD	0.6 #^-1.s^-1	10 s^-1	3.6 uM^-1.s^-1	10 s^-1	
CaM-Ca3-PSD + Ca-PSD <===> CaM-Ca4-PSD	0.077502 #^-1.s^-1	10 s^-1	0.46501 uM^-1.s^-1	10 s^-1	
neurogranin_PSD + CaM-PSD <===> neurogranin-CaM_PSD	0.05 #^-1.s^-1	1 s^-1	0.3 uM^-1.s^-1	1 s^-1	
neurogranin*_PSD <===> neurogranin_PSD	0.005 s^-1	0 s^-1	0.005 s^-1	0 s^-1	
CaM-Ca3 + Ca <===> CaM-Ca4	0.0086111 #^-1.s^-1	10 s^-1	0.465 uM^-1.s^-1	10 s^-1	
CaM-Ca4-PSD <===> CaM-Ca4	540 s^-1	60 s^-1	540 s^-1	60 s^-1	Traffic Reacn.

Pools for group /kinetics/CaM

name	InitialConc	buffered	Volume
CaM	26.333 uM	0	0.09 fl
neurogranin-CaM	0 uM	0	0.09 fl
neurogranin*	0 uM	0	0.09 fl
neurogranin	10 uM	0	0.09 fl
CaM-PSD	26.333 uM	0	0.01 fl
neurogranin-CaM_PSD	0 uM	0	0.01 fl
neurogranin_PSD	10 uM	0	0.01 fl
neurogranin*_PSD	0 uM	0	0.01 fl
CaM-TR2-Ca2	0 uM	0	0.09 fl
CaM-Ca3	0 uM	0	0.09 fl
CaM-Ca4-PSD	0 uM	0	0.01 fl
CaM-Ca3-PSD	0 uM	0	0.01 fl
CaM-TR2-Ca2-PSD	0 uM	0	0.01 fl
CaM-Ca4	0 uM	0	0.09 fl

PP1 reactions

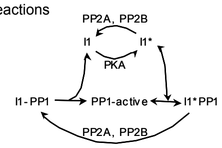

Equations for group /kinetics/PP1

Reactions for group /kinetics/PP1

Reaction	kf	kb	Kf	Kb
I1* + PP1-active <===> PP1-I1*	9.2589 #^-1.s^-1	0.1 s^-1	499.98 uM^-1.s^-1	0.1 s^-1
PP1-I1 <===> PP1-active + I1	1 s^-1	0 #^-1.s^-1	1 s^-1	0 uM^-1.s^-1

Enzymes for group /kinetics/PP1

Enzyme-reaction	k1	k2	k3	Km	kcat	ratio
I1* ---PP2A--> I1	0.01196 #^-1.s^-1	8.3334 s^-1	2 s^-1	16 uM	2 s^-1	4.1667
PP1-I1* ---PP2A--> PP1-I1	0.01196 #^-1.s^-1	8.3334 s^-1	2 s^-1	16 uM	2 s^-1	4.1667
I1* ---PP2A--> I1	0.01196 #^-1.s^-1	8.3334 s^-1	2 s^-1	16 uM	2 s^-1	4.1667
PP1-I1* ---PP2A--> PP1-I1	0.01196 #^-1.s^-1	8.3334 s^-1	2 s^-1	16 uM	2 s^-1	4.1667
CaMKII-thr286*-CaM ---PP1-active--> CaMKII-CaM	0.045397 #^-1.s^-1	10 s^-1	2.5 s^-1	5.099 uM	2.5 s^-1	4
CaMKII-thr286 ---PP1-active--> CaMKII	0.045397 #^-1.s^-1	10 s^-1	2.5 s^-1	5.099 uM	2.5 s^-1	4
CaMKII*** ---PP1-active--> CaMKII-thr286	0.045397 #^-1.s^-1	10 s^-1	2.5 s^-1	5.099 uM	2.5 s^-1	4
CaMKII*** ---PP1-active--> CaMK-thr305	0.045397 #^-1.s^-1	10 s^-1	2.5 s^-1	5.099 uM	2.5 s^-1	4
CaMK-thr305 ---PP1-active--> CaMKII	0.045397 #^-1.s^-1	10 s^-1	2.5 s^-1	5.099 uM	2.5 s^-1	4

Pools for group /kinetics/PP1

name	InitialConc	buffered	Volume
I1	1.8 uM	0	0.09 fl
I1*	0 uM	0	0.09 fl
PP1-I1*	0 uM	0	0.09 fl
PP1-I1	0 uM	0	0.09 fl
PP2A	0.11111 uM	0	0.09 fl
PP1-active	1.8 uM	0	0.09 fl

Equations for group /kinetics/PP1_PSD

Reactions for group /kinetics/PP1_PSD

Reaction	kf	kb	Kf	Kb
PP1-I1 <===> I1 + PP1-active_PSD	1 s^-1	0 #^-1.s^-1	1 s^-1	0 uM^-1.s^-1
I1* + PP1-active_PSD <===> PP1-I1*	83.33 #^-1.s^-1	0.1 s^-1	499.98 uM^-1.s^-1	0.1 s^-1

Enzymes for group /kinetics/PP1_PSD

Enzyme-reaction	k1	k2	k3	Km	kcat	ratio
CaMKII-thr286-CaM-PSD ---PP1-active_PSD--> CaMKII-CaM-PSD	1.0417 #^-1.s^-1	10 s^-1	2.5 s^-1	1.9999 uM	2.5 s^-1	4
CaMKII-thr286-PSD ---PP1-active_PSD--> CaMKII-PSD	1.0417 #^-1.s^-1	10 s^-1	2.5 s^-1	1.9999 uM	2.5 s^-1	4
CaMKII***-PSD ---PP1-active_PSD--> CaMKII-thr286-PSD	0.40857 #^-1.s^-1	10 s^-1	2.5 s^-1	5.0991 uM	2.5 s^-1	4
CaMKII***-PSD ---PP1-active_PSD--> CaMKII-thr305-PSD	1.0417 #^-1.s^-1	10 s^-1	2.5 s^-1	1.9999 uM	2.5 s^-1	4
A845*_B845* ---PP1-active_PSD--> A_B845*	0.14583 #^-1.s^-1	0.68 s^-1	0.17 s^-1	0.97145 uM	0.17 s^-1	4
A845*_B845* ---PP1-active_PSD--> A845*_B	0.14583 #^-1.s^-1	0.68 s^-1	0.17 s^-1	0.97145 uM	0.17 s^-1	4
A845*_B ---PP1-active_PSD--> A_B	0.14583 #^-1.s^-1	0.68 s^-1	0.17 s^-1	0.97145 uM	0.17 s^-1	4
A_B845* ---PP1-active_PSD--> A_B	0.14583 #^-1.s^-1	0.68 s^-1	0.17 s^-1	0.97145 uM	0.17 s^-1	4
A831*845*_B845* ---PP1-active_PSD--> A831*_B845*	0.14583 #^-1.s^-1	0.68 s^-1	0.17 s^-1	0.97145 uM	0.17 s^-1	4
A831*845*_B845* ---PP1-active_PSD--> A831*845*_B	0.14583 #^-1.s^-1	0.68 s^-1	0.17 s^-1	0.97145 uM	0.17 s^-1	4
A831*_B845* ---PP1-active_PSD--> A831*_B	0.14583 #^-1.s^-1	0.68 s^-1	0.17 s^-1	0.97145 uM	0.17 s^-1	4
A831*845*_B ---PP1-active_PSD--> A831*_B	0.14583 #^-1.s^-1	0.68 s^-1	0.17 s^-1	0.97145 uM	0.17 s^-1	4
A831*845*_B845* ---PP1-active_PSD--> A845*_B845*	0.14583 #^-1.s^-1	1.4 s^-1	0.35 s^-1	2 uM	0.35 s^-1	4
A831*_B845* ---PP1-active_PSD--> A_B845*	0.14583 #^-1.s^-1	1.4 s^-1	0.35 s^-1	2 uM	0.35 s^-1	4
A831*845*_B ---PP1-active_PSD--> A845*_B	0.14583 #^-1.s^-1	1.4 s^-1	0.35 s^-1	2 uM	0.35 s^-1	4
A831*_B ---PP1-active_PSD--> A_B	0.14583 #^-1.s^-1	1.4 s^-1	0.35 s^-1	2 uM	0.35 s^-1	4
A845*_B831*845* ---PP1-active_PSD--> A_B831*845*	0.14583 #^-1.s^-1	0.68 s^-1	0.17 s^-1	0.97145 uM	0.17 s^-1	4
A845*_B831*845* ---PP1-active_PSD--> A845*_B831*	0.14583 #^-1.s^-1	0.68 s^-1	0.17 s^-1	0.97145 uM	0.17 s^-1	4
A_B831*845* ---PP1-active_PSD--> A_B831*	0.14583 #^-1.s^-1	0.68 s^-1	0.17 s^-1	0.97145 uM	0.17 s^-1	4
A845*_B831* ---PP1-active_PSD--> A_B831*	0.14583 #^-1.s^-1	0.68 s^-1	0.17 s^-1	0.97145 uM	0.17 s^-1	4
A835*845*_B835*845* ---PP1-active_PSD--> A831*_B831*845*	0.14583 #^-1.s^-1	0.68 s^-1	0.17 s^-1	0.97145 uM	0.17 s^-1	4
A835*845*_B835*845* ---PP1-active_PSD--> A831*845*_B831*	0.14583 #^-1.s^-1	0.68 s^-1	0.17 s^-1	0.97145 uM	0.17 s^-1	4
A831*_B831*845* ---PP1-active_PSD--> A831*_B831*	0.14583 #^-1.s^-1	0.68 s^-1	0.17 s^-1	0.97145 uM	0.17 s^-1	4
A831*845*_B831* ---PP1-active_PSD--> A831*_B831*	0.14583 #^-1.s^-1	0.68 s^-1	0.17 s^-1	0.97145 uM	0.17 s^-1	4
A_B831*845* ---PP1-active_PSD--> A_B845*	0.14583 #^-1.s^-1	1.4 s^-1	0.35 s^-1	2 uM	0.35 s^-1	4
A845*_B831*845* ---PP1-active_PSD--> A845*_B845*	0.14583 #^-1.s^-1	1.4 s^-1	0.35 s^-1	2 uM	0.35 s^-1	4
A845*_B831* ---PP1-active_PSD--> A845*_B	0.14583 #^-1.s^-1	1.4 s^-1	0.35 s^-1	2 uM	0.35 s^-1	4
A_B831* ---PP1-active_PSD--> A_B	0.14583 #^-1.s^-1	1.4 s^-1	0.35 s^-1	2 uM	0.35 s^-1	4
A835*845*_B835*845* ---PP1-active_PSD--> A845*_B831*845*	0.14583 #^-1.s^-1	1.4 s^-1	0.35 s^-1	2 uM	0.35 s^-1	4
A831*_B831*845* ---PP1-active_PSD--> A_B831*845*	0.14583 #^-1.s^-1	1.4 s^-1	0.35 s^-1	2 uM	0.35 s^-1	4
A831*845*_B831* ---PP1-active_PSD--> A845*_B831*	0.14583 #^-1.s^-1	1.4 s^-1	0.35 s^-1	2 uM	0.35 s^-1	4
A831*_B831* ---PP1-active_PSD--> A_B831*	0.14583 #^-1.s^-1	1.4 s^-1	0.35 s^-1	2 uM	0.35 s^-1	4
A835*845*_B835*845* ---PP1-active_PSD--> A831*845*_B845*	0.14583 #^-1.s^-1	1.4 s^-1	0.35 s^-1	2 uM	0.35 s^-1	4
A831*_B831*845* ---PP1-active_PSD--> A831*_B845*	0.14583 #^-1.s^-1	1.4 s^-1	0.35 s^-1	2 uM	0.35 s^-1	4
A831*845*_B831* ---PP1-active_PSD--> A831*845*_B	0.14583 #^-1.s^-1	1.4 s^-1	0.35 s^-1	2 uM	0.35 s^-1	4
A831*_B831* ---PP1-active_PSD--> A831*_B	0.14583 #^-1.s^-1	1.4 s^-1	0.35 s^-1	2 uM	0.35 s^-1	4
CaMKII-thr305-PSD ---PP1-active_PSD--> CaMKII-PSD	0.40857 #^-1.s^-1	10 s^-1	2.5 s^-1	5.0991 uM	2.5 s^-1	4

Pools for group /kinetics/PP1_PSD

name	InitialConc	buffered	Volume
I1	4 uM	0	0.01 fl
I1*	0 uM	0	0.01 fl
PP1-I1*	0 uM	0	0.01 fl
PP1-I1	0 uM	0	0.01 fl
PP1-active_PSD	4 uM	0	0.01 fl

PP2B/Calcineurin
reactions

F

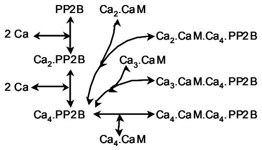

Equations for group /kinetics/PP2B
Reactions for group /kinetics/PP2B

Reaction	kf	kb	Kf	Kb
2 Ca + CaNAB-Ca2 <===> CaNAB-Ca4	0.0012346 #^-2.s^-1	1 s^-1	3.6001 uM^-2.s^-1	1 s^-1
CaNAB + 2 Ca <===> CaNAB-Ca2	3.4321 #^-2.s^-1	1 s^-1	10008 uM^-2.s^-1	1 s^-1
CaM-Ca4 + CaNAB-Ca4 <===> CaM_Ca_n-CaNAB	11.111 #^-1.s^-1	1 s^-1	599.99 uM^-1.s^-1	1 s^-1

Enzymes for group /kinetics/PP2B

Enzyme-reaction	k1	k2	k3	Km	kcat	ratio
I1* ---CaNAB-Ca4--> I1	0.00063333 #^-1.s^-1	0.136 s^-1	0.034 s^-1	4.9708 uM	0.034 s^-1	4
I1* ---CaNAB-Ca4--> I1	0.00063334 #^-1.s^-1	0.136 s^-1	0.034 s^-1	4.9707 uM	0.034 s^-1	4
neurogranin* ---CaM_Ca_n-CaNAB--> neurogranin	0.0061778 #^-1.s^-1	2.67 s^-1	0.67 s^-1	10.012 uM	0.67 s^-1	3.9851
I1* ---CaM_Ca_n-CaNAB--> I1	0.0063333 #^-1.s^-1	1.36 s^-1	0.34 s^-1	4.9708 uM	0.34 s^-1	4
PP1-I1* ---CaM_Ca_n-CaNAB--> PP1-I1	0.0063333 #^-1.s^-1	1.36 s^-1	0.34 s^-1	4.9708 uM	0.34 s^-1	4
I1* ---CaM_Ca_n-CaNAB--> I1	0.0063334 #^-1.s^-1	1.36 s^-1	0.34 s^-1	4.9707 uM	0.34 s^-1	4
PP1-I1* ---CaM_Ca_n-CaNAB--> PP1-I1	0.0063334 #^-1.s^-1	1.36 s^-1	0.34 s^-1	4.9707 uM	0.34 s^-1	4
A845*_B ---CaM_Ca_n-CaNAB--> A_B	0.037256 #^-1.s^-1	8 s^-1	2 s^-1	4.9706 uM	2 s^-1	4
A831*845*_B ---CaM_Ca_n-CaNAB--> A831*_B	0.037256 #^-1.s^-1	8 s^-1	2 s^-1	4.9706 uM	2 s^-1	4
A845*_B831* ---CaM_Ca_n-CaNAB--> A_B831*	0.037256 #^-1.s^-1	8 s^-1	2 s^-1	4.9706 uM	2 s^-1	4
A831*845*_B831* ---CaM_Ca_n-CaNAB--> A831*_B831*	0.037256 #^-1.s^-1	8 s^-1	2 s^-1	4.9706 uM	2 s^-1	4
A_B845* ---CaM_Ca_n-CaNAB--> A_B	0.037256 #^-1.s^-1	8 s^-1	2 s^-1	4.9706 uM	2 s^-1	4
AA831*_B845* ---CaM_Ca_n-CaNAB--> A831*_B	0.037256 #^-1.s^-1	8 s^-1	2 s^-1	4.9706 uM	2 s^-1	4
A_B831*845* ---CaM_Ca_n-CaNAB--> A_B831*	0.037256 #^-1.s^-1	8 s^-1	2 s^-1	4.9706 uM	2 s^-1	4
A831*_B831*845* ---CaM_Ca_n-CaNAB--> A831*_B831*	0.037256 #^-1.s^-1	8 s^-1	2 s^-1	4.9706 uM	2 s^-1	4
A845*_B845* ---CaM_Ca_n-CaNAB--> A_B845*	0.037256 #^-1.s^-1	8 s^-1	2 s^-1	4.9706 uM	2 s^-1	4
A831*845*_B845* ---CaM_Ca_n-CaNAB--> AA831*_B845*	0.037256 #^-1.s^-1	8 s^-1	2 s^-1	4.9706 uM	2 s^-1	4
A845*_B831*845* ---CaM_Ca_n-CaNAB--> A_B831*845*	0.037256 #^-1.s^-1	8 s^-1	2 s^-1	4.9706 uM	2 s^-1	4
A835*845*_B835*845* ---CaM_Ca_n-CaNAB--> A831*_B831*845*	0.037256 #^-1.s^-1	8 s^-1	2 s^-1	4.9706 uM	2 s^-1	4
A845*_B845* ---CaM_Ca_n-CaNAB--> A845*_B	0.037256 #^-1.s^-1	8 s^-1	2 s^-1	4.9706 uM	2 s^-1	4
A831*845*_B845* ---CaM_Ca_n-CaNAB--> A831*845*_B	0.037256 #^-1.s^-1	8 s^-1	2 s^-1	4.9706 uM	2 s^-1	4
A845*_B831*845* ---CaM_Ca_n-CaNAB--> A845*_B831*	0.037256 #^-1.s^-1	8 s^-1	2 s^-1	4.9706 uM	2 s^-1	4
A835*845*_B835*845* ---CaM_Ca_n-CaNAB--> A831*845*_B831*	0.037256 #^-1.s^-1	8 s^-1	2 s^-1	4.9706 uM	2 s^-1	4
A845*_B845* ---CaM_Ca_n-CaNAB--> A_B845*	0.037256 #^-1.s^-1	8 s^-1	2 s^-1	4.9706 uM	2 s^-1	4
A845*_B845* ---CaM_Ca_n-CaNAB--> A845*_B	0.037256 #^-1.s^-1	8 s^-1	2 s^-1	4.9706 uM	2 s^-1	4
A_B845* ---CaM_Ca_n-CaNAB--> A_B	0.037256 #^-1.s^-1	8 s^-1	2 s^-1	4.9706 uM	2 s^-1	4
A845*_B ---CaM_Ca_n-CaNAB--> A_B	0.037256 #^-1.s^-1	8 s^-1	2 s^-1	4.9706 uM	2 s^-1	4
A831*845*_B845* ---CaM_Ca_n-CaNAB--> A831*_B845*	0.037256 #^-1.s^-1	8 s^-1	2 s^-1	4.9706 uM	2 s^-1	4
A831*845*_B845* ---CaM_Ca_n-CaNAB--> A831*845*_B	0.037256 #^-1.s^-1	8 s^-1	2 s^-1	4.9706 uM	2 s^-1	4
A831*_B845* ---CaM_Ca_n-CaNAB--> A831*_B	0.037256 #^-1.s^-1	8 s^-1	2 s^-1	4.9706 uM	2 s^-1	4
A831*845*_B ---CaM_Ca_n-CaNAB--> A831*_B	0.037256 #^-1.s^-1	8 s^-1	2 s^-1	4.9706 uM	2 s^-1	4
A845*_B831*845* ---CaM_Ca_n-CaNAB--> A_B831*845*	0.037256 #^-1.s^-1	8 s^-1	2 s^-1	4.9706 uM	2 s^-1	4
A845*_B831*845* ---CaM_Ca_n-CaNAB--> A845*_B831*	0.037256 #^-1.s^-1	8 s^-1	2 s^-1	4.9706 uM	2 s^-1	4
A_B831*845* ---CaM_Ca_n-CaNAB--> A_B831*	0.037256 #^-1.s^-1	8 s^-1	2 s^-1	4.9706 uM	2 s^-1	4
A845*_B831* ---CaM_Ca_n-CaNAB--> A_B831*	0.037256 #^-1.s^-1	8 s^-1	2 s^-1	4.9706 uM	2 s^-1	4
A835*845*_B835*845* ---CaM_Ca_n-CaNAB--> A831*_B831*845*	0.037256 #^-1.s^-1	8 s^-1	2 s^-1	4.9706 uM	2 s^-1	4
A835*845*_B835*845* ---CaM_Ca_n-CaNAB--> A831*845*_B831*	0.037256 #^-1.s^-1	8 s^-1	2 s^-1	4.9706 uM	2 s^-1	4
A831*845*_B831* ---CaM_Ca_n-CaNAB--> A831*_B831*	0.037256 #^-1.s^-1	8 s^-1	2 s^-1	4.9706 uM	2 s^-1	4
A831*_B831*845* ---CaM_Ca_n-CaNAB--> A831*_B831*	0.037256 #^-1.s^-1	8 s^-1	2 s^-1	4.9706 uM	2 s^-1	4
neurogranin*_PSD ---CaM_Ca_n-CaNAB--> neurogranin_PSD	0.0061778 #^-1.s^-1	2.67 s^-1	0.67 s^-1	10.012 uM	0.67 s^-1	3.9851

Pools for group /kinetics/PP2B

name	InitialConc	buffered	Volume
CaNAB	1 uM	0	0.09 fl
CaNAB-Ca2	0 uM	0	0.09 fl
CaNAB-Ca4	0 uM	0	0.09 fl
CaM_Ca_n-CaNAB	0 uM	0	0.09 fl

PKA reactions

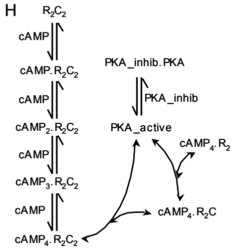

Equations for group /kinetics/PKA

Reactions for group /kinetics/PKA

Reaction	kf	kb	Kf	Kb
R2C2 + cAMP <===> R2C2-cAMP	1 #^-1.s^-1	33 s^-1	54 uM^-1.s^-1	33 s^-1
R2C2-cAMP + cAMP <===> R2C2-cAMP2	1 #^-1.s^-1	33 s^-1	54 uM^-1.s^-1	33 s^-1
R2C2-cAMP2 + cAMP <===> R2C2-cAMP3	1.3889 #^-1.s^-1	110 s^-1	75.001 uM^-1.s^-1	110 s^-1
cAMP + R2C2-cAMP3 <===> R2C2-cAMP4	1.3889 #^-1.s^-1	32.5 s^-1	75.001 uM^-1.s^-1	32.5 s^-1
R2C2-cAMP4 <===> PKA-active + R2C-cAMP4	60 s^-1	0.33333 #^-1.s^-1	60 s^-1	18 uM^-1.s^-1
R2C-cAMP4 <===> PKA-active + R2-cAMP4	60 s^-1	0.33333 #^-1.s^-1	60 s^-1	18 uM^-1.s^-1
PKA-active + PKA-inhibitor <===> inhibited-PKA	1.1111 #^-1.s^-1	1 s^-1	59.999 uM^-1.s^-1	1 s^-1

Enzymes for group /kinetics/PKA

Enzyme-reaction	k1	k2	k3	Km	kcat	ratio
cAMP-PDE ---PKA-active--> cAMP-PDE*	0.11111 #^-1.s^-1	36 s^-1	9 s^-1	7.5001 uM	9 s^-1	4
I1 ---PKA-active--> I1*	0.11111 #^-1.s^-1	36 s^-1	9 s^-1	7.5001 uM	9 s^-1	4
I1 ---PKA-active--> I1*	0.11111 #^-1.s^-1	36 s^-1	9 s^-1	7.5001 uM	9 s^-1	4
A_B ---PKA-active--> A845*_B	0.074072 #^-1.s^-1	24 s^-1	6 s^-1	7.5002 uM	6 s^-1	4
A831*_B ---PKA-active--> A831*845*_B	0.074072 #^-1.s^-1	24 s^-1	6 s^-1	7.5002 uM	6 s^-1	4
A_B831* ---PKA-active--> A845*_B831*	0.074072 #^-1.s^-1	24 s^-1	6 s^-1	7.5002 uM	6 s^-1	4
A831*_B831* ---PKA-active--> A831*845*_B831*	0.074072 #^-1.s^-1	24 s^-1	6 s^-1	7.5002 uM	6 s^-1	4
A_B ---PKA-active--> A_B845*	0.074072 #^-1.s^-1	24 s^-1	6 s^-1	7.5002 uM	6 s^-1	4
A831*_B ---PKA-active--> AA831*_B845*	0.074072 #^-1.s^-1	24 s^-1	6 s^-1	7.5002 uM	6 s^-1	4
A_B831* ---PKA-active--> A_B831*845*	0.074072 #^-1.s^-1	24 s^-1	6 s^-1	7.5002 uM	6 s^-1	4
A831*_B831* ---PKA-active--> A831*_B831*845*	0.074072 #^-1.s^-1	24 s^-1	6 s^-1	7.5002 uM	6 s^-1	4
A_B845* ---PKA-active--> A845*_B845*	0.074072 #^-1.s^-1	24 s^-1	6 s^-1	7.5002 uM	6 s^-1	4
AA831*_B845* ---PKA-active--> A831*845*_B845*	0.074072 #^-1.s^-1	24 s^-1	6 s^-1	7.5002 uM	6 s^-1	4
A_B831*845* ---PKA-active--> A845*_B831*845*	0.074072 #^-1.s^-1	24 s^-1	6 s^-1	7.5002 uM	6 s^-1	4
A831*_B831*845* ---PKA-active--> A835*845*_B835*845*	0.074072 #^-1.s^-1	24 s^-1	6 s^-1	7.5002 uM	6 s^-1	4
A845*_B ---PKA-active--> A845*_B845*	0.074072 #^-1.s^-1	24 s^-1	6 s^-1	7.5002 uM	6 s^-1	4
A831*845*_B ---PKA-active--> A831*845*_B845*	0.074072 #^-1.s^-1	24 s^-1	6 s^-1	7.5002 uM	6 s^-1	4
A845*_B831* ---PKA-active--> A845*_B831*845*	0.074072 #^-1.s^-1	24 s^-1	6 s^-1	7.5002 uM	6 s^-1	4
A831*845*_B831* ---PKA-active--> A835*845*_B835*845*	0.074072 #^-1.s^-1	24 s^-1	6 s^-1	7.5002 uM	6 s^-1	4
A_B845* ---PKA-active--> A845*_B845*	0.074072 #^-1.s^-1	24 s^-1	6 s^-1	7.5002 uM	6 s^-1	4
A845*_B ---PKA-active--> A845*_B845*	0.074072 #^-1.s^-1	24 s^-1	6 s^-1	7.5002 uM	6 s^-1	4
A_B ---PKA-active--> A845*_B	0.074072 #^-1.s^-1	24 s^-1	6 s^-1	7.5002 uM	6 s^-1	4
A_B ---PKA-active--> A_B845*	0.074072 #^-1.s^-1	24 s^-1	6 s^-1	7.5002 uM	6 s^-1	4
A831*_B845* ---PKA-active--> A831*845*_B845*	0.074072 #^-1.s^-1	24 s^-1	6 s^-1	7.5002 uM	6 s^-1	4
A831*845*_B ---PKA-active--> A831*845*_B845*	0.074072 #^-1.s^-1	24 s^-1	6 s^-1	7.5002 uM	6 s^-1	4
A831*_B ---PKA-active--> A831*_B845*	0.074072 #^-1.s^-1	24 s^-1	6 s^-1	7.5002 uM	6 s^-1	4
A831*_B ---PKA-active--> A831*845*_B	0.074072 #^-1.s^-1	24 s^-1	6 s^-1	7.5002 uM	6 s^-1	4
A_B831*845* ---PKA-active--> A845*_B831*845*	0.074072 #^-1.s^-1	24 s^-1	6 s^-1	7.5002 uM	6 s^-1	4
A845*_B831* ---PKA-active--> A845*_B831*845*	0.074072 #^-1.s^-1	24 s^-1	6 s^-1	7.5002 uM	6 s^-1	4
A_B831* ---PKA-active--> A_B831*845*	0.074072 #^-1.s^-1	24 s^-1	6 s^-1	7.5002 uM	6 s^-1	4
A_B831* ---PKA-active--> A845*_B831*	0.074072 #^-1.s^-1	24 s^-1	6 s^-1	7.5002 uM	6 s^-1	4
A831*_B831*845* ---PKA-active--> A835*845*_B835*845*	0.074072 #^-1.s^-1	24 s^-1	6 s^-1	7.5002 uM	6 s^-1	4
A831*845*_B831* ---PKA-active--> A835*845*_B835*845*	0.074072 #^-1.s^-1	24 s^-1	6 s^-1	7.5002 uM	6 s^-1	4
A831*_B831* ---PKA-active--> A831*_B831*845*	0.074072 #^-1.s^-1	24 s^-1	6 s^-1	7.5002 uM	6 s^-1	4
A831*_B831* ---PKA-active--> A831*845*_B831*	0.074072 #^-1.s^-1	24 s^-1	6 s^-1	7.5002 uM	6 s^-1	4

Pools for group /kinetics/PKA

name	InitialConc	buffered	Volume
R2C2	0.5 uM	0	0.09 fl
R2C2-cAMP	0 uM	0	0.09 fl
R2C2-cAMP2	0 uM	0	0.09 fl
R2C2-cAMP3	0 uM	0	0.09 fl
R2C2-cAMP4	0 uM	0	0.09 fl
R2C-cAMP4	0 uM	0	0.09 fl
R2-cAMP4	0 uM	0	0.09 fl
PKA-inhibitor	0.25926 uM	0	0.09 fl
inhibited-PKA	0 uM	0	0.09 fl
PKA-active	0 uM	0	0.09 fl

AC/cAMP reactions

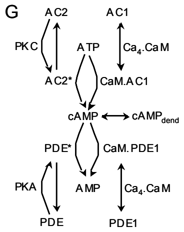

Equations for group /kinetics/AC

Reactions for group /kinetics/AC

Reaction	kf	kb	Kf	Kb
CaM-Ca4 + AC1 <===> AC1-CaM	0.92592 #^-1.s^-1	1 s^-1	50 uM^-1.s^-1	1 s^-1
AC2* <===> AC2	0.1 s^-1	0 s^-1	0.1 s^-1	0 s^-1
cAMP-PDE* <===> cAMP-PDE	0.01 s^-1	0 s^-1	0.01 s^-1	0 s^-1
PDE1 + CaM-Ca4 <===> CaM.PDE1	13.333 #^-1.s^-1	5 s^-1	719.98 uM^-1.s^-1	5 s^-1
cAMP <===> cAMP_in_dend	300 s^-1	5.4 s^-1	300 s^-1	5.4 s^-1

Enzymes for group /kinetics/AC

Enzyme-reaction	k1	k2	k3	Km	kcat	ratio
ATP ---AC1-CaM--> cAMP	0.0013889 #^-1.s^-1	18 s^-1	4.5 s^-1	300 uM	4.5 s^-1	4
ATP ---AC2*--> cAMP	0.00061728 #^-1.s^-1	8 s^-1	2 s^-1	300 uM	2 s^-1	4
cAMP ---cAMP-PDE--> AMP	0.046667 #^-1.s^-1	40 s^-1	10 s^-1	19.841 uM	10 s^-1	4
cAMP ---cAMP-PDE*--> AMP	0.093333 #^-1.s^-1	80 s^-1	20 s^-1	19.841 uM	20 s^-1	4
cAMP ---PDE1--> AMP	0.0038889 #^-1.s^-1	6.67 s^-1	1.667 s^-1	39.7 uM	1.667 s^-1	4.0012
cAMP ---CaM.PDE1--> AMP	0.023333 #^-1.s^-1	40 s^-1	10 s^-1	39.683 uM	10 s^-1	4

Pools for group /kinetics/AC

name	InitialConc	buffered	Volume
ATP	2000 uM	1	0.09 fl
AC1-CaM	0 uM	0	0.09 fl
AC1	0.074074 uM	0	0.09 fl
AC2*	0 uM	0	0.09 fl
AC2	0.074074 uM	0	0.09 fl
AMP	0 uM	0	0.09 fl
cAMP-PDE	0.55556 uM	0	0.09 fl
cAMP-PDE*	0 uM	0	0.09 fl
PDE1	2.5926 uM	0	0.09 fl
CaM.PDE1	0 uM	0	0.09 fl
cAMP_in_dend	0 uM	0	5 fl
cAMP	0 uM	0	0.09 fl

AMPAR trafficking reactions

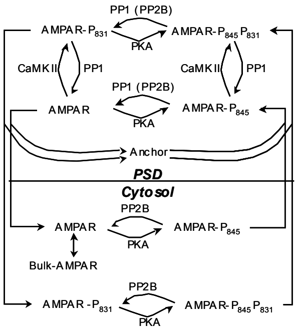

Equations for group /kinetics/AMPAR

Reactions for group /kinetics/AMPAR

Reaction		kf	kb	Kf	Kb	
A831*_B831* <===> A831*_B831* + Anchor		0.0008 s^-1	0 #^-1.s^-1	0.0008 s^-1	0 uM^-1.s^-1	Traffic Reacn
A_B831* <===> A_B831* + Anchor		0.0008 s^-1	0 #^-1.s^-1	0.0008 s^-1	0 uM^-1.s^-1	Traffic Reacn
A831*_B <===> A831*_B + Anchor		0.0008 s^-1	0 #^-1.s^-1	0.0008 s^-1	0 uM^-1.s^-1	Traffic Reacn
A_B <===> A_B + Anchor		0.0008 s^-1	0 #^-1.s^-1	0.0008 s^-1	0 uM^-1.s^-1	Traffic Reacn
A835*845*_B835*845* <===> AMPAR_deg	Model 1 only	3.6e-05 s^-1	0 s^-1	3.6e-05 s^-1	0 s^-1	
A845*_B831*845* <===> AMPAR_deg	Model 1 only	3.6e-05 s^-1	0 s^-1	3.6e-05 s^-1	0 s^-1	
A831*845*_B845* <===> AMPAR_deg	Model 1 only	3.6e-05 s^-1	0 s^-1	3.6e-05 s^-1	0 s^-1	
A845*_B845* <===> AMPAR_deg	Model 1 only	3.6e-05 s^-1	0 s^-1	3.6e-05 s^-1	0 s^-1	
A835*845*_B835*845* + Anchor <===> A835*845*_B835*845*		0.0002 #^-1.s^-1	0.008 s^-1	0.0108 uM^-1.s^-1	0.008 s^-1	Traffic Reacn
A845*_B831*845* + Anchor <===> A845*_B831*845*		0.0002 #^-1.s^-1	0.008 s^-1	0.0108 uM^-1.s^-1	0.008 s^-1	Traffic Reacn
A831*845*_B845* + Anchor <===> A831*845*_B845*		0.0002 #^-1.s^-1	0.008 s^-1	0.0108 uM^-1.s^-1	0.008 s^-1	Traffic Reacn
A845*_B845* + Anchor <===> A845*_B845*		0.0002 #^-1.s^-1	0.008 s^-1	0.0108 uM^-1.s^-1	0.008 s^-1	Traffic Reacn
AMPAR_bulk <===> A_B	Model 1 only	0.018 s^-1	1 s^-1	0.018 s^-1	1 s^-1	Traffic Reacn
GluR23_M <===> GluR23_I		0.00035 s^-1	0.0014 s^-1	0.00035 s^-1	0.0014 s^-1	Traffic Reacn

Pools for group /kinetics/AMPAR

name	InitialConc	buffered	Volume
GluR23_I	0.092593 uM	0	0.09 fl
AMPAR_deg	0 uM	1	0.09 fl
A_B	0 uM	0	0.09 fl
A831*_B	0 uM	0	0.09 fl
A845*_B	0 uM	0	0.09 fl
A831*845*_B	0 uM	0	0.09 fl
A_B845*	0 uM	0	0.09 fl
AA831*_B845*	0 uM	0	0.09 fl
A845*_B845*	0 uM	0	0.09 fl
A831*845*_B845*	0 uM	0	0.09 fl
A845*_B831*845*	0 uM	0	0.09 fl
A835*845*_B835*845*	0 uM	0	0.09 fl
A_B831*845*	0 uM	0	0.09 fl
A831*_B831*845*	0 uM	0	0.09 fl
A_B831*	0 uM	0	0.09 fl
A845*_B831*	0 uM	0	0.01 fl
A831*_B831*	0 uM	0	0.09 fl
A831*845*_B831*	0 uM	0	0.09 fl
AMPAR_bulk	0.011111 uM	1	5 fl
I_845	0 uM	0	0.09 fl
I_845-P	0 uM	0	0.09 fl
I_845_PP	0 uM	0	0.09 fl
tot_I_GluR12	0 uM	0	0.09 fl
total_Int	0.096296 uM	0	0.09 fl

Equations for group /kinetics/AMPAR_memb

Pools for group /kinetics/AMPAR_memb

name	InitialConc	buffered	Volume
A_B	0 uM	0	0.01 fl
A831*_B	0 uM	0	0.01 fl
A_B831*	0 uM	0	0.01 fl
A831*_B831*	0 uM	0	0.01 fl
A845*_B	0 uM	0	0.01 fl
A831*845*_B	0 uM	0	0.01 fl
A831*845*_B831*	0 uM	0	0.01 fl
A_B845*	0 uM	0	0.01 fl
A845*_B831*	0 uM	0	0.01 fl
A845*_B845*	0 uM	0	0.01 fl
A831*_B845*	0 uM	0	0.01 fl
A_B831*845*	0 uM	0	0.01 fl
A831*_B831*845*	0 uM	0	0.01 fl
A831*845*_B845*	0 uM	0	0.01 fl
A845*_B831*845*	0 uM	0	0.01 fl
A835*845*_B835*845*	0 uM	0	0.01 fl
GluR23_M	3.5 uM	0	0.01 fl

Ser845-PP	0 uM	0	0.01 fl
Ser845-P	0 uM	0	0.01 fl
Ser845	0 uM	0	0.01 fl
tot_mem_GluR12	0 uM	0	0.01 fl
total_mem	3.4667 uM	0	0.01 fl
Ser831	0 uM	0	0.01 fl
Ser831-P	0 uM	0	0.01 fl
Ser831-PP	0 uM	0	0.01 fl
Anchor	27.333 uM	0	0.01 fl

Same data, sorting by data type:

Equations for group /##[]

Reactions for group /##[]

Reaction	kf	kb	Kf	Kb
PKC-control <===> PKC-active	2.5 s^-1	2.5 s^-1	2.5 s^-1	2.5 s^-1
Ca_control_cyt <===> Ca	100 s^-1	100 s^-1	100 s^-1	100 s^-1
Ca_control_PSD <===> Ca-PSD	100 s^-1	100 s^-1	100 s^-1	100 s^-1
CaM-Ca4 + CaMKII <===> CaMKII-CaM	0.92592 #^-1.s^-1	5 s^-1	50 uM^-1.s^-1	5 s^-1
CaMKII-thr286 + CaM-Ca4 <===> CaMKII-thr286*-CaM	18.522 #^-1.s^-1	0.1 s^-1	1000.2 uM^-1.s^-1	0.1 s^-1
CaMKII-thr305-PSD <===> CaMK-thr305 + NMDAR	0.3 s^-1	1e-05 #^-1.s^-1	0.3 s^-1	6e-05 uM^-1.s^-1
CaMKII-PSD <===> CaMKII + NMDAR	0.3 s^-1	1e-05 #^-1.s^-1	0.3 s^-1	6e-05 uM^-1.s^-1
CaMKII-CaM + NMDAR <===> CaMKII-CaM-PSD	2e-05 #^-1.s^-1	0 s^-1	0.00108 uM^-1.s^-1	0 s^-1
CaMKII-thr286*-CaM + NMDAR <===> CaMKII-thr286-CaM-PSD	2e-05 #^-1.s^-1	0 s^-1	0.00108 uM^-1.s^-1	0 s^-1
basal_CaMKII_PSD_control <===> basal_CaMKII_PSD	1 s^-1	1 s^-1	1 s^-1	1 s^-1
CaMKII-CaM-PSD <===> CaM-Ca4-PSD + CaMKII-PSD	5 s^-1	0 #^-1.s^-1	5 s^-1	0 uM^-1.s^-1
CaMKII-PSD + CaM-Ca4-PSD <===> CaMKII-CaM-PSD	8.3333 #^-1.s^-1	0 s^-1	50 uM^-1.s^-1	0 s^-1
CaMKII-thr286-PSD + CaM-Ca4-PSD <===> CaMKII-thr286-CaM-PSD	166.67 #^-1.s^-1	0.1 s^-1	1000 uM^-1.s^-1	0.1 s^-1
CaM + 2 Ca <===> CaM-TR2-Ca2	0.024691 #^-2.s^-1	72 s^-1	71.999 uM^-2.s^-1	72 s^-1
CaM-TR2-Ca2 + Ca <===> CaM-Ca3	0.066667 #^-1.s^-1	10 s^-1	3.6 uM^-1.s^-1	10 s^-1
neurogranin + CaM <===> neurogranin-CaM	0.0055556 #^-1.s^-1	1 s^-1	0.3 uM^-1.s^-1	1 s^-1
neurogranin* <===> neurogranin	0.005 s^-1	0 s^-1	0.005 s^-1	0 s^-1
CaM-PSD + 2 Ca-PSD <===> CaM-TR2-Ca2-PSD	2 #^-2.s^-1	72 s^-1	72 uM^-2.s^-1	72 s^-1
CaM-TR2-Ca2-PSD + Ca-PSD <===> CaM-Ca3-PSD	0.6 #^-1.s^-1	10 s^-1	3.6 uM^-1.s^-1	10 s^-1
CaM-Ca3-PSD + Ca-PSD <===> CaM-Ca4-PSD	0.077502 #^-1.s^-1	10 s^-1	0.46501 uM^-1.s^-1	10 s^-1
neurogranin_PSD + CaM-PSD <===> neurogranin-CaM_PSD	0.05 #^-1.s^-1	1 s^-1	0.3 uM^-1.s^-1	1 s^-1
neurogranin*_PSD <===> neurogranin_PSD	0.005 s^-1	0 s^-1	0.005 s^-1	0 s^-1
CaM-Ca3 + Ca <===> CaM-Ca4	0.0086111 #^-1.s^-1	10 s^-1	0.465 uM^-1.s^-1	10 s^-1
CaM-Ca4-PSD <===> CaM-Ca4	540 s^-1	60 s^-1	540 s^-1	60 s^-1
I1* + PP1-active <===> PP1-I1*	9.2589 #^-1.s^-1	0.1 s^-1	499.98 uM^-1.s^-1	0.1 s^-1
PP1-I1 <===> PP1-active + I1	1 s^-1	0 #^-1.s^-1	1 s^-1	0 uM^-1.s^-1
2 Ca + CaNAB-Ca2 <===> CaNAB-Ca4	0.0012346 #^-2.s^-1	1 s^-1	3.6001 uM^-2.s^-1	1 s^-1
CaNAB + 2 Ca <===> CaNAB-Ca2	3.4321 #^-2.s^-1	1 s^-1	10008 uM^-2.s^-1	1 s^-1
CaM-Ca4 + CaNAB-Ca4 <===> CaM_Ca_n-CaNAB	11.111 #^-1.s^-1	1 s^-1	599.99 uM^-1.s^-1	1 s^-1
R2C2 + cAMP <===> R2C2-cAMP	1 #^-1.s^-1	33 s^-1	54 uM^-1.s^-1	33 s^-1
R2C2-cAMP + cAMP <===> R2C2-cAMP2	1 #^-1.s^-1	33 s^-1	54 uM^-1.s^-1	33 s^-1
R2C2-cAMP2 + cAMP <===> R2C2-cAMP3	1.3889 #^-1.s^-1	110 s^-1	75.001 uM^-1.s^-1	110 s^-1
cAMP + R2C2-cAMP3 <===> R2C2-cAMP4	1.3889 #^-1.s^-1	32.5 s^-1	75.001 uM^-1.s^-1	32.5 s^-1
R2C2-cAMP4 <===> PKA-active + R2C-cAMP4	60 s^-1	0.33333 #^-1.s^-1	60 s^-1	18 uM^-1.s^-1
R2C-cAMP4 <===> PKA-active + R2-cAMP4	60 s^-1	0.33333 #^-1.s^-1	60 s^-1	18 uM^-1.s^-1
PKA-active + PKA-inhibitor <===> inhibited-PKA	1.1111 #^-1.s^-1	1 s^-1	59.999 uM^-1.s^-1	1 s^-1
CaM-Ca4 + AC1 <===> AC1-CaM	0.92592 #^-1.s^-1	1 s^-1	50 uM^-1.s^-1	1 s^-1
AC2* <===> AC2	0.1 s^-1	0 s^-1	0.1 s^-1	0 s^-1
cAMP-PDE* <===> cAMP-PDE	0.01 s^-1	0 s^-1	0.01 s^-1	0 s^-1
PDE1 + CaM-Ca4 <===> CaM.PDE1	13.333 #^-1.s^-1	5 s^-1	719.98 uM^-1.s^-1	5 s^-1
cAMP <===> cAMP_in_dend	300 s^-1	5.4 s^-1	300 s^-1	5.4 s^-1
PP1-I1 <===> I1 + PP1-active_PSD	1 s^-1	0 #^-1.s^-1	1 s^-1	0 uM^-1.s^-1
I1* + PP1-active_PSD <===> PP1-I1*	83.33 #^-1.s^-1	0.1 s^-1	499.98 uM^-1.s^-1	0.1 s^-1
A831*_B831* <===> A831*_B831* + Anchor	0.0008 s^-1	0 #^-1.s^-1	0.0008 s^-1	0 uM^-1.s^-1
A_B831* <===> A_B831* + Anchor	0.0008 s^-1	0 #^-1.s^-1	0.0008 s^-1	0 uM^-1.s^-1
A831*_B <===> A831*_B + Anchor	0.0008 s^-1	0 #^-1.s^-1	0.0008 s^-1	0 uM^-1.s^-1
A_B <===> A_B + Anchor	0.0008 s^-1	0 #^-1.s^-1	0.0008 s^-1	0 uM^-1.s^-1
A835*845*_B835*845* <===> AMPAR_deg	3.6e-05 s^-1	0 s^-1	3.6e-05 s^-1	0 s^-1
A845*_B831*845* <===> AMPAR_deg	3.6e-05 s^-1	0 s^-1	3.6e-05 s^-1	0 s^-1
A831*845*_B845* <===> AMPAR_deg	3.6e-05 s^-1	0 s^-1	3.6e-05 s^-1	0 s^-1
A845*_B845* <===> AMPAR_deg	3.6e-05 s^-1	0 s^-1	3.6e-05 s^-1	0 s^-1
A835*845*_B835*845* + Anchor <===> A835*845*_B835*845*	0.0002 #^-1.s^-1	0.008 s^-1	0.0108 uM^-1.s^-1	0.008 s^-1
A845*_B831*845* + Anchor <===> A845*_B831*845*	0.0002 #^-1.s^-1	0.008 s^-1	0.0108 uM^-1.s^-1	0.008 s^-1
A831*845*_B845* + Anchor <===> A831*845*_B845*	0.0002 #^-1.s^-1	0.008 s^-1	0.0108 uM^-1.s^-1	0.008 s^-1
A845*_B845* + Anchor <===> A845*_B845*	0.0002 #^-1.s^-1	0.008 s^-1	0.0108 uM^-1.s^-1	0.008 s^-1
AMPAR_bulk <===> A_B	0.018 s^-1	1 s^-1	0.018 s^-1	1 s^-1
GluR23_M <===> GluR23_I	0.00035 s^-1	0.0014 s^-1	0.00035 s^-1	0.0014 s^-1

Enzymes for group /##[]

Enzyme-reaction	k1	k2	k3	Km	kcat	ratio
neurogranin ---PKC-active--> neurogranin*	0.0018889 #^-1.s^-1	2.34 s^-1	0.58 s^-1	28.627 uM	0.58 s^-1	4.0345
neurogranin-CaM ---PKC-active--> CaM + neurogranin*	0.0011333 #^-1.s^-1	1.4 s^-1	0.35 s^-1	28.596 uM	0.35 s^-1	4
AC2 ---PKC-active--> AC2*	0.011111 #^-1.s^-1	16 s^-1	4 s^-1	33.334 uM	4 s^-1	4
neurogranin_PSD ---PKC-active--> neurogranin*_PSD	0.0018889 #^-1.s^-1	2.34 s^-1	0.58 s^-1	28.627 uM	0.58 s^-1	4.0345
neurogranin-CaM_PSD ---PKC-active--> CaM-PSD + neurogranin*_PSD	0.0011333 #^-1.s^-1	1.4 s^-1	0.35 s^-1	28.596 uM	0.35 s^-1	4
CaMKII-thr286 ---tot_CaM_CaMKII--> CaMKII***	0.0048904 #^-1.s^-1	24 s^-1	6 s^-1	113.6 uM	6 s^-1	4
CaMKII-CaM ---tot_CaM_CaMKII--> CaMKII-thr286*-CaM	0.00040753 #^-1.s^-1	2 s^-1	0.5 s^-1	113.6 uM	0.5 s^-1	4
CaMKII-thr286 ---tot_autonomous_CaMKII--> CaMKII***	0.0031746 #^-1.s^-1	24 s^-1	6 s^-1	175 uM	6 s^-1	4
CaMKII-CaM ---tot_autonomous_CaMKII--> CaMKII-thr286*-CaM	0.00026456 #^-1.s^-1	2 s^-1	0.5 s^-1	174.99 uM	0.5 s^-1	4
CaMKII-thr286-PSD ---tot-auto-PSD--> CaMKII***-PSD	0.04 #^-1.s^-1	24 s^-1	6 s^-1	125 uM	6 s^-1	4
CaMKII-CaM-PSD ---tot-auto-PSD--> CaMKII-thr286-CaM-PSD	0.0033333 #^-1.s^-1	2 s^-1	0.5 s^-1	125 uM	0.5 s^-1	4
A845*_B845* ---actCaMKII-PSD--> A831*845*_B845*	0.0046296 #^-1.s^-1	2 s^-1	0.5 s^-1	90.001 uM	0.5 s^-1	4
A_B845* ---actCaMKII-PSD--> A831*_B845*	0.0046296 #^-1.s^-1	2 s^-1	0.5 s^-1	90.001 uM	0.5 s^-1	4
A845*_B ---actCaMKII-PSD--> A831*845*_B	0.0046296 #^-1.s^-1	2 s^-1	0.5 s^-1	90.001 uM	0.5 s^-1	4
A_B ---actCaMKII-PSD--> A831*_B	0.0046296 #^-1.s^-1	2 s^-1	0.5 s^-1	90.001 uM	0.5 s^-1	4
A845*_B845* ---actCaMKII-PSD--> A845*_B831*845*	0.0046296 #^-1.s^-1	2 s^-1	0.5 s^-1	90.001 uM	0.5 s^-1	4
A_B845* ---actCaMKII-PSD--> A_B831*845*	0.0046296 #^-1.s^-1	2 s^-1	0.5 s^-1	90.001 uM	0.5 s^-1	4
A845*_B ---actCaMKII-PSD--> A845*_B831*	0.0046296 #^-1.s^-1	2 s^-1	0.5 s^-1	90.001 uM	0.5 s^-1	4
A_B ---actCaMKII-PSD--> A_B831*	0.0046296 #^-1.s^-1	2 s^-1	0.5 s^-1	90.001 uM	0.5 s^-1	4
A845*_B831*845* ---actCaMKII-PSD--> A835*845*_B835*845*	0.0046296 #^-1.s^-1	2 s^-1	0.5 s^-1	90.001 uM	0.5 s^-1	4
A_B831*845* ---actCaMKII-PSD--> A831*_B831*845*	0.0046296 #^-1.s^-1	2 s^-1	0.5 s^-1	90.001 uM	0.5 s^-1	4
A845*_B831* ---actCaMKII-PSD--> A831*845*_B831*	0.0046296 #^-1.s^-1	2 s^-1	0.5 s^-1	90.001 uM	0.5 s^-1	4

A_B831* ---actCaMKII-PSD--> A831*_B831*	0.0046296 #^-1.s^-1	2 s^-1	0.5 s^-1	90.001 uM	0.5 s^-1	4
A831*845*_B845* ---actCaMKII-PSD--> A835*845*_B835*845*	0.0046296 #^-1.s^-1	2 s^-1	0.5 s^-1	90.001 uM	0.5 s^-1	4
A831*_B845* ---actCaMKII-PSD--> A831*_B831*845*	0.0046296 #^-1.s^-1	2 s^-1	0.5 s^-1	90.001 uM	0.5 s^-1	4
A831*845*_B ---actCaMKII-PSD--> A831*845*_B831*	0.0046296 #^-1.s^-1	2 s^-1	0.5 s^-1	90.001 uM	0.5 s^-1	4
A831*_B ---actCaMKII-PSD--> A831*_B831*	0.0046296 #^-1.s^-1	2 s^-1	0.5 s^-1	90.001 uM	0.5 s^-1	4
CaMKII-thr286-PSD ---tot-CaM-CaMKII-PSD--> CaMKII***-PSD	0.061599 #^-1.s^-1	24 s^-1	6 s^-1	81.17 uM	6 s^-1	4
CaMKII-CaM-PSD ---tot-CaM-CaMKII-PSD--> CaMKII-thr286-CaM-PSD	0.0051333 #^-1.s^-1	2 s^-1	0.5 s^-1	81.169 uM	0.5 s^-1	4
I1* ---PP2A--> I1	0.01196 #^-1.s^-1	8.3334 s^-1	2 s^-1	16 uM	2 s^-1	4.1667
PP1-I1* ---PP2A--> PP1-I1	0.01196 #^-1.s^-1	8.3334 s^-1	2 s^-1	16 uM	2 s^-1	4.1667
I1* ---PP2A--> I1	0.01196 #^-1.s^-1	8.3334 s^-1	2 s^-1	16 uM	2 s^-1	4.1667
PP1-I1* ---PP2A--> PP1-I1	0.01196 #^-1.s^-1	8.3334 s^-1	2 s^-1	16 uM	2 s^-1	4.1667
CaMKII-thr286*-CaM ---PP1-active--> CaMKII-CaM	0.045397 #^-1.s^-1	10 s^-1	2.5 s^-1	5.099 uM	2.5 s^-1	4
CaMKII-thr286 ---PP1-active--> CaMKII	0.045397 #^-1.s^-1	10 s^-1	2.5 s^-1	5.099 uM	2.5 s^-1	4
CaMKII*** ---PP1-active--> CaMKII-thr286	0.045397 #^-1.s^-1	10 s^-1	2.5 s^-1	5.099 uM	2.5 s^-1	4
CaMKII*** ---PP1-active--> CaMK-thr305	0.045397 #^-1.s^-1	10 s^-1	2.5 s^-1	5.099 uM	2.5 s^-1	4
CaMK-thr305 ---PP1-active--> CaMKII	0.045397 #^-1.s^-1	10 s^-1	2.5 s^-1	5.099 uM	2.5 s^-1	4
I1* ---CaNAB-Ca4--> I1	0.00063333 #^-1.s^-1	0.136 s^-1	0.034 s^-1	4.9708 uM	0.034 s^-1	4
I1* ---CaNAB-Ca4--> I1	0.00063334 #^-1.s^-1	0.136 s^-1	0.034 s^-1	4.9707 uM	0.034 s^-1	4
neurogranin* ---CaM_Ca_n-CaNAB--> neurogranin	0.0061778 #^-1.s^-1	2.67 s^-1	0.67 s^-1	10.012 uM	0.67 s^-1	3.9851
I1* ---CaM_Ca_n-CaNAB--> I1	0.0063333 #^-1.s^-1	1.36 s^-1	0.34 s^-1	4.9708 uM	0.34 s^-1	4
PP1-I1* ---CaM_Ca_n-CaNAB--> PP1-I1	0.0063333 #^-1.s^-1	1.36 s^-1	0.34 s^-1	4.9708 uM	0.34 s^-1	4
I1* ---CaM_Ca_n-CaNAB--> I1	0.0063334 #^-1.s^-1	1.36 s^-1	0.34 s^-1	4.9707 uM	0.34 s^-1	4
PP1-I1* ---CaM_Ca_n-CaNAB--> PP1-I1	0.0063334 #^-1.s^-1	1.36 s^-1	0.34 s^-1	4.9707 uM	0.34 s^-1	4
A845*_B ---CaM_Ca_n-CaNAB--> A_B	0.037256 #^-1.s^-1	8 s^-1	2 s^-1	4.9706 uM	2 s^-1	4
A831*845*_B ---CaM_Ca_n-CaNAB--> A831*_B	0.037256 #^-1.s^-1	8 s^-1	2 s^-1	4.9706 uM	2 s^-1	4
A845*_B831* ---CaM_Ca_n-CaNAB--> A_B831*	0.037256 #^-1.s^-1	8 s^-1	2 s^-1	4.9706 uM	2 s^-1	4
A831*845*_B831* ---CaM_Ca_n-CaNAB--> A831*_B831*	0.037256 #^-1.s^-1	8 s^-1	2 s^-1	4.9706 uM	2 s^-1	4
A_B845* ---CaM_Ca_n-CaNAB--> A_B	0.037256 #^-1.s^-1	8 s^-1	2 s^-1	4.9706 uM	2 s^-1	4
AA831*_B845* ---CaM_Ca_n-CaNAB--> A831*_B	0.037256 #^-1.s^-1	8 s^-1	2 s^-1	4.9706 uM	2 s^-1	4
A_B831*845* ---CaM_Ca_n-CaNAB--> A_B831*	0.037256 #^-1.s^-1	8 s^-1	2 s^-1	4.9706 uM	2 s^-1	4
A831*_B831*845* ---CaM_Ca_n-CaNAB--> A831*_B831*	0.037256 #^-1.s^-1	8 s^-1	2 s^-1	4.9706 uM	2 s^-1	4
A845*_B845* ---CaM_Ca_n-CaNAB--> A_B845*	0.037256 #^-1.s^-1	8 s^-1	2 s^-1	4.9706 uM	2 s^-1	4
A831*845*_B845* ---CaM_Ca_n-CaNAB--> AA831*_B845*	0.037256 #^-1.s^-1	8 s^-1	2 s^-1	4.9706 uM	2 s^-1	4
A845*_B831*845* ---CaM_Ca_n-CaNAB--> A_B831*845*	0.037256 #^-1.s^-1	8 s^-1	2 s^-1	4.9706 uM	2 s^-1	4
A835*845*_B835*845* ---CaM_Ca_n-CaNAB--> A831*_B831*845*	0.037256 #^-1.s^-1	8 s^-1	2 s^-1	4.9706 uM	2 s^-1	4
A845*_B845* ---CaM_Ca_n-CaNAB--> A845*_B	0.037256 #^-1.s^-1	8 s^-1	2 s^-1	4.9706 uM	2 s^-1	4
A831*845*_B845* ---CaM_Ca_n-CaNAB--> A831*845*_B	0.037256 #^-1.s^-1	8 s^-1	2 s^-1	4.9706 uM	2 s^-1	4
A845*_B831*845* ---CaM_Ca_n-CaNAB--> A845*_B831*	0.037256 #^-1.s^-1	8 s^-1	2 s^-1	4.9706 uM	2 s^-1	4
A835*845*_B835*845* ---CaM_Ca_n-CaNAB--> A831*845*_B831*	0.037256 #^-1.s^-1	8 s^-1	2 s^-1	4.9706 uM	2 s^-1	4
A845*_B845* ---CaM_Ca_n-CaNAB--> A_B845*	0.037256 #^-1.s^-1	8 s^-1	2 s^-1	4.9706 uM	2 s^-1	4
A845*_B845* ---CaM_Ca_n-CaNAB--> A845*_B	0.037256 #^-1.s^-1	8 s^-1	2 s^-1	4.9706 uM	2 s^-1	4
A_B845* ---CaM_Ca_n-CaNAB--> A_B	0.037256 #^-1.s^-1	8 s^-1	2 s^-1	4.9706 uM	2 s^-1	4
A845*_B ---CaM_Ca_n-CaNAB--> A_B	0.037256 #^-1.s^-1	8 s^-1	2 s^-1	4.9706 uM	2 s^-1	4
A831*845*_B845* ---CaM_Ca_n-CaNAB--> A831*_B845*	0.037256 #^-1.s^-1	8 s^-1	2 s^-1	4.9706 uM	2 s^-1	4
A831*845*_B845* ---CaM_Ca_n-CaNAB--> A831*845*_B	0.037256 #^-1.s^-1	8 s^-1	2 s^-1	4.9706 uM	2 s^-1	4
A831*_B845* ---CaM_Ca_n-CaNAB--> A831*_B	0.037256 #^-1.s^-1	8 s^-1	2 s^-1	4.9706 uM	2 s^-1	4
A831*845*_B ---CaM_Ca_n-CaNAB--> A831*_B	0.037256 #^-1.s^-1	8 s^-1	2 s^-1	4.9706 uM	2 s^-1	4
A845*_B831*845* ---CaM_Ca_n-CaNAB--> A_B831*845*	0.037256 #^-1.s^-1	8 s^-1	2 s^-1	4.9706 uM	2 s^-1	4
A845*_B831*845* ---CaM_Ca_n-CaNAB--> A845*_B831*	0.037256 #^-1.s^-1	8 s^-1	2 s^-1	4.9706 uM	2 s^-1	4
A_B831*845* ---CaM_Ca_n-CaNAB--> A_B831*	0.037256 #^-1.s^-1	8 s^-1	2 s^-1	4.9706 uM	2 s^-1	4
A845*_B831* ---CaM_Ca_n-CaNAB--> A_B831*	0.037256 #^-1.s^-1	8 s^-1	2 s^-1	4.9706 uM	2 s^-1	4
A835*845*_B835*845* ---CaM_Ca_n-CaNAB--> A831*_B831*845*	0.037256 #^-1.s^-1	8 s^-1	2 s^-1	4.9706 uM	2 s^-1	4
A835*845*_B835*845* ---CaM_Ca_n-CaNAB--> A831*845*_B831*	0.037256 #^-1.s^-1	8 s^-1	2 s^-1	4.9706 uM	2 s^-1	4
A831*845*_B831* ---CaM_Ca_n-CaNAB--> A831*_B831*	0.037256 #^-1.s^-1	8 s^-1	2 s^-1	4.9706 uM	2 s^-1	4
A831*_B831*845* ---CaM_Ca_n-CaNAB--> A831*_B831*	0.037256 #^-1.s^-1	8 s^-1	2 s^-1	4.9706 uM	2 s^-1	4
neurogranin*_PSD ---CaM_Ca_n-CaNAB--> neurogranin_PSD	0.0061778 #^-1.s^-1	2.67 s^-1	0.67 s^-1	10.012 uM	0.67 s^-1	3.9851
cAMP-PDE ---PKA-active--> cAMP-PDE*	0.11111 #^-1.s^-1	36 s^-1	9 s^-1	7.5001 uM	9 s^-1	4
I1 ---PKA-active--> I1*	0.11111 #^-1.s^-1	36 s^-1	9 s^-1	7.5001 uM	9 s^-1	4
I1 ---PKA-active--> I1*	0.11111 #^-1.s^-1	36 s^-1	9 s^-1	7.5001 uM	9 s^-1	4
A_B ---PKA-active--> A845*_B	0.074072 #^-1.s^-1	24 s^-1	6 s^-1	7.5002 uM	6 s^-1	4
A831*_B ---PKA-active--> A831*845*_B	0.074072 #^-1.s^-1	24 s^-1	6 s^-1	7.5002 uM	6 s^-1	4
A_B831* ---PKA-active--> A845*_B831*	0.074072 #^-1.s^-1	24 s^-1	6 s^-1	7.5002 uM	6 s^-1	4
A831*_B831* ---PKA-active--> A831*845*_B831*	0.074072 #^-1.s^-1	24 s^-1	6 s^-1	7.5002 uM	6 s^-1	4
A_B ---PKA-active--> A_B845*	0.074072 #^-1.s^-1	24 s^-1	6 s^-1	7.5002 uM	6 s^-1	4
A831*_B ---PKA-active--> AA831*_B845*	0.074072 #^-1.s^-1	24 s^-1	6 s^-1	7.5002 uM	6 s^-1	4
A_B831* ---PKA-active--> A_B831*845*	0.074072 #^-1.s^-1	24 s^-1	6 s^-1	7.5002 uM	6 s^-1	4
A831*_B831* ---PKA-active--> A831*_B831*845*	0.074072 #^-1.s^-1	24 s^-1	6 s^-1	7.5002 uM	6 s^-1	4
A_B845* ---PKA-active--> A845*_B845*	0.074072 #^-1.s^-1	24 s^-1	6 s^-1	7.5002 uM	6 s^-1	4
AA831*_B845* ---PKA-active--> A831*845*_B845*	0.074072 #^-1.s^-1	24 s^-1	6 s^-1	7.5002 uM	6 s^-1	4
A_B831*845* ---PKA-active--> A845*_B831*845*	0.074072 #^-1.s^-1	24 s^-1	6 s^-1	7.5002 uM	6 s^-1	4
A831*_B831*845* ---PKA-active--> A835*845*_B835*845*	0.074072 #^-1.s^-1	24 s^-1	6 s^-1	7.5002 uM	6 s^-1	4
A845*_B ---PKA-active--> A845*_B845*	0.074072 #^-1.s^-1	24 s^-1	6 s^-1	7.5002 uM	6 s^-1	4
A831*845*_B ---PKA-active--> A831*845*_B845*	0.074072 #^-1.s^-1	24 s^-1	6 s^-1	7.5002 uM	6 s^-1	4
A845*_B831* ---PKA-active--> A845*_B831*845*	0.074072 #^-1.s^-1	24 s^-1	6 s^-1	7.5002 uM	6 s^-1	4
A831*845*_B831* ---PKA-active--> A835*845*_B835*845*	0.074072 #^-1.s^-1	24 s^-1	6 s^-1	7.5002 uM	6 s^-1	4
A_B845* ---PKA-active--> A845*_B845*	0.074072 #^-1.s^-1	24 s^-1	6 s^-1	7.5002 uM	6 s^-1	4
A845*_B ---PKA-active--> A845*_B845*	0.074072 #^-1.s^-1	24 s^-1	6 s^-1	7.5002 uM	6 s^-1	4
A_B ---PKA-active--> A845*_B	0.074072 #^-1.s^-1	24 s^-1	6 s^-1	7.5002 uM	6 s^-1	4
A_B ---PKA-active--> A_B845*	0.074072 #^-1.s^-1	24 s^-1	6 s^-1	7.5002 uM	6 s^-1	4
A831*_B845* ---PKA-active--> A831*845*_B845*	0.074072 #^-1.s^-1	24 s^-1	6 s^-1	7.5002 uM	6 s^-1	4
A831*845*_B ---PKA-active--> A831*845*_B845*	0.074072 #^-1.s^-1	24 s^-1	6 s^-1	7.5002 uM	6 s^-1	4
A831*_B ---PKA-active--> A831*_B845*	0.074072 #^-1.s^-1	24 s^-1	6 s^-1	7.5002 uM	6 s^-1	4
A831*_B ---PKA-active--> A831*845*_B	0.074072 #^-1.s^-1	24 s^-1	6 s^-1	7.5002 uM	6 s^-1	4
A_B831*845* ---PKA-active--> A845*_B831*845*	0.074072 #^-1.s^-1	24 s^-1	6 s^-1	7.5002 uM	6 s^-1	4
A845*_B831* ---PKA-active--> A845*_B831*845*	0.074072 #^-1.s^-1	24 s^-1	6 s^-1	7.5002 uM	6 s^-1	4
A_B831* ---PKA-active--> A_B831*845*	0.074072 #^-1.s^-1	24 s^-1	6 s^-1	7.5002 uM	6 s^-1	4
A_B831* ---PKA-active--> A845*_B831*	0.074072 #^-1.s^-1	24 s^-1	6 s^-1	7.5002 uM	6 s^-1	4
A831*_B831*845* ---PKA-active--> A835*845*_B835*845*	0.074072 #^-1.s^-1	24 s^-1	6 s^-1	7.5002 uM	6 s^-1	4
A831*845*_B831* ---PKA-active--> A835*845*_B835*845*	0.074072 #^-1.s^-1	24 s^-1	6 s^-1	7.5002 uM	6 s^-1	4
A831*_B831* ---PKA-active--> A831*_B831*845*	0.074072 #^-1.s^-1	24 s^-1	6 s^-1	7.5002 uM	6 s^-1	4
A831*_B831* ---PKA-active--> A831*845*_B831*	0.074072 #^-1.s^-1	24 s^-1	6 s^-1	7.5002 uM	6 s^-1	4
ATP ---AC1-CaM--> cAMP	0.0013889 #^-1.s^-1	18 s^-1	4.5 s^-1	300 uM	4.5 s^-1	4

ATP ---AC2*--> cAMP	0.00061728 #^-1.s^-1	8 s^-1	2 s^-1	300 uM	2 s^-1	4
cAMP ---cAMP-PDE--> AMP	0.046667 #^-1.s^-1	40 s^-1	10 s^-1	19.841 uM	10 s^-1	4
cAMP ---cAMP-PDE*--> AMP	0.093333 #^-1.s^-1	80 s^-1	20 s^-1	19.841 uM	20 s^-1	4
cAMP ---PDE1--> AMP	0.0038889 #^-1.s^-1	6.67 s^-1	1.667 s^-1	39.7 uM	1.667 s^-1	4.0012
cAMP ---CaM.PDE1--> AMP	0.023333 #^-1.s^-1	40 s^-1	10 s^-1	39.683 uM	10 s^-1	4
CaMKII-thr286-CaM-PSD ---PP1-active_PSD--> CaMKII-CaM-PSD	1.0417 #^-1.s^-1	10 s^-1	2.5 s^-1	1.9999 uM	2.5 s^-1	4
CaMKII-thr286-PSD ---PP1-active_PSD--> CaMKII-PSD	1.0417 #^-1.s^-1	10 s^-1	2.5 s^-1	1.9999 uM	2.5 s^-1	4
CaMKII***-PSD ---PP1-active_PSD--> CaMKII-thr286-PSD	0.40857 #^-1.s^-1	10 s^-1	2.5 s^-1	5.0991 uM	2.5 s^-1	4
CaMKII***-PSD ---PP1-active_PSD--> CaMKII-thr305-PSD	1.0417 #^-1.s^-1	10 s^-1	2.5 s^-1	1.9999 uM	2.5 s^-1	4
A845*_B845* ---PP1-active_PSD--> A_B845*	0.14583 #^-1.s^-1	0.68 s^-1	0.17 s^-1	0.97145 uM	0.17 s^-1	4
A845*_B845* ---PP1-active_PSD--> A845*_B	0.14583 #^-1.s^-1	0.68 s^-1	0.17 s^-1	0.97145 uM	0.17 s^-1	4
A845*_B ---PP1-active_PSD--> A_B	0.14583 #^-1.s^-1	0.68 s^-1	0.17 s^-1	0.97145 uM	0.17 s^-1	4
A_B845* ---PP1-active_PSD--> A_B	0.14583 #^-1.s^-1	0.68 s^-1	0.17 s^-1	0.97145 uM	0.17 s^-1	4
A831*845*_B845* ---PP1-active_PSD--> A831*_B845*	0.14583 #^-1.s^-1	0.68 s^-1	0.17 s^-1	0.97145 uM	0.17 s^-1	4
A831*845*_B845* ---PP1-active_PSD--> A831*845*_B	0.14583 #^-1.s^-1	0.68 s^-1	0.17 s^-1	0.97145 uM	0.17 s^-1	4
A831*_B845* ---PP1-active_PSD--> A831*_B	0.14583 #^-1.s^-1	0.68 s^-1	0.17 s^-1	0.97145 uM	0.17 s^-1	4
A831*845*_B ---PP1-active_PSD--> A831*_B	0.14583 #^-1.s^-1	0.68 s^-1	0.17 s^-1	0.97145 uM	0.17 s^-1	4
A831*845*_B845* ---PP1-active_PSD--> A845*_B845*	0.14583 #^-1.s^-1	1.4 s^-1	0.35 s^-1	2 uM	0.35 s^-1	4
A831*_B845* ---PP1-active_PSD--> A_B845*	0.14583 #^-1.s^-1	1.4 s^-1	0.35 s^-1	2 uM	0.35 s^-1	4
A831*845*_B ---PP1-active_PSD--> A845*_B	0.14583 #^-1.s^-1	1.4 s^-1	0.35 s^-1	2 uM	0.35 s^-1	4
A831*_B ---PP1-active_PSD--> A_B	0.14583 #^-1.s^-1	1.4 s^-1	0.35 s^-1	2 uM	0.35 s^-1	4
A845*_B831*845* ---PP1-active_PSD--> A_B831*845*	0.14583 #^-1.s^-1	0.68 s^-1	0.17 s^-1	0.97145 uM	0.17 s^-1	4
A845*_B831*845* ---PP1-active_PSD--> A845*_B831*	0.14583 #^-1.s^-1	0.68 s^-1	0.17 s^-1	0.97145 uM	0.17 s^-1	4
A_B831*845* ---PP1-active_PSD--> A_B831*	0.14583 #^-1.s^-1	0.68 s^-1	0.17 s^-1	0.97145 uM	0.17 s^-1	4
A845*_B831* ---PP1-active_PSD--> A_B831*	0.14583 #^-1.s^-1	0.68 s^-1	0.17 s^-1	0.97145 uM	0.17 s^-1	4
A835*845*_B835*845* ---PP1-active_PSD--> A831*_B831*845*	0.14583 #^-1.s^-1	0.68 s^-1	0.17 s^-1	0.97145 uM	0.17 s^-1	4
A835*845*_B835*845* ---PP1-active_PSD--> A831*845*_B831*	0.14583 #^-1.s^-1	0.68 s^-1	0.17 s^-1	0.97145 uM	0.17 s^-1	4
A831*_B831*845* ---PP1-active_PSD--> A831*_B831*	0.14583 #^-1.s^-1	0.68 s^-1	0.17 s^-1	0.97145 uM	0.17 s^-1	4
A831*845*_B831* ---PP1-active_PSD--> A831*_B831*	0.14583 #^-1.s^-1	0.68 s^-1	0.17 s^-1	0.97145 uM	0.17 s^-1	4
A_B831*845* ---PP1-active_PSD--> A_B845*	0.14583 #^-1.s^-1	1.4 s^-1	0.35 s^-1	2 uM	0.35 s^-1	4
A845*_B831*845* ---PP1-active_PSD--> A845*_B845*	0.14583 #^-1.s^-1	1.4 s^-1	0.35 s^-1	2 uM	0.35 s^-1	4
A845*_B831* ---PP1-active_PSD--> A845*_B	0.14583 #^-1.s^-1	1.4 s^-1	0.35 s^-1	2 uM	0.35 s^-1	4
A_B831* ---PP1-active_PSD--> A_B	0.14583 #^-1.s^-1	1.4 s^-1	0.35 s^-1	2 uM	0.35 s^-1	4
A835*845*_B835*845* ---PP1-active_PSD--> A845*_B831*845*	0.14583 #^-1.s^-1	1.4 s^-1	0.35 s^-1	2 uM	0.35 s^-1	4
A831*_B831*845* ---PP1-active_PSD--> A_B831*845*	0.14583 #^-1.s^-1	1.4 s^-1	0.35 s^-1	2 uM	0.35 s^-1	4
A831*845*_B831* ---PP1-active_PSD--> A845*_B831*	0.14583 #^-1.s^-1	1.4 s^-1	0.35 s^-1	2 uM	0.35 s^-1	4
A831*_B831* ---PP1-active_PSD--> A_B831*	0.14583 #^-1.s^-1	1.4 s^-1	0.35 s^-1	2 uM	0.35 s^-1	4
A835*845*_B835*845* ---PP1-active_PSD--> A831*845*_B845*	0.14583 #^-1.s^-1	1.4 s^-1	0.35 s^-1	2 uM	0.35 s^-1	4
A831*_B831*845* ---PP1-active_PSD--> A831*_B845*	0.14583 #^-1.s^-1	1.4 s^-1	0.35 s^-1	2 uM	0.35 s^-1	4
A831*845*_B831* ---PP1-active_PSD--> A831*845*_B	0.14583 #^-1.s^-1	1.4 s^-1	0.35 s^-1	2 uM	0.35 s^-1	4
A831*_B831* ---PP1-active_PSD--> A831*_B	0.14583 #^-1.s^-1	1.4 s^-1	0.35 s^-1	2 uM	0.35 s^-1	4
CaMKII-thr305-PSD ---PP1-active_PSD--> CaMKII-PSD	0.40857 #^-1.s^-1	10 s^-1	2.5 s^-1	5.0991 uM	2.5 s^-1	4

Pools for group /##[]

name	InitialConc	buffered	Volume
Ca	0.08 uM	0	0.09 fl
Ca-PSD	0.08 uM	0	0.01 fl
PKC-active	0.1 uM	0	0.09 fl
PKC-control	0.1 uM	1	0.09 fl
Ca_control_cyt	0.08 uM	1	0.09 fl
Ca_control_PSD	0.08 uM	1	0.01 fl
CaMKII	20 uM	0	0.09 fl
CaMKII-CaM	0 uM	0	0.09 fl
CaMKII-thr286*-CaM	0 uM	0	0.09 fl
CaMKII***	0 uM	0	0.09 fl
CaMKII-thr286	0 uM	0	0.09 fl
tot_CaM_CaMKII	0 uM	0	0.09 fl
tot_autonomous_CaMKII	2 uM	0	0.09 fl
CaMK-thr305	0 uM	0	0.09 fl
tot_CaMKII_cyt	22 uM	0	0.09 fl
act_CaMKII_cyt	2 uM	0	0.09 fl
basal_CaMKII_cyt	2 uM	1	0.09 fl
basal_CaMKII_PSD_control	2 uM	1	0.01 fl
CaMKII-thr305-PSD	0 uM	0	0.01 fl
CaMKII***-PSD	0 uM	0	0.01 fl
CaMKII-PSD	0 uM	0	0.01 fl
NMDAR	120 uM	0	0.01 fl
CaMKII-thr286-PSD	0 uM	0	0.01 fl
CaMKII-CaM-PSD	0 uM	0	0.01 fl
CaMKII-thr286-CaM-PSD	0 uM	0	0.01 fl
tot-auto-PSD	2 uM	0	0.01 fl
basal_CaMKII_PSD	2 uM	0	0.01 fl
tot_CaMKII_PSD	2 uM	0	0.01 fl
actCaMKII-PSD	2 uM	0	0.01 fl
tot-CaM-CaMKII-PSD	0 uM	0	0.01 fl
286P-PSD	0 uM	0	0.01 fl
CaM	26.333 uM	0	0.09 fl
neurogranin-CaM	0 uM	0	0.09 fl
neurogranin*	0 uM	0	0.09 fl
neurogranin	10 uM	0	0.09 fl
CaM-PSD	26.333 uM	0	0.01 fl
neurogranin-CaM_PSD	0 uM	0	0.01 fl
neurogranin_PSD	10 uM	0	0.01 fl
neurogranin*_PSD	0 uM	0	0.01 fl
CaM-TR2-Ca2	0 uM	0	0.09 fl
CaM-Ca3	0 uM	0	0.09 fl
CaM-Ca4-PSD	0 uM	0	0.01 fl
CaM-Ca3-PSD	0 uM	0	0.01 fl
CaM-TR2-Ca2-PSD	0 uM	0	0.01 fl
CaM-Ca4	0 uM	0	0.09 fl
I1	1.8 uM	0	0.09 fl
I1*	0 uM	0	0.09 fl

PP1-I1*	0 uM	0	0.09 fl
PP1-I1	0 uM	0	0.09 fl
PP2A	0.11111 uM	0	0.09 fl
PP1-active	1.8 uM	0	0.09 fl
CaNAB	1 uM	0	0.09 fl
CaNAB-Ca2	0 uM	0	0.09 fl
CaNAB-Ca4	0 uM	0	0.09 fl
CaM_Ca_n-CaNAB	0 uM	0	0.09 fl
R2C2	0.5 uM	0	0.09 fl
R2C2-cAMP	0 uM	0	0.09 fl
R2C2-cAMP2	0 uM	0	0.09 fl
R2C2-cAMP3	0 uM	0	0.09 fl
R2C2-cAMP4	0 uM	0	0.09 fl
R2C-cAMP4	0 uM	0	0.09 fl
R2-cAMP4	0 uM	0	0.09 fl
PKA-inhibitor	0.25926 uM	0	0.09 fl
inhibited-PKA	0 uM	0	0.09 fl
PKA-active	0 uM	0	0.09 fl
ATP	2000 uM	1	0.09 fl
AC1-CaM	0 uM	0	0.09 fl
AC1	0.074074 uM	0	0.09 fl
AC2*	0 uM	0	0.09 fl
AC2	0.074074 uM	0	0.09 fl
AMP	0 uM	0	0.09 fl
cAMP-PDE	0.55556 uM	0	0.09 fl
cAMP-PDE*	0 uM	0	0.09 fl
PDE1	2.5926 uM	0	0.09 fl
CaM.PDE1	0 uM	0	0.09 fl
cAMP_in_dend	0 uM	0	5 fl
cAMP	0 uM	0	0.09 fl
I1	4 uM	0	0.01 fl
I1*	0 uM	0	0.01 fl
PP1-I1*	0 uM	0	0.01 fl
PP1-I1	0 uM	0	0.01 fl
PP1-active_PSD	4 uM	0	0.01 fl
GluR23_I	0.092593 uM	0	0.09 fl
AMPAR_deg	0 uM	1	0.09 fl
A_B	0 uM	0	0.09 fl
A831*_B	0 uM	0	0.09 fl
A845*_B	0 uM	0	0.09 fl
A831*845*_B	0 uM	0	0.09 fl
A_B845*	0 uM	0	0.09 fl
AA831*_B845*	0 uM	0	0.09 fl
A845*_B845*	0 uM	0	0.09 fl
A831*845*_B845*	0 uM	0	0.09 fl
A845*_B831*845*	0 uM	0	0.09 fl
A835*845*_B835*845*	0 uM	0	0.09 fl
A_B831*845*	0 uM	0	0.09 fl
A831*_B831*845*	0 uM	0	0.09 fl
A_B831*	0 uM	0	0.09 fl
A845*_B831*	0 uM	0	0.01 fl
A831*_B831*	0 uM	0	0.09 fl
A831*845*_B831*	0 uM	0	0.09 fl
AMPAR_bulk	0.011111 uM	1	5 fl
I_845	0 uM	0	0.09 fl
I_845-P	0 uM	0	0.09 fl
I_845_PP	0 uM	0	0.09 fl
tot_I_GluR12	0 uM	0	0.09 fl
total_Int	0.096296 uM	0	0.09 fl
A_B	0 uM	0	0.01 fl
A831*_B	0 uM	0	0.01 fl
A_B831*	0 uM	0	0.01 fl
A831*_B831*	0 uM	0	0.01 fl
A845*_B	0 uM	0	0.01 fl
A831*845*_B	0 uM	0	0.01 fl
A831*845*_B831*	0 uM	0	0.01 fl
A_B845*	0 uM	0	0.01 fl
A845*_B831*	0 uM	0	0.01 fl
A845*_B845*	0 uM	0	0.01 fl
A831*_B845*	0 uM	0	0.01 fl
A_B831*845*	0 uM	0	0.01 fl
A831*_B831*845*	0 uM	0	0.01 fl
A831*845*_B845*	0 uM	0	0.01 fl
A845*_B831*845*	0 uM	0	0.01 fl
A835*845*_B835*845*	0 uM	0	0.01 fl
GluR23_M	3.5 uM	0	0.01 fl
Ser845-PP	0 uM	0	0.01 fl
Ser845-P	0 uM	0	0.01 fl
Ser845	0 uM	0	0.01 fl
tot_mem_GluR12	0 uM	0	0.01 fl
total_mem	3.4667 uM	0	0.01 fl
Ser831	0 uM	0	0.01 fl
Ser831-P	0 uM	0	0.01 fl
Ser831-PP	0 uM	0	0.01 fl
Anchor	27.333 uM	0	0.01 fl

Model Parameters for simplified AMPAR bistability model: Model 2.

Concentration units: uM (micromolar) for rate constants presented as Kf, Kb, Km
#/cell for rate constants presented as kf, kb, k1, k2, k3. This formulation of rates may depend on cellular volume.
Molecular traffic between compartments of different volumes is represented as reactions. These reactions may have concentration terms which are ambiguous if expressed in uM. Please use the concentration terms involving #/cell for such reactions.
Time units: Seconds in all cases.
Total Volume of Synapse = 0.1 femtoliters (fl)
Volume of cytosolic portion = 0.09 fl
Volume of Postsynaptic Density (PSD) = 0.01 fl

The enzyme rates are related as follows:
Km = (k2 + k3)/k1 (after conversion of units)
Kcat = k3.
Ratio = k2/k3

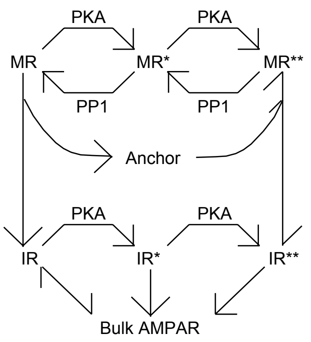

Reactions

Reaction	kf	kb	Kf	Kb	
Anchor + IR** <===> MR**	0.0002 #^-1.s^-1	0.008 s^-1	0.0012 uM^-1.s^-1	0.008 s^-1	Traffic Reacn.
MR <===> Anchor + IR	0.0008 s^-1	0 #^-1.s^-1	0.0008 s^-1	0 uM^-1.s^-1	Traffic Reacn.
Bulk_AMPAR <===> IR	0.018 s^-1	1 s^-1	0.018 s^-1	1 s^-1	Traffic Reacn.
IR** <===> Bulk_AMPAR	2e-05 s^-1	0 s^-1	2e-05 s^-1	0 s^-1	
IR* <===> Bulk_AMPAR	2e-05 s^-1	0 s^-1	2e-05 s^-1	0 s^-1	

Enzyme activities

Enzyme-reaction	k1	k2	k3	Km	kcat	ratio
MR ---PKA-active--> MR*	0.14815 #^-1.s^-1	24 s^-1	6 s^-1	3.75 uM	6 s^-1	4
IR ---PKA-active--> IR*	0.14815 #^-1.s^-1	24 s^-1	6 s^-1	3.75 uM	6 s^-1	4
MR* ---PKA-active--> MR**	0.14815 #^-1.s^-1	24 s^-1	6 s^-1	3.75 uM	6 s^-1	4
IR* ---PKA-active--> IR**	0.14815 #^-1.s^-1	24 s^-1	6 s^-1	3.75 uM	6 s^-1	4
MR* ---PP1-active--> MR	0.29167 #^-1.s^-1	1.4 s^-1	0.35 s^-1	0.99999 uM	0.35 s^-1	4
MR** ---PP1-active--> MR*	0.29167 #^-1.s^-1	1.4 s^-1	0.35 s^-1	0.99999 uM	0.35 s^-1	4

Pools

name	InitialConc	buffered	Volume
PKA-active	0.018519 uM	1	0.09 fl
Anchor	27.333 uM	0	0.01 fl
Bulk_AMPAR	0.003 uM	1	5 fl
PP1-active	0.33333 uM	0	0.01 fl
MR*	0 uM	0	0.01 fl
MR	0 uM	0	0.01 fl
MR**	0 uM	0	0.01 fl
IR**	0 uM	0	0.09 fl
IR	0 uM	0	0.09 fl
IR*	0 uM	0	0.09 fl

Model Parameters for CaMKII bistability model: Model 3.

Concentration units: uM (micromolar) for rate constants presented as Kf, Kb, Km
#/cell for rate constants presented as kf, kb, k1, k2, k3. This formulation of rates may depend on cellular volume.
A few trafficking reactions have concentration units. In such cases the preferred rates are those using units of # per cell, as these remain unambiguous even when the volume terms differ for reactants.
Time units: Seconds in all cases.
Total Volume of Synapse = 0.1 femtoliters (fl)
Volume of cytosolic portion = 0.09 fl
Volume of Postsynaptic Density (PSD) = 0.01 fl

The enzyme rates are related as follows:
Km = (k2 + k3)/k1 (after conversion of units)
Kcat = k3.
Ratio = k2/k3

Initial concentrations (CoInit) are mostly zero, except for a few key molecules.
There is a flag for 'buffered' in the molecule concentration table. When this flag is zero the molecule concentrations are computed according to the reaction equations. If the flag is one the molecule concentration is held fixed to its initial concentration.

The entire model scheme is presented as composite tables for molecules, reactions and enzymes.

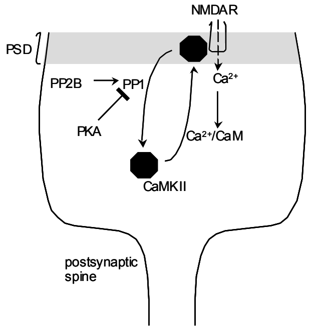

Block Diagram. Only the CaMKII traffic and regulatory molecules are implemented in this model.

CaMKII reactions

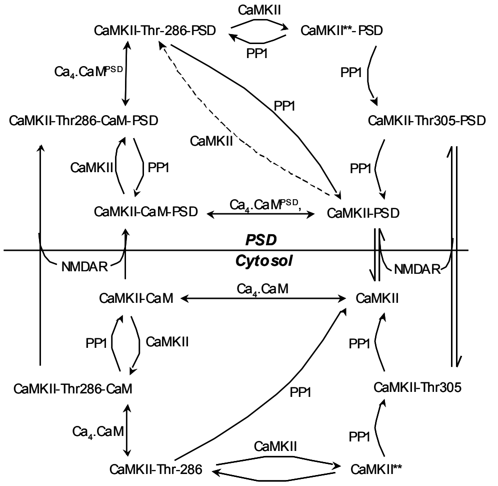

Equations for all groups

Reactions

Reaction	kf	kb	Kf	Kb	
CaMKII-PSD + CaM-Ca4-PSD <===> CaMKII-CaM-PSD	8.3333 #^-1.s^-1	0 s^-1	50 uM^-1.s^-1	0 s^-1	
CaMKII-CaM + NMDAR <===> CaMKII-CaM-PSD	2e-05 #^-1.s^-1	0 s^-1	0.00108 uM^-1.s^-1	0 s^-1	Trafficking reac
CaMKII-thr286*-CaM + NMDAR <===> CaMKII-thr286-CaM-PSD	2e-05 #^-1.s^-1	0 s^-1	0.00108 uM^-1.s^-1	0 s^-1	Trafficking reac
CaMKII-PSD <===> CaMKII + NMDAR	0.3 s^-1	1e-05 #^-1.s^-1	0.3 s^-1	6e-05 uM^-1.s^-1	Trafficking reac
CaMKII-thr305-PSD <===> CaMK-thr305 + NMDAR	0.3 s^-1	1e-05 #^-1.s^-1	0.3 s^-1	6e-05 uM^-1.s^-1	Trafficking reac
CaMKII-thr286-PSD + CaM-Ca4-PSD <===> CaMKII-thr286-CaM-PSD	166.67 #^-1.s^-1	0.1 s^-1	1000 uM^-1.s^-1	0.1 s^-1	
CaM-Ca4-PSD <===> CaM-Ca4	540 s^-1	60 s^-1	540 s^-1	60 s^-1	Trafficking reac
I1* + PP1-active_PSD <===> PP1-I1*	83.33 #^-1.s^-1	0.1 s^-1	499.98 uM^-1.s^-1	0.1 s^-1	
CaM-Ca3 + Ca <===> CaM-Ca4	0.0086111 #^-1.s^-1	10 s^-1	0.465 uM^-1.s^-1	10 s^-1	
CaMKII-CaM-PSD <===> CaM-Ca4-PSD + CaMKII-PSD	5 s^-1	0 #^-1.s^-1	5 s^-1	0 uM^-1.s^-1	
Ca_control_cyt <===> Ca	100 s^-1	100 s^-1	100 s^-1	100 s^-1	
Ca_control_PSD <===> Ca-PSD	100 s^-1	100 s^-1	100 s^-1	100 s^-1	
basal_CaMKII_PSD_control <===> basal_CaMKII_PSD	1 s^-1	1 s^-1	1 s^-1	1 s^-1	
PKC-control <===> PKC-active	2.5 s^-1	2.5 s^-1	2.5 s^-1	2.5 s^-1	
CaM-Ca4 + CaMKII <===> CaMKII-CaM	0.92592 #^-1.s^-1	5 s^-1	50 uM^-1.s^-1	5 s^-1	
CaMKII-thr286 + CaM-Ca4 <===> CaMKII-thr286*-CaM	18.522 #^-1.s^-1	0.1 s^-1	1000.2 uM^-1.s^-1	0.1 s^-1	
CaM + 2 Ca <===> CaM-TR2-Ca2	0.024691 #^-2.s^-1	72 s^-1	71.999 uM^-2.s^-1	72 s^-1	
CaM-TR2-Ca2 + Ca <===> CaM-Ca3	0.066667 #^-1.s^-1	10 s^-1	3.6 uM^-1.s^-1	10 s^-1	
CaM-PSD + 2 Ca-PSD <===> CaM-TR2-Ca2-PSD	2 #^-2.s^-1	72 s^-1	72 uM^-2.s^-1	72 s^-1	
CaM-TR2-Ca2-PSD + Ca-PSD <===> CaM-Ca3-PSD	0.6 #^-1.s^-1	10 s^-1	3.6 uM^-1.s^-1	10 s^-1	
CaM-Ca3-PSD + Ca-PSD <===> CaM-Ca4-PSD	0.077502 #^-1.s^-1	10 s^-1	0.46501 uM^-1.s^-1	10 s^-1	
I1* + PP1-active <===> PP1-I1*	9.2589 #^-1.s^-1	0.1 s^-1	499.98 uM^-1.s^-1	0.1 s^-1	
PP1-I1 <===> PP1-active + I1	1 s^-1	0 #^-1.s^-1	1 s^-1	0 uM^-1.s^-1	
2 Ca + CaNAB-Ca2 <===> CaNAB-Ca4	0.0012346 #^-2.s^-1	1 s^-1	3.6001 uM^-2.s^-1	1 s^-1	
CaNAB + 2 Ca <===> CaNAB-Ca2	3.4321 #^-2.s^-1	1 s^-1	10008 uM^-2.s^-1	1 s^-1	
CaM-Ca4 + CaNAB-Ca4 <===> CaM_Ca_n-CaNAB	11.111 #^-1.s^-1	1 s^-1	599.99 uM^-1.s^-1	1 s^-1	
PP1-I1 <===> I1 + PP1-active_PSD	1 s^-1	0 #^-1.s^-1	1 s^-1	0 uM^-1.s^-1	
CaM-Ca4 + AC1 <===> AC1-CaM	0.92592 #^-1.s^-1	1 s^-1	50 uM^-1.s^-1	1 s^-1	
AC2* <===> AC2	0.1 s^-1	0 s^-1	0.1 s^-1	0 s^-1	
cAMP-PDE* <===> cAMP-PDE	0.01 s^-1	0 s^-1	0.01 s^-1	0 s^-1	
PDE1 + CaM-Ca4 <===> CaM.PDE1	13.333 #^-1.s^-1	5 s^-1	719.98 uM^-1.s^-1	5 s^-1	
cAMP <===> cAMP_in_dend	300 s^-1	5.4 s^-1	300 s^-1	5.4 s^-1	
R2C2 + cAMP <===> R2C2-cAMP	1 #^-1.s^-1	33 s^-1	54 uM^-1.s^-1	33 s^-1	
R2C2-cAMP + cAMP <===> R2C2-cAMP2	1 #^-1.s^-1	33 s^-1	54 uM^-1.s^-1	33 s^-1	
R2C2-cAMP2 + cAMP <===> R2C2-cAMP3	1.3889 #^-1.s^-1	110 s^-1	75.001 uM^-1.s^-1	110 s^-1	
cAMP + R2C2-cAMP3 <===> R2C2-cAMP4	1.3889 #^-1.s^-1	32.5 s^-1	75.001 uM^-1.s^-1	32.5 s^-1	
R2C2-cAMP4 <===> PKA-active + R2C-cAMP4	60 s^-1	0.33333 #^-1.s^-1	60 s^-1	18 uM^-1.s^-1	
R2C-cAMP4 <===> PKA-active + R2-cAMP4	60 s^-1	0.33333 #^-1.s^-1	60 s^-1	18 uM^-1.s^-1	
PKA-active + PKA-inhibitor <===> inhibited-PKA	1.1111 #^-1.s^-1	1 s^-1	59.999 uM^-1.s^-1	1 s^-1	

Enzymes for group /##[]

Enzyme-reaction	k1	k2	k3	Km	kcat	ratio
CaMKII-thr286*-CaM ---PP1-active--> CaMKII-CaM	0.045397 #^-1.s^-1	10 s^-1	2.5 s^-1	5.099 uM	2.5 s^-1	4
CaMKII-thr286 ---PP1-active--> CaMKII	0.045397 #^-1.s^-1	10 s^-1	2.5 s^-1	5.099 uM	2.5 s^-1	4
CaMKII*** ---PP1-active--> CaMKII-thr286	0.045397 #^-1.s^-1	10 s^-1	2.5 s^-1	5.099 uM	2.5 s^-1	4
CaMKII*** ---PP1-active--> CaMK-thr305	0.045397 #^-1.s^-1	10 s^-1	2.5 s^-1	5.099 uM	2.5 s^-1	4
CaMK-thr305 ---PP1-active--> CaMKII	0.045397 #^-1.s^-1	10 s^-1	2.5 s^-1	5.099 uM	2.5 s^-1	4
I1 ---PKA-active--> I1*	0.11111 #^-1.s^-1	36 s^-1	9 s^-1	7.5001 uM	9 s^-1	4
I1 ---PKA-active--> I1*	0.11111 #^-1.s^-1	36 s^-1	9 s^-1	7.5001 uM	9 s^-1	4
cAMP-PDE ---PKA-active--> cAMP-PDE*	0.11111 #^-1.s^-1	36 s^-1	9 s^-1	7.5001 uM	9 s^-1	4
I1* ---PP2A--> I1	0.01196 #^-1.s^-1	8.3334 s^-1	2 s^-1	16 uM	2 s^-1	4.1667
PP1-I1* ---PP2A--> PP1-I1	0.01196 #^-1.s^-1	8.3334 s^-1	2 s^-1	16 uM	2 s^-1	4.1667
I1* ---PP2A--> I1	0.01196 #^-1.s^-1	8.3334 s^-1	2 s^-1	16 uM	2 s^-1	4.1667
PP1-I1* ---PP2A--> PP1-I1	0.01196 #^-1.s^-1	8.3334 s^-1	2 s^-1	16 uM	2 s^-1	4.1667
I1* ---CaNAB-Ca4--> I1	0.00063333 #^-1.s^-1	0.136 s^-1	0.034 s^-1	4.9708 uM	0.034 s^-1	4
I1* ---CaNAB-Ca4--> I1	0.00063334 #^-1.s^-1	0.136 s^-1	0.034 s^-1	4.9707 uM	0.034 s^-1	4
CaMKII-thr286-PSD ---tot-auto-PSD--> CaMKII***-PSD	0.01 #^-1.s^-1	24 s^-1	6 s^-1	500 uM	6 s^-1	4
CaMKII-CaM-PSD ---tot-auto-PSD--> CaMKII-thr286-CaM-PSD	0.00083333 #^-1.s^-1	2 s^-1	0.5 s^-1	500 uM	0.5 s^-1	4
CaMKII-PSD ---tot-auto-PSD--> CaMKII-thr286-PSD	0.0033333 #^-1.s^-1	8 s^-1	2 s^-1	500.01 uM	2 s^-1	4
CaMKII-thr286-CaM-PSD ---PP1-active_PSD--> CaMKII-CaM-PSD	0.083333 #^-1.s^-1	0.8 s^-1	0.2 s^-1	2 uM	0.2 s^-1	4
CaMKII-thr286-PSD ---PP1-active_PSD--> CaMKII-PSD	0.083333 #^-1.s^-1	0.8 s^-1	0.2 s^-1	2 uM	0.2 s^-1	4
CaMKII***-PSD ---PP1-active_PSD--> CaMKII-thr286-PSD	0.083333 #^-1.s^-1	0.8 s^-1	0.2 s^-1	2 uM	0.2 s^-1	4
CaMKII***-PSD ---PP1-active_PSD--> CaMKII-thr305-PSD	0.083333 #^-1.s^-1	0.8 s^-1	0.2 s^-1	2 uM	0.2 s^-1	4
CaMKII-thr305-PSD ---PP1-active_PSD--> CaMKII-PSD	0.083333 #^-1.s^-1	0.8 s^-1	0.2 s^-1	2 uM	0.2 s^-1	4
I1* ---CaM_Ca_n-CaNAB--> I1	0.0063333 #^-1.s^-1	1.36 s^-1	0.34 s^-1	4.9708 uM	0.34 s^-1	4
PP1-I1* ---CaM_Ca_n-CaNAB--> PP1-I1	0.0063333 #^-1.s^-1	1.36 s^-1	0.34 s^-1	4.9708 uM	0.34 s^-1	4
I1* ---CaM_Ca_n-CaNAB--> I1	0.0063334 #^-1.s^-1	1.36 s^-1	0.34 s^-1	4.9707 uM	0.34 s^-1	4
PP1-I1* ---CaM_Ca_n-CaNAB--> PP1-I1	0.0063334 #^-1.s^-1	1.36 s^-1	0.34 s^-1	4.9707 uM	0.34 s^-1	4
CaMKII-thr286-PSD ---tot-CaM-CaMKII-PSD--> CaMKII***-PSD	0.015625 #^-1.s^-1	24 s^-1	6 s^-1	320 uM	6 s^-1	4
CaMKII-CaM-PSD ---tot-CaM-CaMKII-PSD--> CaMKII-thr286-CaM-PSD	0.0013021 #^-1.s^-1	2 s^-1	0.5 s^-1	320 uM	0.5 s^-1	4
CaMKII-PSD ---tot-CaM-CaMKII-PSD--> CaMKII-thr286-PSD	0.0052083 #^-1.s^-1	8 s^-1	2 s^-1	320 uM	2 s^-1	4
AC2 ---PKC-active--> AC2*	0.011111 #^-1.s^-1	16 s^-1	4 s^-1	33.334 uM	4 s^-1	4
CaMKII-thr286 ---tot_CaM_CaMKII--> CaMKII***	0.0024474 #^-1.s^-1	24 s^-1	6 s^-1	227 uM	6 s^-1	4
CaMKII-CaM ---tot_CaM_CaMKII--> CaMKII-thr286*-CaM	0.00020395 #^-1.s^-1	2 s^-1	0.5 s^-1	227 uM	0.5 s^-1	4
CaMKII-thr286 ---tot_autonomous_CaMKII--> CaMKII***	0.0015873 #^-1.s^-1	24 s^-1	6 s^-1	350 uM	6 s^-1	4
CaMKII-CaM ---tot_autonomous_CaMKII--> CaMKII-thr286*-CaM	0.00013228 #^-1.s^-1	2 s^-1	0.5 s^-1	349.99 uM	0.5 s^-1	4
ATP ---AC1-CaM--> cAMP	0.0013889 #^-1.s^-1	18 s^-1	4.5 s^-1	300 uM	4.5 s^-1	4
ATP ---AC2*--> cAMP	0.00061728 #^-1.s^-1	8 s^-1	2 s^-1	300 uM	2 s^-1	4
cAMP ---cAMP-PDE--> AMP	0.046667 #^-1.s^-1	40 s^-1	10 s^-1	19.841 uM	10 s^-1	4
cAMP ---cAMP-PDE*--> AMP	0.093333 #^-1.s^-1	80 s^-1	20 s^-1	19.841 uM	20 s^-1	4
cAMP ---PDE1--> AMP	0.0038889 #^-1.s^-1	6.67 s^-1	1.667 s^-1	39.7 uM	1.667 s^-1	4.0012
cAMP ---CaM.PDE1--> AMP	0.023333 #^-1.s^-1	40 s^-1	10 s^-1	39.683 uM	10 s^-1	4

Pools for group /##[]

name	InitialConc	buffered	Volume
CaM-Ca4	0 uM	0	0.09 fl
PP1-active	1.8 uM	0	0.09 fl
Ca	0.08 uM	0	0.09 fl
PKA-active	0 uM	0	0.09 fl
CaM-Ca3	0 uM	0	0.09 fl
CaM-TR2-Ca2	0 uM	0	0.09 fl
PP2A	0.11111 uM	0	0.09 fl
CaNAB-Ca4	0 uM	0	0.09 fl
CaMKII-thr286-CaM-PSD	0 uM	0	0.01 fl
CaMKII-CaM-PSD	0 uM	0	0.01 fl
CaMKII-thr286-PSD	0 uM	0	0.01 fl
CaMKII-PSD	0 uM	0	0.01 fl
CaMKII***-PSD	0 uM	0	0.01 fl
tot-auto-PSD	2 uM	0	0.01 fl
CaM-TR2-Ca2-PSD	0 uM	0	0.01 fl
CaM-Ca3-PSD	0.0025458 uM	0	0.01 fl
CaM-Ca4-PSD	0 uM	0	0.01 fl
Ca-PSD	0.08 uM	0	0.01 fl
286P-PSD	0 uM	0	0.01 fl
actCaMKII-PSD	2 uM	0	0.01 fl
tot_CaMKII_PSD	2 uM	0	0.01 fl
tot_CaMKII_cyt	22 uM	0	0.09 fl
PP1-active_PSD	4 uM	0	0.01 fl
act_CaMKII_cyt	2 uM	0	0.09 fl
NMDAR	120 uM	0	0.01 fl
CaM_Ca_n-CaNAB	0 uM	0	0.09 fl
basal_CaMKII_cyt	2 uM	1	0.09 fl
basal_CaMKII_PSD	2 uM	0	0.01 fl
Ca_control_cyt	0.08 uM	1	0.09 fl
Ca_control_PSD	0.08 uM	1	0.01 fl
basal_CaMKII_PSD_control	2 uM	1	0.01 fl
tot-CaM-CaMKII-PSD	0 uM	0	0.01 fl
cAMP	0 uM	0	0.09 fl
PKC-control	0.1 uM	1	0.09 fl
PKC-active	0.1 uM	0	0.09 fl
CaMKII-thr305-PSD	0 uM	0	0.01 fl
CaMKII	20 uM	0	0.09 fl
CaMKII-CaM	0 uM	0	0.09 fl
CaMKII-thr286*-CaM	0 uM	0	0.09 fl
CaMKII***	0 uM	0	0.09 fl
CaMKII-thr286	0 uM	0	0.09 fl
tot_CaM_CaMKII	0 uM	0	0.09 fl
tot_autonomous_CaMKII	2 uM	0	0.09 fl
CaMK-thr305	0 uM	0	0.09 fl
CaM	26.333 uM	0	0.09 fl
CaM-PSD	26.333 uM	0	0.01 fl
I1	1.8 uM	0	0.09 fl
I1*	0 uM	0	0.09 fl
PP1-I1*	0 uM	0	0.09 fl
PP1-I1	0 uM	0	0.09 fl
CaNAB	1 uM	0	0.09 fl
CaNAB-Ca2	0 uM	0	0.09 fl
I1	4 uM	0	0.01 fl
I1*	0 uM	0	0.01 fl
PP1-I1*	0 uM	0	0.01 fl
PP1-I1	0 uM	0	0.01 fl
ATP	2000 uM	1	0.09 fl
AC1-CaM	0 uM	0	0.09 fl
AC1	0.074074 uM	0	0.09 fl
AC2*	0 uM	0	0.09 fl
AC2	0.074074 uM	0	0.09 fl
AMP	0 uM	0	0.09 fl
cAMP-PDE	0.55556 uM	0	0.09 fl
cAMP-PDE*	0 uM	0	0.09 fl
PDE1	2.5926 uM	0	0.09 fl
CaM.PDE1	0 uM	0	0.09 fl
cAMP_in_dend	0 uM	0	5 fl
R2C2	0.5 uM	0	0.09 fl
R2C2-cAMP	0 uM	0	0.09 fl
R2C2-cAMP2	0 uM	0	0.09 fl
R2C2-cAMP3	0 uM	0	0.09 fl
R2C2-cAMP4	0 uM	0	0.09 fl
R2C-cAMP4	0 uM	0	0.09 fl
R2-cAMP4	0 uM	0	0.09 fl
PKA-inhibitor	0.25926 uM	0	0.09 fl
inhibited-PKA	0 uM	0	0.09 fl

Model Parameters for model where AMPAR and CaMKII bistability occurs in lockstep: Model 4.

Concentration units: uM (micromolar) for rate constants presented as Kf, Kb, Km
#/cell for rate constants presented as kf, kb, k1, k2, k3. This formulation of rates may depend on cellular volume.
A few reactions represent traffic between compartments of different volumes. Where such reactions involve concentration units, please use the #/cell rate terms as the
are unambiguous.
Time units: Seconds in all cases.
Total Volume of Synapse = 0.1 femtoliters (fl)
Volume of cytosolic portion = 0.09 fl
Volume of Postsynaptic Density (PSD) = 0.01 fl

The enzyme rates are related as follows:
Km = (k2 + k3)/k1 (after conversion of units)
Kcat = k3.
Ratio = k2/k3

Initial concentrations (CoInit) are mostly zero, except for a few key molecules.
There is a flag for 'buffered' in the molecule concentration table. When this flag is zero the molecule concentrations are computed
according to the reaction equations. If the flag is one the molecule concentration is held fixed to its initial concentration.

The entire model scheme is presented as composite tables for molecules, reactions and enzymes.

All equations.
Reactions.

Reaction	kf	kb	Kf	Kb
CaMKII-PSD + CaM-Ca4-PSD <====> CaMKII-CaM-PSD	8.3333 #^-1.s^-1	0 s^-1	50 uM^-1.s^-1	0 s^-1
CaMKII-CaM + NMDAR <====> CaMKII-CaM-PSD	2e-05 #^-1.s^-1	0 s^-1	0.00108 uM^-1.s^-1	0 s^-1
CaMKII-thr286*-CaM + NMDAR <====> CaMKII-thr286-CaM-PSD	2e-05 #^-1.s^-1	0 s^-1	0.00108 uM^-1.s^-1	0 s^-1
CaMKII-PSD <====> CaMKII + NMDAR	0.3 s^-1	1e-05 #^-1.s^-1	0.3 s^-1	6e-05 uM^-1.s^-1
CaMKII-thr305-PSD <====> CaMK-thr305 + NMDAR	0.3 s^-1	1e-05 #^-1.s^-1	0.3 s^-1	6e-05 uM^-1.s^-1
CaMKII-thr286-PSD + CaM-Ca4-PSD <====> CaMKII-thr286-CaM-PSD	166.67 #^-1.s^-1	0.1 s^-1	1000 uM^-1.s^-1	0.1 s^-1
CaM-Ca4-PSD <====> CaM-Ca4	540 s^-1	60 s^-1	540 s^-1	60 s^-1
I1* + PP1-active_PSD <====> PP1-I1*	83.33 #^-1.s^-1	0.1 s^-1	499.98 uM^-1.s^-1	0.1 s^-1
CaM-Ca3 + Ca <====> CaM-Ca4	0.0086111 #^-1.s^-1	10 s^-1	0.465 uM^-1.s^-1	10 s^-1
GluR23_M <====> GluR23_I	0.00035 s^-1	0.0014 s^-1	0.00035 s^-1	0.0014 s^-1
CaMKII-CaM-PSD <====> CaM-Ca4-PSD + CaMKII-PSD	5 s^-1	0 #^-1.s^-1	5 s^-1	0 uM^-1.s^-1
PKC-control <====> PKC-active	2.5 s^-1	2.5 s^-1	2.5 s^-1	2.5 s^-1
Ca_control_cyt <====> Ca	100 s^-1	100 s^-1	100 s^-1	100 s^-1
Ca_control_PSD <====> Ca-PSD	100 s^-1	100 s^-1	100 s^-1	100 s^-1
basal_CaMKII_PSD_control <====> basal_CaMKII_PSD	1 s^-1	1 s^-1	1 s^-1	1 s^-1
AMPAR_bulk <====> A_B	0.018 s^-1	1 s^-1	0.018 s^-1	1 s^-1
CaM-Ca4 + CaMKII <====> CaMKII-CaM	0.92592 #^-1.s^-1	5 s^-1	50 uM^-1.s^-1	5 s^-1
CaMKII-thr286 + CaM-Ca4 <====> CaMKII-thr286*-CaM	18.522 #^-1.s^-1	0.1 s^-1	1000.2 uM^-1.s^-1	0.1 s^-1
CaM + 2 Ca <====> CaM-TR2-Ca2	0.024691 #^-2.s^-1	72 s^-1	71.999 uM^-2.s^-1	72 s^-1
CaM-TR2-Ca2 + Ca <====> CaM-Ca3	0.066667 #^-1.s^-1	10 s^-1	3.6 uM^-1.s^-1	10 s^-1
neurogranin + CaM <====> neurogranin-CaM	0.0055556 #^-1.s^-1	1 s^-1	0.3 uM^-1.s^-1	1 s^-1
neurogranin* <====> neurogranin	0.005 s^-1	0 s^-1	0.005 s^-1	0 s^-1
CaM-PSD + 2 Ca-PSD <====> CaM-TR2-Ca2-PSD	2 #^-2.s^-1	72 s^-1	72 uM^-2.s^-1	72 s^-1
CaM-TR2-Ca2-PSD + Ca-PSD <====> CaM-Ca3-PSD	0.6 #^-1.s^-1	10 s^-1	3.6 uM^-1.s^-1	10 s^-1
CaM-Ca3-PSD + Ca-PSD <====> CaM-Ca4-PSD	0.077502 #^-1.s^-1	10 s^-1	0.46501 uM^-1.s^-1	10 s^-1
neurogranin_PSD + CaM-PSD <====> neurogranin-CaM_PSD	0.05 #^-1.s^-1	1 s^-1	0.3 uM^-1.s^-1	1 s^-1
neurogranin*_PSD <====> neurogranin_PSD	0.005 s^-1	0 s^-1	0.005 s^-1	0 s^-1
I1* + PP1-active <====> PP1-I1*	9.2589 #^-1.s^-1	0.1 s^-1	499.98 uM^-1.s^-1	0.1 s^-1
PP1-I1 <====> PP1-active + I1	1 s^-1	0 #^-1.s^-1	1 s^-1	0 uM^-1.s^-1
2 Ca + CaNAB-Ca2 <====> CaNAB-Ca4	0.0012346 #^-2.s^-1	1 s^-1	3.6001 uM^-2.s^-1	1 s^-1
CaNAB + 2 Ca <====> CaNAB-Ca2	3.4321 #^-2.s^-1	1 s^-1	10008 uM^-2.s^-1	1 s^-1
CaM-Ca4 + CaNAB-Ca4 <====> CaM_Ca_n-CaNAB	11.111 #^-1.s^-1	1 s^-1	599.99 uM^-1.s^-1	1 s^-1
R2C2 + cAMP <====> R2C2-cAMP	1 #^-1.s^-1	33 s^-1	54 uM^-1.s^-1	33 s^-1
R2C2-cAMP + cAMP <====> R2C2-cAMP2	1 #^-1.s^-1	33 s^-1	54 uM^-1.s^-1	33 s^-1
R2C2-cAMP2 + cAMP <====> R2C2-cAMP3	1.3889 #^-1.s^-1	110 s^-1	75.001 uM^-1.s^-1	110 s^-1
cAMP + R2C2-cAMP3 <====> R2C2-cAMP4	1.3889 #^-1.s^-1	32.5 s^-1	75.001 uM^-1.s^-1	32.5 s^-1
R2C2-cAMP4 <====> PKA-active + R2C-cAMP4	60 s^-1	0.33333 #^-1.s^-1	60 s^-1	18 uM^-1.s^-1
R2C-cAMP4 <====> PKA-active + R2-cAMP4	60 s^-1	0.33333 #^-1.s^-1	60 s^-1	18 uM^-1.s^-1
PKA-active + PKA-inhibitor <====> inhibited-PKA	1.1111 #^-1.s^-1	1 s^-1	59.999 uM^-1.s^-1	1 s^-1
CaM-Ca4 + AC1 <====> AC1-CaM	0.92592 #^-1.s^-1	1 s^-1	50 uM^-1.s^-1	1 s^-1
AC2* <====> AC2	0.1 s^-1	0 s^-1	0.1 s^-1	0 s^-1
cAMP-PDE* <====> cAMP-PDE	0.01 s^-1	0 s^-1	0.01 s^-1	0 s^-1
PDE1 + CaM-Ca4 <====> CaM.PDE1	13.333 #^-1.s^-1	5 s^-1	719.98 uM^-1.s^-1	5 s^-1
cAMP <====> cAMP_in_dend	300 s^-1	5.4 s^-1	300 s^-1	5.4 s^-1
PP1-I1 <====> I1 + PP1-active_PSD	1 s^-1	0 #^-1.s^-1	1 s^-1	0 uM^-1.s^-1
A831*_B831* <====> A831*_B831* + Anchor	0.0008 s^-1	0 #^-1.s^-1	0.0008 s^-1	0 uM^-1.s^-1
A_B831* <====> A_B831* + Anchor	0.0008 s^-1	0 #^-1.s^-1	0.0008 s^-1	0 uM^-1.s^-1
A831*_B <====> A831*_B + Anchor	0.0008 s^-1	0 #^-1.s^-1	0.0008 s^-1	0 uM^-1.s^-1
A_B <====> A_B + Anchor	0.0008 s^-1	0 #^-1.s^-1	0.0008 s^-1	0 uM^-1.s^-1
A835*845*_B835*845* <====> AMPAR_deg	3.6e-05 s^-1	0 s^-1	3.6e-05 s^-1	0 s^-1
A845*_B831*845* <====> AMPAR_deg	3.6e-05 s^-1	0 s^-1	3.6e-05 s^-1	0 s^-1
A831*845*_B845* <====> AMPAR_deg	3.6e-05 s^-1	0 s^-1	3.6e-05 s^-1	0 s^-1
A845*_B845* <====> AMPAR_deg	3.6e-05 s^-1	0 s^-1	3.6e-05 s^-1	0 s^-1
A835*845*_B835*845* + Anchor <====> A835*845*_B835*845*	0.0002 #^-1.s^-1	0.008 s^-1	0.0108 uM^-1.s^-1	0.008 s^-1
A845*_B831*845* + Anchor <====> A845*_B831*845*	0.0002 #^-1.s^-1	0.008 s^-1	0.0108 uM^-1.s^-1	0.008 s^-1
A831*845*_B845* + Anchor <====> A831*845*_B845*	0.0002 #^-1.s^-1	0.008 s^-1	0.0108 uM^-1.s^-1	0.008 s^-1
A845*_B845* + Anchor <====> A845*_B845*	0.0002 #^-1.s^-1	0.008 s^-1	0.0108 uM^-1.s^-1	0.008 s^-1

Enzymes for group /##[]

Enzyme-reaction	k1	k2	k3	Km	kcat	ratio
CaMKII-thr286*-CaM ---PP1-active--> CaMKII-CaM	0.045397 #^-1.s^-1	10 s^-1	2.5 s^-1	5.099 uM	2.5 s^-1	4
CaMKII-thr286 ---PP1-active--> CaMKII	0.045397 #^-1.s^-1	10 s^-1	2.5 s^-1	5.099 uM	2.5 s^-1	4
CaMKII*** ---PP1-active--> CaMKII-thr286	0.045397 #^-1.s^-1	10 s^-1	2.5 s^-1	5.099 uM	2.5 s^-1	4
CaMKII*** ---PP1-active--> CaMK-thr305	0.045397 #^-1.s^-1	10 s^-1	2.5 s^-1	5.099 uM	2.5 s^-1	4
CaMK-thr305 ---PP1-active--> CaMKII	0.045397 #^-1.s^-1	10 s^-1	2.5 s^-1	5.099 uM	2.5 s^-1	4
cAMP-PDE ---PKA-active--> cAMP-PDE*	0.11111 #^-1.s^-1	36 s^-1	9 s^-1	7.5001 uM	9 s^-1	4
I1 ---PKA-active--> I1*	0.11111 #^-1.s^-1	36 s^-1	9 s^-1	7.5001 uM	9 s^-1	4
I1 ---PKA-active--> I1*	0.11111 #^-1.s^-1	36 s^-1	9 s^-1	7.5001 uM	9 s^-1	4
A_B ---PKA-active--> A845*_B	0.074072 #^-1.s^-1	24 s^-1	6 s^-1	7.5002 uM	6 s^-1	4
A831*_B ---PKA-active--> A831*845*_B	0.074072 #^-1.s^-1	24 s^-1	6 s^-1	7.5002 uM	6 s^-1	4
A_B831* ---PKA-active--> A845*_B831*	0.074072 #^-1.s^-1	24 s^-1	6 s^-1	7.5002 uM	6 s^-1	4
A831*_B831* ---PKA-active--> A831*845*_B831*	0.074072 #^-1.s^-1	24 s^-1	6 s^-1	7.5002 uM	6 s^-1	4
A_B ---PKA-active--> A_B845*	0.074072 #^-1.s^-1	24 s^-1	6 s^-1	7.5002 uM	6 s^-1	4
A831*_B ---PKA-active--> AA831*_B845*	0.074072 #^-1.s^-1	24 s^-1	6 s^-1	7.5002 uM	6 s^-1	4
A_B831* ---PKA-active--> A_B831*845*	0.074072 #^-1.s^-1	24 s^-1	6 s^-1	7.5002 uM	6 s^-1	4
A831*_B831* ---PKA-active--> A831*_B831*845*	0.074072 #^-1.s^-1	24 s^-1	6 s^-1	7.5002 uM	6 s^-1	4
A_B845* ---PKA-active--> A845*_B845*	0.074072 #^-1.s^-1	24 s^-1	6 s^-1	7.5002 uM	6 s^-1	4
AA831*_B845* ---PKA-active--> A831*845*_B845*	0.074072 #^-1.s^-1	24 s^-1	6 s^-1	7.5002 uM	6 s^-1	4
A_B831*845* ---PKA-active--> A845*_B831*845*	0.074072 #^-1.s^-1	24 s^-1	6 s^-1	7.5002 uM	6 s^-1	4
A831*_B831*845* ---PKA-active--> A835*845*_B835*845*	0.074072 #^-1.s^-1	24 s^-1	6 s^-1	7.5002 uM	6 s^-1	4
A845*_B ---PKA-active--> A845*_B845*	0.074072 #^-1.s^-1	24 s^-1	6 s^-1	7.5002 uM	6 s^-1	4
A831*845*_B ---PKA-active--> A831*845*_B845*	0.074072 #^-1.s^-1	24 s^-1	6 s^-1	7.5002 uM	6 s^-1	4
A845*_B831* ---PKA-active--> A845*_B831*845*	0.074072 #^-1.s^-1	24 s^-1	6 s^-1	7.5002 uM	6 s^-1	4
A831*845*_B831* ---PKA-active--> A835*845*_B835*845*	0.074072 #^-1.s^-1	24 s^-1	6 s^-1	7.5002 uM	6 s^-1	4
A_B845* ---PKA-active--> A845*_B845*	0.074072 #^-1.s^-1	24 s^-1	6 s^-1	7.5002 uM	6 s^-1	4
A845*_B ---PKA-active--> A845*_B845*	0.074072 #^-1.s^-1	24 s^-1	6 s^-1	7.5002 uM	6 s^-1	4
A_B ---PKA-active--> A845*_B	0.074072 #^-1.s^-1	24 s^-1	6 s^-1	7.5002 uM	6 s^-1	4
A_B ---PKA-active--> A_B845*	0.074072 #^-1.s^-1	24 s^-1	6 s^-1	7.5002 uM	6 s^-1	4
A831*_B845* ---PKA-active--> A831*845*_B845*	0.074072 #^-1.s^-1	24 s^-1	6 s^-1	7.5002 uM	6 s^-1	4
A831*845*_B ---PKA-active--> A831*845*_B845*	0.074072 #^-1.s^-1	24 s^-1	6 s^-1	7.5002 uM	6 s^-1	4
A831*_B ---PKA-active--> A831*_B845*	0.074072 #^-1.s^-1	24 s^-1	6 s^-1	7.5002 uM	6 s^-1	4
A831*_B ---PKA-active--> A831*845*_B	0.074072 #^-1.s^-1	24 s^-1	6 s^-1	7.5002 uM	6 s^-1	4
A_B831*845* ---PKA-active--> A845*_B831*845*	0.074072 #^-1.s^-1	24 s^-1	6 s^-1	7.5002 uM	6 s^-1	4
A845*_B831* ---PKA-active--> A845*_B831*845*	0.074072 #^-1.s^-1	24 s^-1	6 s^-1	7.5002 uM	6 s^-1	4
A_B831* ---PKA-active--> A_B831*845*	0.074072 #^-1.s^-1	24 s^-1	6 s^-1	7.5002 uM	6 s^-1	4
A_B831* ---PKA-active--> A845*_B831*	0.074072 #^-1.s^-1	24 s^-1	6 s^-1	7.5002 uM	6 s^-1	4
A831*_B831*845* ---PKA-active--> A835*845*_B835*845*	0.074072 #^-1.s^-1	24 s^-1	6 s^-1	7.5002 uM	6 s^-1	4
A831*845*_B831* ---PKA-active--> A835*845*_B835*845*	0.074072 #^-1.s^-1	24 s^-1	6 s^-1	7.5002 uM	6 s^-1	4
A831*_B831* ---PKA-active--> A831*_B831*845*	0.074072 #^-1.s^-1	24 s^-1	6 s^-1	7.5002 uM	6 s^-1	4
A831*_B831* ---PKA-active--> A831*845*_B831*	0.074072 #^-1.s^-1	24 s^-1	6 s^-1	7.5002 uM	6 s^-1	4
I1* ---PP2A--> I1	0.01196 #^-1.s^-1	8.3334 s^-1	2 s^-1	16 uM	2 s^-1	4.1667
PP1-I1* ---PP2A--> PP1-I1	0.01196 #^-1.s^-1	8.3334 s^-1	2 s^-1	16 uM	2 s^-1	4.1667
I1* ---PP2A--> I1	0.01196 #^-1.s^-1	8.3334 s^-1	2 s^-1	16 uM	2 s^-1	4.1667
PP1-I1* ---PP2A--> PP1-I1	0.01196 #^-1.s^-1	8.3334 s^-1	2 s^-1	16 uM	2 s^-1	4.1667
I1* ---CaNAB-Ca4--> I1	0.00063333 #^-1.s^-1	0.136 s^-1	0.034 s^-1	4.9708 uM	0.034 s^-1	4
I1* ---CaNAB-Ca4--> I1	0.00063334 #^-1.s^-1	0.136 s^-1	0.034 s^-1	4.9707 uM	0.034 s^-1	4
CaMKII-thr286-PSD ---tot-auto-PSD--> CaMKII***-PSD	0.01 #^-1.s^-1	24 s^-1	6 s^-1	500 uM	6 s^-1	4
CaMKII-CaM-PSD ---tot-auto-PSD--> CaMKII-thr286-CaM-PSD	0.00083333 #^-1.s^-1	2 s^-1	0.5 s^-1	500 uM	0.5 s^-1	4
CaMKII-PSD ---tot-auto-PSD--> CaMKII-thr286-PSD	0.0033333 #^-1.s^-1	8 s^-1	2 s^-1	500.01 uM	2 s^-1	4
A845*_B845* ---actCaMKII-PSD--> A831*845*_B845*	0.0046296 #^-1.s^-1	2 s^-1	0.5 s^-1	90.001 uM	0.5 s^-1	4
A_B845* ---actCaMKII-PSD--> A831*_B845*	0.0046296 #^-1.s^-1	2 s^-1	0.5 s^-1	90.001 uM	0.5 s^-1	4
A845*_B ---actCaMKII-PSD--> A831*845*_B	0.0046296 #^-1.s^-1	2 s^-1	0.5 s^-1	90.001 uM	0.5 s^-1	4
A_B ---actCaMKII-PSD--> A831*_B	0.0046296 #^-1.s^-1	2 s^-1	0.5 s^-1	90.001 uM	0.5 s^-1	4
A845*_B845* ---actCaMKII-PSD--> A845*_B831*845*	0.0046296 #^-1.s^-1	2 s^-1	0.5 s^-1	90.001 uM	0.5 s^-1	4
A_B845* ---actCaMKII-PSD--> A_B831*845*	0.0046296 #^-1.s^-1	2 s^-1	0.5 s^-1	90.001 uM	0.5 s^-1	4
A845*_B ---actCaMKII-PSD--> A845*_B831*	0.0046296 #^-1.s^-1	2 s^-1	0.5 s^-1	90.001 uM	0.5 s^-1	4
A_B ---actCaMKII-PSD--> A_B831*	0.0046296 #^-1.s^-1	2 s^-1	0.5 s^-1	90.001 uM	0.5 s^-1	4
A845*_B831*845* ---actCaMKII-PSD--> A835*845*_B835*845*	0.0046296 #^-1.s^-1	2 s^-1	0.5 s^-1	90.001 uM	0.5 s^-1	4
A_B831*845* ---actCaMKII-PSD--> A831*_B831*845*	0.0046296 #^-1.s^-1	2 s^-1	0.5 s^-1	90.001 uM	0.5 s^-1	4
A845*_B831* ---actCaMKII-PSD--> A831*845*_B831*	0.0046296 #^-1.s^-1	2 s^-1	0.5 s^-1	90.001 uM	0.5 s^-1	4
A_B831* ---actCaMKII-PSD--> A831*_B831*	0.0046296 #^-1.s^-1	2 s^-1	0.5 s^-1	90.001 uM	0.5 s^-1	4
A831*845*_B845* ---actCaMKII-PSD--> A835*845*_B835*845*	0.0046296 #^-1.s^-1	2 s^-1	0.5 s^-1	90.001 uM	0.5 s^-1	4
A831*_B845* ---actCaMKII-PSD--> A831*_B831*845*	0.0046296 #^-1.s^-1	2 s^-1	0.5 s^-1	90.001 uM	0.5 s^-1	4
A831*845*_B ---actCaMKII-PSD--> A831*845*_B831*	0.0046296 #^-1.s^-1	2 s^-1	0.5 s^-1	90.001 uM	0.5 s^-1	4
A831*_B ---actCaMKII-PSD--> A831*_B831*	0.0046296 #^-1.s^-1	2 s^-1	0.5 s^-1	90.001 uM	0.5 s^-1	4
CaMKII-thr286-CaM-PSD ---PP1-active_PSD--> CaMKII-CaM-PSD	0.083333 #^-1.s^-1	0.8 s^-1	0.2 s^-1	2 uM	0.2 s^-1	4
CaMKII-thr286-PSD ---PP1-active_PSD--> CaMKII-PSD	0.083333 #^-1.s^-1	0.8 s^-1	0.2 s^-1	2 uM	0.2 s^-1	4
CaMKII***-PSD ---PP1-active_PSD--> CaMKII-thr286-PSD	0.083333 #^-1.s^-1	0.8 s^-1	0.2 s^-1	2 uM	0.2 s^-1	4
CaMKII***-PSD ---PP1-active_PSD--> CaMKII-thr305-PSD	0.083333 #^-1.s^-1	0.8 s^-1	0.2 s^-1	2 uM	0.2 s^-1	4
A845*_B845* ---PP1-active_PSD--> A_B845*	0.14583 #^-1.s^-1	0.68 s^-1	0.17 s^-1	0.97145 uM	0.17 s^-1	4
A845*_B845* ---PP1-active_PSD--> A845*_B	0.14583 #^-1.s^-1	0.68 s^-1	0.17 s^-1	0.97145 uM	0.17 s^-1	4
A845*_B ---PP1-active_PSD--> A_B	0.14583 #^-1.s^-1	0.68 s^-1	0.17 s^-1	0.97145 uM	0.17 s^-1	4
A_B845* ---PP1-active_PSD--> A_B	0.14583 #^-1.s^-1	0.68 s^-1	0.17 s^-1	0.97145 uM	0.17 s^-1	4
A831*845*_B845* ---PP1-active_PSD--> A831*_B845*	0.14583 #^-1.s^-1	0.68 s^-1	0.17 s^-1	0.97145 uM	0.17 s^-1	4
A831*845*_B845* ---PP1-active_PSD--> A831*845*_B	0.14583 #^-1.s^-1	0.68 s^-1	0.17 s^-1	0.97145 uM	0.17 s^-1	4
A831*_B845* ---PP1-active_PSD--> A831*_B	0.14583 #^-1.s^-1	0.68 s^-1	0.17 s^-1	0.97145 uM	0.17 s^-1	4
A831*845*_B ---PP1-active_PSD--> A831*_B	0.14583 #^-1.s^-1	0.68 s^-1	0.17 s^-1	0.97145 uM	0.17 s^-1	4
A831*845*_B845* ---PP1-active_PSD--> A845*_B845*	0.14583 #^-1.s^-1	1.4 s^-1	0.35 s^-1	2 uM	0.35 s^-1	4
A831*_B845* ---PP1-active_PSD--> A_B845*	0.14583 #^-1.s^-1	1.4 s^-1	0.35 s^-1	2 uM	0.35 s^-1	4
A831*845*_B ---PP1-active_PSD--> A845*_B	0.14583 #^-1.s^-1	1.4 s^-1	0.35 s^-1	2 uM	0.35 s^-1	4
A831*_B ---PP1-active_PSD--> A_B	0.14583 #^-1.s^-1	1.4 s^-1	0.35 s^-1	2 uM	0.35 s^-1	4
A845*_B831*845* ---PP1-active_PSD--> A_B831*845*	0.14583 #^-1.s^-1	0.68 s^-1	0.17 s^-1	0.97145 uM	0.17 s^-1	4
A845*_B831*845* ---PP1-active_PSD--> A845*_B831*	0.14583 #^-1.s^-1	0.68 s^-1	0.17 s^-1	0.97145 uM	0.17 s^-1	4
A_B831*845* ---PP1-active_PSD--> A_B831*	0.14583 #^-1.s^-1	0.68 s^-1	0.17 s^-1	0.97145 uM	0.17 s^-1	4
A845*_B831* ---PP1-active_PSD--> A_B831*	0.14583 #^-1.s^-1	0.68 s^-1	0.17 s^-1	0.97145 uM	0.17 s^-1	4
A835*845*_B835*845* ---PP1-active_PSD--> A831*_B831*845*	0.14583 #^-1.s^-1	0.68 s^-1	0.17 s^-1	0.97145 uM	0.17 s^-1	4
A835*845*_B835*845* ---PP1-active_PSD--> A831*845*_B831*	0.14583 #^-1.s^-1	0.68 s^-1	0.17 s^-1	0.97145 uM	0.17 s^-1	4
A831*_B831*845* ---PP1-active_PSD--> A831*_B831*	0.14583 #^-1.s^-1	0.68 s^-1	0.17 s^-1	0.97145 uM	0.17 s^-1	4
A831*845*_B831* ---PP1-active_PSD--> A831*_B831*	0.14583 #^-1.s^-1	0.68 s^-1	0.17 s^-1	0.97145 uM	0.17 s^-1	4
A_B831*845* ---PP1-active_PSD--> A_B845*	0.14583 #^-1.s^-1	1.4 s^-1	0.35 s^-1	2 uM	0.35 s^-1	4

A845*_B831*845* ---PP1-active_PSD--> A845*_B845*	0.14583 #^-1.s^-1	1.4 s^-1	0.35 s^-1	2 uM	0.35 s^-1	4
A845*_B831* ---PP1-active_PSD--> A845*_B	0.14583 #^-1.s^-1	1.4 s^-1	0.35 s^-1	2 uM	0.35 s^-1	4
A_B831* ---PP1-active_PSD--> A_B	0.14583 #^-1.s^-1	1.4 s^-1	0.35 s^-1	2 uM	0.35 s^-1	4
A835*845*_B835*845* ---PP1-active_PSD--> A845*_B831*845*	0.14583 #^-1.s^-1	1.4 s^-1	0.35 s^-1	2 uM	0.35 s^-1	4
A831*_B831*845* ---PP1-active_PSD--> A_B831*845*	0.14583 #^-1.s^-1	1.4 s^-1	0.35 s^-1	2 uM	0.35 s^-1	4
A831*845*_B831* ---PP1-active_PSD--> A845*_B831*	0.14583 #^-1.s^-1	1.4 s^-1	0.35 s^-1	2 uM	0.35 s^-1	4
A831*_B831* ---PP1-active_PSD--> A_B831*	0.14583 #^-1.s^-1	1.4 s^-1	0.35 s^-1	2 uM	0.35 s^-1	4
A835*845*_B835*845* ---PP1-active_PSD--> A831*845*_B845*	0.14583 #^-1.s^-1	1.4 s^-1	0.35 s^-1	2 uM	0.35 s^-1	4
A831*_B831*845* ---PP1-active_PSD--> A831*_B845*	0.14583 #^-1.s^-1	1.4 s^-1	0.35 s^-1	2 uM	0.35 s^-1	4
A831*845*_B831* ---PP1-active_PSD--> A831*845*_B	0.14583 #^-1.s^-1	1.4 s^-1	0.35 s^-1	2 uM	0.35 s^-1	4
A831*_B831* ---PP1-active_PSD--> A831*_B	0.14583 #^-1.s^-1	1.4 s^-1	0.35 s^-1	2 uM	0.35 s^-1	4
CaMKII-thr305-PSD ---PP1-active_PSD--> CaMKII-PSD	0.083333 #^-1.s^-1	0.8 s^-1	0.2 s^-1	2 uM	0.2 s^-1	4
neurogranin ---PKC-active--> neurogranin*	0.0018889 #^-1.s^-1	2.34 s^-1	0.58 s^-1	28.627 uM	0.58 s^-1	4.0345
neurogranin-CaM ---PKC-active--> CaM + neurogranin*	0.0011333 #^-1.s^-1	1.4 s^-1	0.35 s^-1	28.596 uM	0.35 s^-1	4
AC2 ---PKC-active--> AC2*	0.011111 #^-1.s^-1	16 s^-1	4 s^-1	33.334 uM	4 s^-1	4
neurogranin_PSD ---PKC-active--> neurogranin*_PSD	0.0018889 #^-1.s^-1	2.34 s^-1	0.58 s^-1	28.627 uM	0.58 s^-1	4.0345
neurogranin-CaM_PSD ---PKC-active--> CaM-PSD + neurogranin*_PSD	0.0011333 #^-1.s^-1	1.4 s^-1	0.35 s^-1	28.596 uM	0.35 s^-1	4
neurogranin* ---CaM_Ca_n-CaNAB--> neurogranin	0.0061778 #^-1.s^-1	2.67 s^-1	0.67 s^-1	10.012 uM	0.67 s^-1	3.9851
I1* ---CaM_Ca_n-CaNAB--> I1	0.0063333 #^-1.s^-1	1.36 s^-1	0.34 s^-1	4.9708 uM	0.34 s^-1	4
PP1-I1* ---CaM_Ca_n-CaNAB--> PP1-I1	0.0063333 #^-1.s^-1	1.36 s^-1	0.34 s^-1	4.9708 uM	0.34 s^-1	4
I1* ---CaM_Ca_n-CaNAB--> I1	0.0063334 #^-1.s^-1	1.36 s^-1	0.34 s^-1	4.9707 uM	0.34 s^-1	4
PP1-I1* ---CaM_Ca_n-CaNAB--> PP1-I1	0.0063334 #^-1.s^-1	1.36 s^-1	0.34 s^-1	4.9707 uM	0.34 s^-1	4
A845*_B ---CaM_Ca_n-CaNAB--> A_B	0.037256 #^-1.s^-1	8 s^-1	2 s^-1	4.9706 uM	2 s^-1	4
A831*845*_B ---CaM_Ca_n-CaNAB--> A831*_B	0.037256 #^-1.s^-1	8 s^-1	2 s^-1	4.9706 uM	2 s^-1	4
A845*_B831* ---CaM_Ca_n-CaNAB--> A_B831*	0.037256 #^-1.s^-1	8 s^-1	2 s^-1	4.9706 uM	2 s^-1	4
A831*845*_B831* ---CaM_Ca_n-CaNAB--> A831*_B831*	0.037256 #^-1.s^-1	8 s^-1	2 s^-1	4.9706 uM	2 s^-1	4
A_B845* ---CaM_Ca_n-CaNAB--> A_B	0.037256 #^-1.s^-1	8 s^-1	2 s^-1	4.9706 uM	2 s^-1	4
AA831*_B845* ---CaM_Ca_n-CaNAB--> A831*_B	0.037256 #^-1.s^-1	8 s^-1	2 s^-1	4.9706 uM	2 s^-1	4
A_B831*845* ---CaM_Ca_n-CaNAB--> A_B831*	0.037256 #^-1.s^-1	8 s^-1	2 s^-1	4.9706 uM	2 s^-1	4
A831*_B831*845* ---CaM_Ca_n-CaNAB--> A831*_B831*	0.037256 #^-1.s^-1	8 s^-1	2 s^-1	4.9706 uM	2 s^-1	4
A845*_B845* ---CaM_Ca_n-CaNAB--> A_B845*	0.037256 #^-1.s^-1	8 s^-1	2 s^-1	4.9706 uM	2 s^-1	4
A831*845*_B845* ---CaM_Ca_n-CaNAB--> AA831*_B845*	0.037256 #^-1.s^-1	8 s^-1	2 s^-1	4.9706 uM	2 s^-1	4
A845*_B831*845* ---CaM_Ca_n-CaNAB--> A_B831*845*	0.037256 #^-1.s^-1	8 s^-1	2 s^-1	4.9706 uM	2 s^-1	4
A835*845*_B835*845* ---CaM_Ca_n-CaNAB--> A831*_B831*845*	0.037256 #^-1.s^-1	8 s^-1	2 s^-1	4.9706 uM	2 s^-1	4
A845*_B845* ---CaM_Ca_n-CaNAB--> A845*_B	0.037256 #^-1.s^-1	8 s^-1	2 s^-1	4.9706 uM	2 s^-1	4
A831*845*_B845* ---CaM_Ca_n-CaNAB--> A831*845*_B	0.037256 #^-1.s^-1	8 s^-1	2 s^-1	4.9706 uM	2 s^-1	4
A845*_B831*845* ---CaM_Ca_n-CaNAB--> A845*_B831*	0.037256 #^-1.s^-1	8 s^-1	2 s^-1	4.9706 uM	2 s^-1	4
A835*845*_B835*845* ---CaM_Ca_n-CaNAB--> A831*845*_B831*	0.037256 #^-1.s^-1	8 s^-1	2 s^-1	4.9706 uM	2 s^-1	4
A845*_B845* ---CaM_Ca_n-CaNAB--> A_B845*	0.037256 #^-1.s^-1	8 s^-1	2 s^-1	4.9706 uM	2 s^-1	4
A845*_B845* ---CaM_Ca_n-CaNAB--> A845*_B	0.037256 #^-1.s^-1	8 s^-1	2 s^-1	4.9706 uM	2 s^-1	4
A_B845* ---CaM_Ca_n-CaNAB--> A_B	0.037256 #^-1.s^-1	8 s^-1	2 s^-1	4.9706 uM	2 s^-1	4
A845*_B ---CaM_Ca_n-CaNAB--> A_B	0.037256 #^-1.s^-1	8 s^-1	2 s^-1	4.9706 uM	2 s^-1	4
A831*845*_B845* ---CaM_Ca_n-CaNAB--> A831*_B845*	0.037256 #^-1.s^-1	8 s^-1	2 s^-1	4.9706 uM	2 s^-1	4
A831*845*_B845* ---CaM_Ca_n-CaNAB--> A831*845*_B	0.037256 #^-1.s^-1	8 s^-1	2 s^-1	4.9706 uM	2 s^-1	4
A831*_B845* ---CaM_Ca_n-CaNAB--> A831*_B	0.037256 #^-1.s^-1	8 s^-1	2 s^-1	4.9706 uM	2 s^-1	4
A831*845*_B ---CaM_Ca_n-CaNAB--> A831*_B	0.037256 #^-1.s^-1	8 s^-1	2 s^-1	4.9706 uM	2 s^-1	4
A845*_B831*845* ---CaM_Ca_n-CaNAB--> A_B831*845*	0.037256 #^-1.s^-1	8 s^-1	2 s^-1	4.9706 uM	2 s^-1	4
A845*_B831*845* ---CaM_Ca_n-CaNAB--> A845*_B831*	0.037256 #^-1.s^-1	8 s^-1	2 s^-1	4.9706 uM	2 s^-1	4
A_B831*845* ---CaM_Ca_n-CaNAB--> A_B831*	0.037256 #^-1.s^-1	8 s^-1	2 s^-1	4.9706 uM	2 s^-1	4
A845*_B831* ---CaM_Ca_n-CaNAB--> A_B831*	0.037256 #^-1.s^-1	8 s^-1	2 s^-1	4.9706 uM	2 s^-1	4
A835*845*_B835*845* ---CaM_Ca_n-CaNAB--> A831*_B831*845*	0.037256 #^-1.s^-1	8 s^-1	2 s^-1	4.9706 uM	2 s^-1	4
A835*845*_B835*845* ---CaM_Ca_n-CaNAB--> A831*845*_B831*	0.037256 #^-1.s^-1	8 s^-1	2 s^-1	4.9706 uM	2 s^-1	4
A831*845*_B831* ---CaM_Ca_n-CaNAB--> A831*_B831*	0.037256 #^-1.s^-1	8 s^-1	2 s^-1	4.9706 uM	2 s^-1	4
A831*_B831*845* ---CaM_Ca_n-CaNAB--> A831*_B831*	0.037256 #^-1.s^-1	8 s^-1	2 s^-1	4.9706 uM	2 s^-1	4
neurogranin*_PSD ---CaM_Ca_n-CaNAB--> neurogranin_PSD	0.0061778 #^-1.s^-1	2.67 s^-1	0.67 s^-1	10.012 uM	0.67 s^-1	3.9851
CaMKII-thr286-PSD ---tot-CaM-CaMKII-PSD--> CaMKII***-PSD	0.015625 #^-1.s^-1	24 s^-1	6 s^-1	320 uM	6 s^-1	4
CaMKII-CaM-PSD ---tot-CaM-CaMKII-PSD--> CaMKII-thr286-CaM-PSD	0.0013021 #^-1.s^-1	2 s^-1	0.5 s^-1	320 uM	0.5 s^-1	4
CaMKII-PSD ---tot-CaM-CaMKII-PSD--> CaMKII-thr286-PSD	0.0052083 #^-1.s^-1	8 s^-1	2 s^-1	320 uM	2 s^-1	4
CaMKII-thr286 ---tot_CaM_CaMKII--> CaMKII***	0.0024474 #^-1.s^-1	24 s^-1	6 s^-1	227 uM	6 s^-1	4
CaMKII-CaM ---tot_CaM_CaMKII--> CaMKII-thr286*-CaM	0.00020395 #^-1.s^-1	2 s^-1	0.5 s^-1	227 uM	0.5 s^-1	4
CaMKII-thr286 ---tot_autonomous_CaMKII--> CaMKII***	0.0015873 #^-1.s^-1	24 s^-1	6 s^-1	350 uM	6 s^-1	4
CaMKII-CaM ---tot_autonomous_CaMKII--> CaMKII-thr286*-CaM	0.00013228 #^-1.s^-1	2 s^-1	0.5 s^-1	349.99 uM	0.5 s^-1	4
ATP ---AC1-CaM--> cAMP	0.0013889 #^-1.s^-1	18 s^-1	4.5 s^-1	300 uM	4.5 s^-1	4
ATP ---AC2*--> cAMP	0.00061728 #^-1.s^-1	8 s^-1	2 s^-1	300 uM	2 s^-1	4
cAMP ---cAMP-PDE--> AMP	0.046667 #^-1.s^-1	40 s^-1	10 s^-1	19.841 uM	10 s^-1	4
cAMP ---cAMP-PDE*--> AMP	0.093333 #^-1.s^-1	80 s^-1	20 s^-1	19.841 uM	20 s^-1	4
cAMP ---PDE1--> AMP	0.0038889 #^-1.s^-1	6.67 s^-1	1.667 s^-1	39.7 uM	1.667 s^-1	4.0012
cAMP ---CaM.PDE1--> AMP	0.023333 #^-1.s^-1	40 s^-1	10 s^-1	39.683 uM	10 s^-1	4

Pools for group /##[]

name	InitialConc	buffered	Volume
CaM-Ca4	0 uM	0	0.09 fl
PP1-active	1.8 uM	0	0.09 fl
cAMP	0 uM	0	0.09 fl
Ca	0.08 uM	0	0.09 fl
PKA-active	0 uM	0	0.09 fl
CaM-Ca3	0 uM	0	0.09 fl
CaM-TR2-Ca2	0 uM	0	0.09 fl
PP2A	0.11111 uM	0	0.09 fl
CaNAB-Ca4	0 uM	0	0.09 fl
CaMKII-thr286-CaM-PSD	0 uM	0	0.01 fl
CaMKII-CaM-PSD	0 uM	0	0.01 fl
CaMKII-thr286-PSD	0 uM	0	0.01 fl
CaMKII-PSD	0 uM	0	0.01 fl
CaMKII***-PSD	0 uM	0	0.01 fl
tot-auto-PSD	2 uM	0	0.01 fl
CaM-TR2-Ca2-PSD	0 uM	0	0.01 fl
CaM-Ca3-PSD	0.0025458 uM	0	0.01 fl
CaM-Ca4-PSD	0 uM	0	0.01 fl
Ca-PSD	0.08 uM	0	0.01 fl
286P-PSD	0 uM	0	0.01 fl
actCaMKII-PSD	2 uM	0	0.01 fl
tot_CaMKII_PSD	2 uM	0	0.01 fl
tot_CaMKII_cyt	22 uM	0	0.09 fl
PP1-active_PSD	8 uM	0	0.01 fl
PKC-active	0.1 uM	0	0.09 fl
temp-PIP2	2.5 uM	1	0.09 fl
I_845-P	0 uM	0	0.09 fl
tot_I_GluR12	0 uM	0	0.09 fl
total_Int	0.096296 uM	0	0.09 fl
Ser845	0 uM	0	0.01 fl
Ser845-P	0 uM	0	0.01 fl
Ser845-PP	0 uM	0	0.01 fl
Ser831	0 uM	0	0.01 fl
Ser831-P	0 uM	0	0.01 fl
Ser831-PP	0 uM	0	0.01 fl
tot_mem_GluR12	0 uM	0	0.01 fl
act_CaMKII_cyt	2 uM	0	0.09 fl
NMDAR	120 uM	0	0.01 fl
CaM_Ca_n-CaNAB	0 uM	0	0.09 fl
basal_CaMKII_cyt	2 uM	1	0.09 fl
basal_CaMKII_PSD	2 uM	0	0.01 fl
PKC-control	0.1 uM	1	0.09 fl
Ca_control_cyt	0.08 uM	1	0.09 fl
Ca_control_PSD	0.08 uM	1	0.01 fl
basal_CaMKII_PSD_control	2 uM	1	0.01 fl
Anchor	27.333 uM	0	0.01 fl
I_845	0 uM	0	0.09 fl
I_845_PP	0 uM	0	0.09 fl
tot-CaM-CaMKII-PSD	0 uM	0	0.01 fl
CaMKII-thr305-PSD	0 uM	0	0.01 fl
AMPAR_bulk	0.0092593 uM	1	5 fl
CaMKII	20 uM	0	0.09 fl
CaMKII-CaM	0 uM	0	0.09 fl
CaMKII-thr286*-CaM	0 uM	0	0.09 fl
CaMKII***	0 uM	0	0.09 fl
CaMKII-thr286	0 uM	0	0.09 fl
tot_CaM_CaMKII	0 uM	0	0.09 fl
tot_autonomous_CaMKII	2 uM	0	0.09 fl
CaMK-thr305	0 uM	0	0.09 fl
CaM	26.333 uM	0	0.09 fl
neurogranin-CaM	0 uM	0	0.09 fl
neurogranin*	0 uM	0	0.09 fl
neurogranin	10 uM	0	0.09 fl
CaM-PSD	26.333 uM	0	0.01 fl
neurogranin-CaM_PSD	0 uM	0	0.01 fl
neurogranin_PSD	10 uM	0	0.01 fl
neurogranin*_PSD	0 uM	0	0.01 fl
I1	1.8 uM	0	0.09 fl
I1*	0 uM	0	0.09 fl
PP1-I1*	0 uM	0	0.09 fl
PP1-I1	0 uM	0	0.09 fl
CaNAB	1 uM	0	0.09 fl
CaNAB-Ca2	0 uM	0	0.09 fl
R2C2	0.5 uM	0	0.09 fl
R2C2-cAMP	0 uM	0	0.09 fl
R2C2-cAMP2	0 uM	0	0.09 fl
R2C2-cAMP3	0 uM	0	0.09 fl
R2C2-cAMP4	0 uM	0	0.09 fl
R2C-cAMP4	0 uM	0	0.09 fl
R2-cAMP4	0 uM	0	0.09 fl
PKA-inhibitor	0.25926 uM	0	0.09 fl
inhibited-PKA	0 uM	0	0.09 fl
ATP	2000 uM	1	0.09 fl
AC1-CaM	0 uM	0	0.09 fl
AC1	0.074074 uM	0	0.09 fl
AC2*	0 uM	0	0.09 fl
AC2	0.074074 uM	0	0.09 fl
AMP	0 uM	0	0.09 fl
cAMP-PDE	0.55556 uM	0	0.09 fl
cAMP-PDE*	0 uM	0	0.09 fl

PDE1	2.5926 uM	0	0.09 fl
CaM.PDE1	0 uM	0	0.09 fl
cAMP_in_dend	0 uM	0	5 fl
I1 (in PSD)	8 uM	0	0.01 fl
I1* (in PSD)	0 uM	0	0.01 fl
PP1-I1* (in PSD)	0 uM	0	0.01 fl
PP1-I1 (in PSD)	0 uM	0	0.01 fl
GluR23_M	3.5 uM	0	0.01 fl
GluR23_I	0.092593 uM	0	0.09 fl
total_mem	3.4667 uM	0	0.01 fl
AMPAR_deg	0 uM	1	0.09 fl
A_B	0 uM	0	0.09 fl
A831*_B	0 uM	0	0.09 fl
A845*_B	0 uM	0	0.09 fl
A831*845*_B	0 uM	0	0.09 fl
A_B845*	0 uM	0	0.09 fl
AA831*_B845*	0 uM	0	0.09 fl
A845*_B845*	0 uM	0	0.09 fl
A831*845*_B845*	0 uM	0	0.09 fl
A845*_B831*845*	0 uM	0	0.09 fl
A835*845*_B835*845*	0 uM	0	0.09 fl
A_B831*845*	0 uM	0	0.09 fl
A831*_B831*845*	0 uM	0	0.09 fl
A_B831*	0 uM	0	0.09 fl
A845*_B831*	0 uM	0	0.01 fl
A831*_B831*	0 uM	0	0.09 fl
A831*845*_B831*	0 uM	0	0.09 fl
A_B	0 uM	0	0.01 fl
A831*_B	0 uM	0	0.01 fl
A_B831*	0 uM	0	0.01 fl
A831*_B831*	0 uM	0	0.01 fl
A845*_B	0 uM	0	0.01 fl
A831*845*_B	0 uM	0	0.01 fl
A831*845*_B831*	0 uM	0	0.01 fl
A_B845*	0 uM	0	0.01 fl
A845*_B831*	0 uM	0	0.01 fl
A845*_B845*	0 uM	0	0.01 fl
A831*_B845*	0 uM	0	0.01 fl
A_B831*845*	0 uM	0	0.01 fl
A831*_B831*845*	0 uM	0	0.01 fl
A831*845*_B845*	0 uM	0	0.01 fl
A845*_B831*845*	0 uM	0	0.01 fl
A835*845*_B835*845*	0 uM	0	0.01 fl

Model Parameters for nested bistability model: Model 5

Concentration units: uM (micromolar) for rate constants presented as Kf, Kb, Km
#/cell for rate constants presented as kf, kb, k1, k2, k3. This formulation of rates may depend on cellular volume.
A few reactions represent traffic between compartments of different volumes. Where such reactions involve concentration units, please use the #/cell rate terms as they a
unambiguous.
Time units: Seconds in all cases.
Total Volume of Synapse = 0.1 femtoliters (fl)
Volume of cytosolic portion = 0.09 fl
Volume of Postsynaptic Density (PSD) = 0.01 fl

The enzyme rates are related as follows:
Km = (k2 + k3)/k1 (after conversion of units)
Kcat = k3.
Ratio = k2/k3

Initial concentrations (CoInit) are mostly zero, except for a few key molecules.
There is a flag for 'buffered' in the molecule concentration table. When this flag is zero the molecule concentrations are computed
according to the reaction equations. If the flag is one the molecule concentration is held fixed to its initial concentration.

The entire model scheme is presented as composite tables for molecules, reactions and enzymes.

All equations.
Reactions

Reaction	kf	kb	Kf	Kb
CaMKII-PSD + CaM-Ca4-PSD <===> CaMKII-CaM-PSD	8.3333 #^-1.s^-1	0 s^-1	50 uM^-1.s^-1	0 s^-1
CaMKII-CaM + NMDAR <===> CaMKII-CaM-PSD	2e-05 #^-1.s^-1	0 s^-1	0.00108 uM^-1.s^-1	0 s^-1
CaMKII-thr286*-CaM + NMDAR <===> CaMKII-thr286-CaM-PSD	2e-05 #^-1.s^-1	0 s^-1	0.00108 uM^-1.s^-1	0 s^-1
CaMKII-PSD <===> CaMKII + NMDAR	0.3 s^-1	1e-05 #^-1.s^-1	0.3 s^-1	6e-05 uM^-1.s^-1
CaMKII-thr305-PSD <===> CaMK-thr305 + NMDAR	0.3 s^-1	1e-05 #^-1.s^-1	0.3 s^-1	6e-05 uM^-1.s^-1
CaMKII-thr286-PSD + CaM-Ca4-PSD <===> CaMKII-thr286-CaM-PSD	166.67 #^-1.s^-1	0.1 s^-1	1000 uM^-1.s^-1	0.1 s^-1
CaM-Ca4-PSD <===> CaM-Ca4	540 s^-1	60 s^-1	540 s^-1	60 s^-1
I1* + PP1-active_PSD <===> PP1-I1*	83.33 #^-1.s^-1	0.1 s^-1	499.98 uM^-1.s^-1	0.1 s^-1
CaM-Ca3 + Ca <===> CaM-Ca4	0.0086111 #^-1.s^-1	10 s^-1	0.465 uM^-1.s^-1	10 s^-1
GluR23_M <===> GluR23_I	0.00035 s^-1	0.0014 s^-1	0.00035 s^-1	0.0014 s^-1
CaMKII-CaM-PSD <===> CaM-Ca4-PSD + CaMKII-PSD	5 s^-1	0 #^-1.s^-1	5 s^-1	0 uM^-1.s^-1
PKC-control <===> PKC-active	2.5 s^-1	2.5 s^-1	2.5 s^-1	2.5 s^-1
Ca_control_cyt <===> Ca	100 s^-1	100 s^-1	100 s^-1	100 s^-1
Ca_control_PSD <===> Ca-PSD	100 s^-1	100 s^-1	100 s^-1	100 s^-1
basal_CaMKII_PSD_control <===> basal_CaMKII_PSD	1 s^-1	1 s^-1	1 s^-1	1 s^-1
AMPAR_bulk <===> A_B	0.018 s^-1	1 s^-1	0.018 s^-1	1 s^-1
CaM-Ca4 + CaMKII <===> CaMKII-CaM	0.92592 #^-1.s^-1	5 s^-1	50 uM^-1.s^-1	5 s^-1
CaMKII-thr286 + CaM-Ca4 <===> CaMKII-thr286*-CaM	18.522 #^-1.s^-1	0.1 s^-1	1000.2 uM^-1.s^-1	0.1 s^-1
CaM + 2 Ca <===> CaM-TR2-Ca2	0.024691 #^-2.s^-1	72 s^-1	71.999 uM^-2.s^-1	72 s^-1
CaM-TR2-Ca2 + Ca <===> CaM-Ca3	0.066667 #^-1.s^-1	10 s^-1	3.6 uM^-1.s^-1	10 s^-1
neurogranin + CaM <===> neurogranin-CaM	0.0055556 #^-1.s^-1	1 s^-1	0.3 uM^-1.s^-1	1 s^-1
neurogranin* <===> neurogranin	0.005 s^-1	0 s^-1	0.005 s^-1	0 s^-1
CaM-PSD + 2 Ca-PSD <===> CaM-TR2-Ca2-PSD	2 #^-2.s^-1	72 s^-1	72 uM^-2.s^-1	72 s^-1
CaM-TR2-Ca2-PSD + Ca-PSD <===> CaM-Ca3-PSD	0.6 #^-1.s^-1	10 s^-1	3.6 uM^-1.s^-1	10 s^-1
CaM-Ca3-PSD + Ca-PSD <===> CaM-Ca4-PSD	0.077502 #^-1.s^-1	10 s^-1	0.46501 uM^-1.s^-1	10 s^-1
neurogranin_PSD + CaM-PSD <===> neurogranin-CaM_PSD	0.05 #^-1.s^-1	1 s^-1	0.3 uM^-1.s^-1	1 s^-1
neurogranin*_PSD <===> neurogranin_PSD	0.005 s^-1	0 s^-1	0.005 s^-1	0 s^-1
I1* + PP1-active <===> PP1-I1*	9.2589 #^-1.s^-1	0.1 s^-1	499.98 uM^-1.s^-1	0.1 s^-1
PP1-I1 <===> PP1-active + I1	1 s^-1	0 #^-1.s^-1	1 s^-1	0 uM^-1.s^-1
2 Ca + CaNAB-Ca2 <===> CaNAB-Ca4	0.0012346 #^-2.s^-1	1 s^-1	3.6001 uM^-2.s^-1	1 s^-1
CaNAB + 2 Ca <===> CaNAB-Ca2	3.4321 #^-2.s^-1	1 s^-1	10008 uM^-2.s^-1	1 s^-1
CaM-Ca4 + CaNAB-Ca4 <===> CaM_Ca_n-CaNAB	11.111 #^-1.s^-1	1 s^-1	599.99 uM^-1.s^-1	1 s^-1
R2C2 + cAMP <===> R2C2-cAMP	1 #^-1.s^-1	33 s^-1	54 uM^-1.s^-1	33 s^-1
R2C2-cAMP + cAMP <===> R2C2-cAMP2	1 #^-1.s^-1	33 s^-1	54 uM^-1.s^-1	33 s^-1
R2C2-cAMP2 + cAMP <===> R2C2-cAMP3	1.3889 #^-1.s^-1	110 s^-1	75.001 uM^-1.s^-1	110 s^-1
cAMP + R2C2-cAMP3 <===> R2C2-cAMP4	1.3889 #^-1.s^-1	32.5 s^-1	75.001 uM^-1.s^-1	32.5 s^-1
R2C2-cAMP4 <===> PKA-active + R2C-cAMP4	60 s^-1	0.33333 #^-1.s^-1	60 s^-1	18 uM^-1.s^-1
R2C-cAMP4 <===> PKA-active + R2-cAMP4	60 s^-1	0.33333 #^-1.s^-1	60 s^-1	18 uM^-1.s^-1
PKA-active + PKA-inhibitor <===> inhibited-PKA	1.1111 #^-1.s^-1	1 s^-1	59.999 uM^-1.s^-1	1 s^-1
CaM-Ca4 + AC1 <===> AC1-CaM	0.92592 #^-1.s^-1	1 s^-1	50 uM^-1.s^-1	1 s^-1
AC2* <===> AC2	0.1 s^-1	0 s^-1	0.1 s^-1	0 s^-1
cAMP-PDE* <===> cAMP-PDE	0.01 s^-1	0 s^-1	0.01 s^-1	0 s^-1
PDE1 + CaM-Ca4 <===> CaM.PDE1	13.333 #^-1.s^-1	5 s^-1	719.98 uM^-1.s^-1	5 s^-1
cAMP <===> cAMP_in_dend	300 s^-1	5.4 s^-1	300 s^-1	5.4 s^-1
PP1-I1 <===> I1 + PP1-active_PSD	1 s^-1	0 #^-1.s^-1	1 s^-1	0 uM^-1.s^-1
A831*_B831* <===> A831*_B831* + Anchor	0.0008 s^-1	0 #^-1.s^-1	0.0008 s^-1	0 uM^-1.s^-1
A_B831* <===> A_B831* + Anchor	0.0008 s^-1	0 #^-1.s^-1	0.0008 s^-1	0 uM^-1.s^-1
A831*_B <===> A831*_B + Anchor	0.0008 s^-1	0 #^-1.s^-1	0.0008 s^-1	0 uM^-1.s^-1
A_B <===> A_B + Anchor	0.0008 s^-1	0 #^-1.s^-1	0.0008 s^-1	0 uM^-1.s^-1
A835*845*_B835*845* <===> AMPAR_deg	3.6e-05 s^-1	0 s^-1	3.6e-05 s^-1	0 s^-1
A845*_B831*845* <===> AMPAR_deg	3.6e-05 s^-1	0 s^-1	3.6e-05 s^-1	0 s^-1
A831*845*_B845* <===> AMPAR_deg	3.6e-05 s^-1	0 s^-1	3.6e-05 s^-1	0 s^-1
A845*_B845* <===> AMPAR_deg	3.6e-05 s^-1	0 s^-1	3.6e-05 s^-1	0 s^-1
A835*845*_B835*845* + Anchor <===> A835*845*_B835*845*	0.0002 #^-1.s^-1	0.008 s^-1	0.0108 uM^-1.s^-1	0.008 s^-1
A845*_B831*845* + Anchor <===> A845*_B831*845*	0.0002 #^-1.s^-1	0.008 s^-1	0.0108 uM^-1.s^-1	0.008 s^-1
A831*845*_B845* + Anchor <===> A831*845*_B845*	0.0002 #^-1.s^-1	0.008 s^-1	0.0108 uM^-1.s^-1	0.008 s^-1
A845*_B845* + Anchor <===> A845*_B845*	0.0002 #^-1.s^-1	0.008 s^-1	0.0108 uM^-1.s^-1	0.008 s^-1

Enzymes for group /##[]

Enzyme-reaction	k1	k2	k3	Km	kcat	ratio
CaMKII-thr286*-CaM ---PP1-active--> CaMKII-CaM	0.045397 #^-1.s^-1	10 s^-1	2.5 s^-1	5.099 uM	2.5 s^-1	4
CaMKII-thr286 ---PP1-active--> CaMKII	0.045397 #^-1.s^-1	10 s^-1	2.5 s^-1	5.099 uM	2.5 s^-1	4
CaMKII*** ---PP1-active--> CaMKII-thr286	0.045397 #^-1.s^-1	10 s^-1	2.5 s^-1	5.099 uM	2.5 s^-1	4
CaMKII*** ---PP1-active--> CaMK-thr305	0.045397 #^-1.s^-1	10 s^-1	2.5 s^-1	5.099 uM	2.5 s^-1	4
CaMK-thr305 ---PP1-active--> CaMKII	0.045397 #^-1.s^-1	10 s^-1	2.5 s^-1	5.099 uM	2.5 s^-1	4
cAMP-PDE ---PKA-active--> cAMP-PDE*	0.11111 #^-1.s^-1	36 s^-1	9 s^-1	7.5001 uM	9 s^-1	4
I1 ---PKA-active--> I1*	0.11111 #^-1.s^-1	36 s^-1	9 s^-1	7.5001 uM	9 s^-1	4
I1 ---PKA-active--> I1*	0.11111 #^-1.s^-1	36 s^-1	9 s^-1	7.5001 uM	9 s^-1	4
A_B ---PKA-active--> A845*_B	0.074072 #^-1.s^-1	24 s^-1	6 s^-1	7.5002 uM	6 s^-1	4
A831*_B ---PKA-active--> A831*845*_B	0.074072 #^-1.s^-1	24 s^-1	6 s^-1	7.5002 uM	6 s^-1	4
A_B831* ---PKA-active--> A845*_B831*	0.074072 #^-1.s^-1	24 s^-1	6 s^-1	7.5002 uM	6 s^-1	4
A831*_B831* ---PKA-active--> A831*845*_B831*	0.074072 #^-1.s^-1	24 s^-1	6 s^-1	7.5002 uM	6 s^-1	4
A_B ---PKA-active--> A_B845*	0.074072 #^-1.s^-1	24 s^-1	6 s^-1	7.5002 uM	6 s^-1	4
A831*_B ---PKA-active--> AA831*_B845*	0.074072 #^-1.s^-1	24 s^-1	6 s^-1	7.5002 uM	6 s^-1	4
A_B831* ---PKA-active--> A_B831*845*	0.074072 #^-1.s^-1	24 s^-1	6 s^-1	7.5002 uM	6 s^-1	4
A831*_B831* ---PKA-active--> A831*_B831*845*	0.074072 #^-1.s^-1	24 s^-1	6 s^-1	7.5002 uM	6 s^-1	4
A_B845* ---PKA-active--> A845*_B845*	0.074072 #^-1.s^-1	24 s^-1	6 s^-1	7.5002 uM	6 s^-1	4
AA831*_B845* ---PKA-active--> A831*845*_B845*	0.074072 #^-1.s^-1	24 s^-1	6 s^-1	7.5002 uM	6 s^-1	4
A_B831*845* ---PKA-active--> A845*_B831*845*	0.074072 #^-1.s^-1	24 s^-1	6 s^-1	7.5002 uM	6 s^-1	4
A831*_B831*845* ---PKA-active--> A835*845*_B835*845*	0.074072 #^-1.s^-1	24 s^-1	6 s^-1	7.5002 uM	6 s^-1	4
A845*_B ---PKA-active--> A845*_B845*	0.074072 #^-1.s^-1	24 s^-1	6 s^-1	7.5002 uM	6 s^-1	4
A831*845*_B ---PKA-active--> A831*845*_B845*	0.074072 #^-1.s^-1	24 s^-1	6 s^-1	7.5002 uM	6 s^-1	4
A845*_B831* ---PKA-active--> A845*_B831*845*	0.074072 #^-1.s^-1	24 s^-1	6 s^-1	7.5002 uM	6 s^-1	4
A831*845*_B831* ---PKA-active--> A835*845*_B835*845*	0.074072 #^-1.s^-1	24 s^-1	6 s^-1	7.5002 uM	6 s^-1	4
A_B845* ---PKA-active--> A845*_B845*	0.074072 #^-1.s^-1	24 s^-1	6 s^-1	7.5002 uM	6 s^-1	4
A845*_B ---PKA-active--> A845*_B845*	0.074072 #^-1.s^-1	24 s^-1	6 s^-1	7.5002 uM	6 s^-1	4
A_B ---PKA-active--> A845*_B	0.074072 #^-1.s^-1	24 s^-1	6 s^-1	7.5002 uM	6 s^-1	4
A_B ---PKA-active--> A_B845*	0.074072 #^-1.s^-1	24 s^-1	6 s^-1	7.5002 uM	6 s^-1	4
A831*_B845* ---PKA-active--> A831*845*_B845*	0.074072 #^-1.s^-1	24 s^-1	6 s^-1	7.5002 uM	6 s^-1	4
A831*845*_B ---PKA-active--> A831*845*_B845*	0.074072 #^-1.s^-1	24 s^-1	6 s^-1	7.5002 uM	6 s^-1	4
A831*_B ---PKA-active--> A831*_B845*	0.074072 #^-1.s^-1	24 s^-1	6 s^-1	7.5002 uM	6 s^-1	4
A831*_B ---PKA-active--> A831*845*_B	0.074072 #^-1.s^-1	24 s^-1	6 s^-1	7.5002 uM	6 s^-1	4
A_B831*845* ---PKA-active--> A845*_B831*845*	0.074072 #^-1.s^-1	24 s^-1	6 s^-1	7.5002 uM	6 s^-1	4
A845*_B831* ---PKA-active--> A845*_B831*845*	0.074072 #^-1.s^-1	24 s^-1	6 s^-1	7.5002 uM	6 s^-1	4
A_B831* ---PKA-active--> A_B831*845*	0.074072 #^-1.s^-1	24 s^-1	6 s^-1	7.5002 uM	6 s^-1	4
A_B831* ---PKA-active--> A845*_B831*	0.074072 #^-1.s^-1	24 s^-1	6 s^-1	7.5002 uM	6 s^-1	4
A831*_B831*845* ---PKA-active--> A835*845*_B835*845*	0.074072 #^-1.s^-1	24 s^-1	6 s^-1	7.5002 uM	6 s^-1	4
A831*845*_B831* ---PKA-active--> A835*845*_B835*845*	0.074072 #^-1.s^-1	24 s^-1	6 s^-1	7.5002 uM	6 s^-1	4
A831*_B831* ---PKA-active--> A831*_B831*845*	0.074072 #^-1.s^-1	24 s^-1	6 s^-1	7.5002 uM	6 s^-1	4
A831*_B831* ---PKA-active--> A831*845*_B831*	0.074072 #^-1.s^-1	24 s^-1	6 s^-1	7.5002 uM	6 s^-1	4
I1* ---PP2A--> I1	0.01196 #^-1.s^-1	8.3334 s^-1	2 s^-1	16 uM	2 s^-1	4.1667
PP1-I1* ---PP2A--> PP1-I1	0.01196 #^-1.s^-1	8.3334 s^-1	2 s^-1	16 uM	2 s^-1	4.1667
I1* ---PP2A--> I1	0.01196 #^-1.s^-1	8.3334 s^-1	2 s^-1	16 uM	2 s^-1	4.1667
PP1-I1* ---PP2A--> PP1-I1	0.01196 #^-1.s^-1	8.3334 s^-1	2 s^-1	16 uM	2 s^-1	4.1667
I1* ---CaNAB-Ca4--> I1	0.00063333 #^-1.s^-1	0.136 s^-1	0.034 s^-1	4.9708 uM	0.034 s^-1	4
I1* ---CaNAB-Ca4--> I1	0.00063334 #^-1.s^-1	0.136 s^-1	0.034 s^-1	4.9707 uM	0.034 s^-1	4
CaMKII-thr286-PSD ---tot-auto-PSD--> CaMKII***-PSD	0.01 #^-1.s^-1	24 s^-1	6 s^-1	500 uM	6 s^-1	4
CaMKII-CaM-PSD ---tot-auto-PSD--> CaMKII-thr286-CaM-PSD	0.00083333 #^-1.s^-1	2 s^-1	0.5 s^-1	500 uM	0.5 s^-1	4
CaMKII-PSD ---tot-auto-PSD--> CaMKII-thr286-PSD	0.0033333 #^-1.s^-1	8 s^-1	2 s^-1	500.01 uM	2 s^-1	4
A845*_B845* ---actCaMKII-PSD--> A831*845*_B845*	0.0046296 #^-1.s^-1	2 s^-1	0.5 s^-1	90.001 uM	0.5 s^-1	4
A_B845* ---actCaMKII-PSD--> A831*_B845*	0.0046296 #^-1.s^-1	2 s^-1	0.5 s^-1	90.001 uM	0.5 s^-1	4
A845*_B ---actCaMKII-PSD--> A831*845*_B	0.0046296 #^-1.s^-1	2 s^-1	0.5 s^-1	90.001 uM	0.5 s^-1	4
A_B ---actCaMKII-PSD--> A831*_B	0.0046296 #^-1.s^-1	2 s^-1	0.5 s^-1	90.001 uM	0.5 s^-1	4
A845*_B845* ---actCaMKII-PSD--> A845*_B831*845*	0.0046296 #^-1.s^-1	2 s^-1	0.5 s^-1	90.001 uM	0.5 s^-1	4
A_B845* ---actCaMKII-PSD--> A_B831*845*	0.0046296 #^-1.s^-1	2 s^-1	0.5 s^-1	90.001 uM	0.5 s^-1	4
A845*_B ---actCaMKII-PSD--> A845*_B831*	0.0046296 #^-1.s^-1	2 s^-1	0.5 s^-1	90.001 uM	0.5 s^-1	4
A_B ---actCaMKII-PSD--> A_B831*	0.0046296 #^-1.s^-1	2 s^-1	0.5 s^-1	90.001 uM	0.5 s^-1	4
A845*_B831*845* ---actCaMKII-PSD--> A835*845*_B835*845*	0.0046296 #^-1.s^-1	2 s^-1	0.5 s^-1	90.001 uM	0.5 s^-1	4
A_B831*845* ---actCaMKII-PSD--> A831*_B831*845*	0.0046296 #^-1.s^-1	2 s^-1	0.5 s^-1	90.001 uM	0.5 s^-1	4
A845*_B831* ---actCaMKII-PSD--> A831*845*_B831*	0.0046296 #^-1.s^-1	2 s^-1	0.5 s^-1	90.001 uM	0.5 s^-1	4
A_B831* ---actCaMKII-PSD--> A831*_B831*	0.0046296 #^-1.s^-1	2 s^-1	0.5 s^-1	90.001 uM	0.5 s^-1	4
A831*845*_B845* ---actCaMKII-PSD--> A835*845*_B835*845*	0.0046296 #^-1.s^-1	2 s^-1	0.5 s^-1	90.001 uM	0.5 s^-1	4
A831*_B845* ---actCaMKII-PSD--> A831*_B831*845*	0.0046296 #^-1.s^-1	2 s^-1	0.5 s^-1	90.001 uM	0.5 s^-1	4
A831*845*_B ---actCaMKII-PSD--> A831*845*_B831*	0.0046296 #^-1.s^-1	2 s^-1	0.5 s^-1	90.001 uM	0.5 s^-1	4
A831*_B ---actCaMKII-PSD--> A831*_B831*	0.0046296 #^-1.s^-1	2 s^-1	0.5 s^-1	90.001 uM	0.5 s^-1	4
A845*_B845* ---PP1-active_PSD--> A_B845*	0.14583 #^-1.s^-1	0.68 s^-1	0.17 s^-1	0.97145 uM	0.17 s^-1	4
A845*_B845* ---PP1-active_PSD--> A845*_B	0.14583 #^-1.s^-1	0.68 s^-1	0.17 s^-1	0.97145 uM	0.17 s^-1	4
A845*_B ---PP1-active_PSD--> A_B	0.14583 #^-1.s^-1	0.68 s^-1	0.17 s^-1	0.97145 uM	0.17 s^-1	4
A_B845* ---PP1-active_PSD--> A_B	0.14583 #^-1.s^-1	0.68 s^-1	0.17 s^-1	0.97145 uM	0.17 s^-1	4
A831*845*_B845* ---PP1-active_PSD--> A831*_B845*	0.14583 #^-1.s^-1	0.68 s^-1	0.17 s^-1	0.97145 uM	0.17 s^-1	4
A831*845*_B845* ---PP1-active_PSD--> A831*845*_B	0.14583 #^-1.s^-1	0.68 s^-1	0.17 s^-1	0.97145 uM	0.17 s^-1	4
A831*_B845* ---PP1-active_PSD--> A831*_B	0.14583 #^-1.s^-1	0.68 s^-1	0.17 s^-1	0.97145 uM	0.17 s^-1	4
A831*845*_B ---PP1-active_PSD--> A831*_B	0.14583 #^-1.s^-1	0.68 s^-1	0.17 s^-1	0.97145 uM	0.17 s^-1	4
A831*845*_B845* ---PP1-active_PSD--> A845*_B845*	0.14583 #^-1.s^-1	1.4 s^-1	0.35 s^-1	2 uM	0.35 s^-1	4
A831*_B845* ---PP1-active_PSD--> A_B845*	0.14583 #^-1.s^-1	1.4 s^-1	0.35 s^-1	2 uM	0.35 s^-1	4
A831*845*_B ---PP1-active_PSD--> A845*_B	0.14583 #^-1.s^-1	1.4 s^-1	0.35 s^-1	2 uM	0.35 s^-1	4
A831*_B ---PP1-active_PSD--> A_B	0.14583 #^-1.s^-1	1.4 s^-1	0.35 s^-1	2 uM	0.35 s^-1	4
A845*_B831*845* ---PP1-active_PSD--> A_B831*845*	0.14583 #^-1.s^-1	0.68 s^-1	0.17 s^-1	0.97145 uM	0.17 s^-1	4
A845*_B831*845* ---PP1-active_PSD--> A845*_B831*	0.14583 #^-1.s^-1	0.68 s^-1	0.17 s^-1	0.97145 uM	0.17 s^-1	4
A_B831*845* ---PP1-active_PSD--> A_B831*	0.14583 #^-1.s^-1	0.68 s^-1	0.17 s^-1	0.97145 uM	0.17 s^-1	4
A845*_B831* ---PP1-active_PSD--> A_B831*	0.14583 #^-1.s^-1	0.68 s^-1	0.17 s^-1	0.97145 uM	0.17 s^-1	4
A835*845*_B835*845* ---PP1-active_PSD--> A831*_B831*845*	0.14583 #^-1.s^-1	0.68 s^-1	0.17 s^-1	0.97145 uM	0.17 s^-1	4
A835*845*_B835*845* ---PP1-active_PSD--> A831*845*_B831*	0.14583 #^-1.s^-1	0.68 s^-1	0.17 s^-1	0.97145 uM	0.17 s^-1	4
A831*_B831*845* ---PP1-active_PSD--> A831*_B831*	0.14583 #^-1.s^-1	0.68 s^-1	0.17 s^-1	0.97145 uM	0.17 s^-1	4
A831*845*_B831* ---PP1-active_PSD--> A831*_B831*	0.14583 #^-1.s^-1	0.68 s^-1	0.17 s^-1	0.97145 uM	0.17 s^-1	4
A_B831*845* ---PP1-active_PSD--> A_B845*	0.14583 #^-1.s^-1	1.4 s^-1	0.35 s^-1	2 uM	0.35 s^-1	4
A845*_B831*845* ---PP1-active_PSD--> A845*_B845*	0.14583 #^-1.s^-1	1.4 s^-1	0.35 s^-1	2 uM	0.35 s^-1	4
A845*_B831* ---PP1-active_PSD--> A845*_B	0.14583 #^-1.s^-1	1.4 s^-1	0.35 s^-1	2 uM	0.35 s^-1	4
A_B831* ---PP1-active_PSD--> A_B	0.14583 #^-1.s^-1	1.4 s^-1	0.35 s^-1	2 uM	0.35 s^-1	4
A835*845*_B835*845* ---PP1-active_PSD--> A845*_B831*845*	0.14583 #^-1.s^-1	1.4 s^-1	0.35 s^-1	2 uM	0.35 s^-1	4

A831*_B831*845* ---PP1-active_PSD--> A_B831*845*	0.14583 #^-1.s^-1	1.4 s^-1	0.35 s^-1	2 uM	0.35 s^-1	4
A831*845*_B831* ---PP1-active_PSD--> A845*_B831*	0.14583 #^-1.s^-1	1.4 s^-1	0.35 s^-1	2 uM	0.35 s^-1	4
A831*_B831* ---PP1-active_PSD--> A_B831*	0.14583 #^-1.s^-1	1.4 s^-1	0.35 s^-1	2 uM	0.35 s^-1	4
A835*845*_B835*845* ---PP1-active_PSD--> A831*845*_B845*	0.14583 #^-1.s^-1	1.4 s^-1	0.35 s^-1	2 uM	0.35 s^-1	4
A831*_B831*845* ---PP1-active_PSD--> A831*_B845*	0.14583 #^-1.s^-1	1.4 s^-1	0.35 s^-1	2 uM	0.35 s^-1	4
A831*845*_B831* ---PP1-active_PSD--> A831*845*_B	0.14583 #^-1.s^-1	1.4 s^-1	0.35 s^-1	2 uM	0.35 s^-1	4
A831*_B831* ---PP1-active_PSD--> A831*_B	0.14583 #^-1.s^-1	1.4 s^-1	0.35 s^-1	2 uM	0.35 s^-1	4
neurogranin ---PKC-active--> neurogranin*	0.0018889 #^-1.s^-1	2.34 s^-1	0.58 s^-1	28.627 uM	0.58 s^-1	4.0345
neurogranin-CaM ---PKC-active--> CaM + neurogranin*	0.0011333 #^-1.s^-1	1.4 s^-1	0.35 s^-1	28.596 uM	0.35 s^-1	4
AC2 ---PKC-active--> AC2*	0.011111 #^-1.s^-1	16 s^-1	4 s^-1	33.334 uM	4 s^-1	4
neurogranin_PSD ---PKC-active--> neurogranin*_PSD	0.0018889 #^-1.s^-1	2.34 s^-1	0.58 s^-1	28.627 uM	0.58 s^-1	4.0345
neurogranin-CaM_PSD ---PKC-active--> CaM-PSD + neurogranin*_PSD	0.0011333 #^-1.s^-1	1.4 s^-1	0.35 s^-1	28.596 uM	0.35 s^-1	4
neurogranin* ---CaM_Ca_n-CaNAB--> neurogranin	0.0061778 #^-1.s^-1	2.67 s^-1	0.67 s^-1	10.012 uM	0.67 s^-1	3.9851
I1* ---CaM_Ca_n-CaNAB--> I1	0.0063333 #^-1.s^-1	1.36 s^-1	0.34 s^-1	4.9708 uM	0.34 s^-1	4
PP1-I1* ---CaM_Ca_n-CaNAB--> PP1-I1	0.0063333 #^-1.s^-1	1.36 s^-1	0.34 s^-1	4.9708 uM	0.34 s^-1	4
I1* ---CaM_Ca_n-CaNAB--> I1	0.0063334 #^-1.s^-1	1.36 s^-1	0.34 s^-1	4.9707 uM	0.34 s^-1	4
PP1-I1* ---CaM_Ca_n-CaNAB--> PP1-I1	0.0063334 #^-1.s^-1	1.36 s^-1	0.34 s^-1	4.9707 uM	0.34 s^-1	4
A845*_B ---CaM_Ca_n-CaNAB--> A_B	0.037256 #^-1.s^-1	8 s^-1	2 s^-1	4.9706 uM	2 s^-1	4
A831*845*_B ---CaM_Ca_n-CaNAB--> A831*_B	0.037256 #^-1.s^-1	8 s^-1	2 s^-1	4.9706 uM	2 s^-1	4
A845*_B831* ---CaM_Ca_n-CaNAB--> A_B831*	0.037256 #^-1.s^-1	8 s^-1	2 s^-1	4.9706 uM	2 s^-1	4
A831*845*_B831* ---CaM_Ca_n-CaNAB--> A831*_B831*	0.037256 #^-1.s^-1	8 s^-1	2 s^-1	4.9706 uM	2 s^-1	4
A_B845* ---CaM_Ca_n-CaNAB--> A_B	0.037256 #^-1.s^-1	8 s^-1	2 s^-1	4.9706 uM	2 s^-1	4
AA831*_B845* ---CaM_Ca_n-CaNAB--> A831*_B	0.037256 #^-1.s^-1	8 s^-1	2 s^-1	4.9706 uM	2 s^-1	4
A_B831*845* ---CaM_Ca_n-CaNAB--> A_B831*	0.037256 #^-1.s^-1	8 s^-1	2 s^-1	4.9706 uM	2 s^-1	4
A831*_B831*845* ---CaM_Ca_n-CaNAB--> A831*_B831*	0.037256 #^-1.s^-1	8 s^-1	2 s^-1	4.9706 uM	2 s^-1	4
A845*_B845* ---CaM_Ca_n-CaNAB--> A_B845*	0.037256 #^-1.s^-1	8 s^-1	2 s^-1	4.9706 uM	2 s^-1	4
A831*845*_B845* ---CaM_Ca_n-CaNAB--> AA831*_B845*	0.037256 #^-1.s^-1	8 s^-1	2 s^-1	4.9706 uM	2 s^-1	4
A845*_B831*845* ---CaM_Ca_n-CaNAB--> A_B831*845*	0.037256 #^-1.s^-1	8 s^-1	2 s^-1	4.9706 uM	2 s^-1	4
A835*845*_B835*845* ---CaM_Ca_n-CaNAB--> A831*_B831*845*	0.037256 #^-1.s^-1	8 s^-1	2 s^-1	4.9706 uM	2 s^-1	4
A845*_B845* ---CaM_Ca_n-CaNAB--> A845*_B	0.037256 #^-1.s^-1	8 s^-1	2 s^-1	4.9706 uM	2 s^-1	4
A831*845*_B845* ---CaM_Ca_n-CaNAB--> A831*845*_B	0.037256 #^-1.s^-1	8 s^-1	2 s^-1	4.9706 uM	2 s^-1	4
A845*_B831*845* ---CaM_Ca_n-CaNAB--> A845*_B831*	0.037256 #^-1.s^-1	8 s^-1	2 s^-1	4.9706 uM	2 s^-1	4
A835*845*_B835*845* ---CaM_Ca_n-CaNAB--> A831*845*_B831*	0.037256 #^-1.s^-1	8 s^-1	2 s^-1	4.9706 uM	2 s^-1	4
A845*_B845* ---CaM_Ca_n-CaNAB--> A_B845*	0.037256 #^-1.s^-1	8 s^-1	2 s^-1	4.9706 uM	2 s^-1	4
A845*_B845* ---CaM_Ca_n-CaNAB--> A845*_B	0.037256 #^-1.s^-1	8 s^-1	2 s^-1	4.9706 uM	2 s^-1	4
A_B845* ---CaM_Ca_n-CaNAB--> A_B	0.037256 #^-1.s^-1	8 s^-1	2 s^-1	4.9706 uM	2 s^-1	4
A845*_B ---CaM_Ca_n-CaNAB--> A_B	0.037256 #^-1.s^-1	8 s^-1	2 s^-1	4.9706 uM	2 s^-1	4
A831*845*_B845* ---CaM_Ca_n-CaNAB--> A831*_B845*	0.037256 #^-1.s^-1	8 s^-1	2 s^-1	4.9706 uM	2 s^-1	4
A831*845*_B845* ---CaM_Ca_n-CaNAB--> A831*845*_B	0.037256 #^-1.s^-1	8 s^-1	2 s^-1	4.9706 uM	2 s^-1	4
A831*_B845* ---CaM_Ca_n-CaNAB--> A831*_B	0.037256 #^-1.s^-1	8 s^-1	2 s^-1	4.9706 uM	2 s^-1	4
A831*845*_B ---CaM_Ca_n-CaNAB--> A831*_B	0.037256 #^-1.s^-1	8 s^-1	2 s^-1	4.9706 uM	2 s^-1	4
A845*_B831*845* ---CaM_Ca_n-CaNAB--> A_B831*845*	0.037256 #^-1.s^-1	8 s^-1	2 s^-1	4.9706 uM	2 s^-1	4
A845*_B831*845* ---CaM_Ca_n-CaNAB--> A845*_B831*	0.037256 #^-1.s^-1	8 s^-1	2 s^-1	4.9706 uM	2 s^-1	4
A_B831*845* ---CaM_Ca_n-CaNAB--> A_B831*	0.037256 #^-1.s^-1	8 s^-1	2 s^-1	4.9706 uM	2 s^-1	4
A845*_B831* ---CaM_Ca_n-CaNAB--> A_B831*	0.037256 #^-1.s^-1	8 s^-1	2 s^-1	4.9706 uM	2 s^-1	4
A835*845*_B835*845* ---CaM_Ca_n-CaNAB--> A831*_B831*845*	0.037256 #^-1.s^-1	8 s^-1	2 s^-1	4.9706 uM	2 s^-1	4
A835*845*_B835*845* ---CaM_Ca_n-CaNAB--> A831*845*_B831*	0.037256 #^-1.s^-1	8 s^-1	2 s^-1	4.9706 uM	2 s^-1	4
A831*845*_B831* ---CaM_Ca_n-CaNAB--> A831*_B831*	0.037256 #^-1.s^-1	8 s^-1	2 s^-1	4.9706 uM	2 s^-1	4
A831*_B831*845* ---CaM_Ca_n-CaNAB--> A831*_B831*	0.037256 #^-1.s^-1	8 s^-1	2 s^-1	4.9706 uM	2 s^-1	4
neurogranin*_PSD ---CaM_Ca_n-CaNAB--> neurogranin_PSD	0.0061778 #^-1.s^-1	2.67 s^-1	0.67 s^-1	10.012 uM	0.67 s^-1	3.9851
CaMKII-thr286-PSD ---tot-CaM-CaMKII-PSD--> CaMKII***-PSD	0.015625 #^-1.s^-1	24 s^-1	6 s^-1	320 uM	6 s^-1	4
CaMKII-CaM-PSD ---tot-CaM-CaMKII-PSD--> CaMKII-thr286-CaM-PSD	0.0013021 #^-1.s^-1	2 s^-1	0.5 s^-1	320 uM	0.5 s^-1	4
CaMKII-PSD ---tot-CaM-CaMKII-PSD--> CaMKII-thr286-PSD	0.0052083 #^-1.s^-1	8 s^-1	2 s^-1	320 uM	2 s^-1	4
CaMKII-thr286-CaM-PSD ---PP1-active_CaMKII_PSD--> CaMKII-CaM-PSD	0.083333 #^-1.s^-1	0.8 s^-1	0.2 s^-1	2 uM	0.2 s^-1	4
CaMKII-thr286-PSD ---PP1-active_CaMKII_PSD--> CaMKII-PSD	0.083333 #^-1.s^-1	0.8 s^-1	0.2 s^-1	2 uM	0.2 s^-1	4
CaMKII***-PSD ---PP1-active_CaMKII_PSD--> CaMKII-thr286-PSD	0.083333 #^-1.s^-1	0.8 s^-1	0.2 s^-1	2 uM	0.2 s^-1	4
CaMKII***-PSD ---PP1-active_CaMKII_PSD--> CaMKII-thr305-PSD	0.083333 #^-1.s^-1	0.8 s^-1	0.2 s^-1	2 uM	0.2 s^-1	4
CaMKII-thr305-PSD ---PP1-active_CaMKII_PSD--> CaMKII-PSD	0.083333 #^-1.s^-1	0.8 s^-1	0.2 s^-1	2 uM	0.2 s^-1	4
CaMKII-thr286 ---tot_CaM_CaMKII--> CaMKII***	0.0024474 #^-1.s^-1	24 s^-1	6 s^-1	227 uM	6 s^-1	4
CaMKII-CaM ---tot_CaM_CaMKII--> CaMKII-thr286*-CaM	0.00020395 #^-1.s^-1	2 s^-1	0.5 s^-1	227 uM	0.5 s^-1	4
CaMKII-thr286 ---tot_autonomous_CaMKII--> CaMKII***	0.0015873 #^-1.s^-1	24 s^-1	6 s^-1	350 uM	6 s^-1	4
CaMKII-CaM ---tot_autonomous_CaMKII--> CaMKII-thr286*-CaM	0.00013228 #^-1.s^-1	2 s^-1	0.5 s^-1	349.99 uM	0.5 s^-1	4
ATP ---AC1-CaM--> cAMP	0.0013889 #^-1.s^-1	18 s^-1	4.5 s^-1	300 uM	4.5 s^-1	4
ATP ---AC2*--> cAMP	0.00061728 #^-1.s^-1	8 s^-1	2 s^-1	300 uM	2 s^-1	4
cAMP ---cAMP-PDE--> AMP	0.046667 #^-1.s^-1	40 s^-1	10 s^-1	19.841 uM	10 s^-1	4
cAMP ---cAMP-PDE*--> AMP	0.093333 #^-1.s^-1	80 s^-1	20 s^-1	19.841 uM	20 s^-1	4
cAMP ---PDE1--> AMP	0.0038889 #^-1.s^-1	6.67 s^-1	1.667 s^-1	39.7 uM	1.667 s^-1	4.0012
cAMP ---CaM.PDE1--> AMP	0.023333 #^-1.s^-1	40 s^-1	10 s^-1	39.683 uM	10 s^-1	4

Pools for group /##[]

name	InitialConc	buffered	Volume
CaM-Ca4	0 uM	0	0.09 fl
PP1-active	1.8 uM	0	0.09 fl
cAMP	0 uM	0	0.09 fl
Ca	0.08 uM	0	0.09 fl
PKA-active	0 uM	0	0.09 fl
CaM-Ca3	0 uM	0	0.09 fl
CaM-TR2-Ca2	0 uM	0	0.09 fl
PP2A	0.11111 uM	0	0.09 fl
CaNAB-Ca4	0 uM	0	0.09 fl
CaMKII-thr286-CaM-PSD	0 uM	0	0.01 fl
CaMKII-CaM-PSD	0 uM	0	0.01 fl
CaMKII-thr286-PSD	0 uM	0	0.01 fl
CaMKII-PSD	0 uM	0	0.01 fl
CaMKII***-PSD	0 uM	0	0.01 fl
tot-auto-PSD	2 uM	0	0.01 fl
CaM-TR2-Ca2-PSD	0 uM	0	0.01 fl
CaM-Ca3-PSD	0 uM	0	0.01 fl
CaM-Ca4-PSD	0 uM	0	0.01 fl
Ca-PSD	0.08 uM	0	0.01 fl
286P-PSD	0 uM	0	0.01 fl
actCaMKII-PSD	2 uM	0	0.01 fl
tot_CaMKII_PSD	2 uM	0	0.01 fl
tot_CaMKII_cyt	22 uM	0	0.09 fl
PP1-active_PSD	4 uM	0	0.01 fl
PKC-active	0.1 uM	0	0.09 fl
temp-PIP2	2.5 uM	1	0.09 fl
I_845-P	0 uM	0	0.09 fl
tot_I_GluR12	0 uM	0	0.09 fl
total_Int	0.096296 uM	0	0.09 fl
Ser845	0 uM	0	0.01 fl
Ser845-P	0 uM	0	0.01 fl
Ser845-PP	0 uM	0	0.01 fl
Ser831	0 uM	0	0.01 fl
Ser831-P	0 uM	0	0.01 fl
Ser831-PP	0 uM	0	0.01 fl
tot_mem_GluR12	0 uM	0	0.01 fl
act_CaMKII_cyt	2 uM	0	0.09 fl
NMDAR	120 uM	0	0.01 fl
CaM_Ca_n-CaNAB	0 uM	0	0.09 fl
basal_CaMKII_cyt	2 uM	1	0.09 fl
basal_CaMKII_PSD	2 uM	0	0.01 fl
PKC-control	0.1 uM	1	0.09 fl
Ca_control_cyt	0.08 uM	1	0.09 fl
Ca_control_PSD	0.08 uM	1	0.01 fl
basal_CaMKII_PSD_control	2 uM	1	0.01 fl
Anchor	27.333 uM	0	0.01 fl
I_845	0 uM	0	0.09 fl
I_845_PP	0 uM	0	0.09 fl
tot-CaM-CaMKII-PSD	0 uM	0	0.01 fl
CaMKII-thr305-PSD	0 uM	0	0.01 fl
AMPAR_bulk	0.0092593 uM	1	5 fl
PP1-active_CaMKII_PSD	2 uM	0	0.01 fl
CaMKII	20 uM	0	0.09 fl
CaMKII-CaM	0 uM	0	0.09 fl
CaMKII-thr286*-CaM	0 uM	0	0.09 fl
CaMKII***	0 uM	0	0.09 fl
CaMKII-thr286	0 uM	0	0.09 fl
tot_CaM_CaMKII	0 uM	0	0.09 fl
tot_autonomous_CaMKII	2 uM	0	0.09 fl
CaMK-thr305	0 uM	0	0.09 fl
CaM	26.333 uM	0	0.09 fl
neurogranin-CaM	0 uM	0	0.09 fl
neurogranin*	0 uM	0	0.09 fl
neurogranin	10 uM	0	0.09 fl
CaM-PSD	26.333 uM	0	0.01 fl
neurogranin-CaM_PSD	0 uM	0	0.01 fl
neurogranin_PSD	10 uM	0	0.01 fl
neurogranin*_PSD	0 uM	0	0.01 fl
I1	1.8 uM	0	0.09 fl
I1*	0 uM	0	0.09 fl
PP1-I1*	0 uM	0	0.09 fl
PP1-I1	0 uM	0	0.09 fl
CaNAB	1 uM	0	0.09 fl
CaNAB-Ca2	0 uM	0	0.09 fl
R2C2	0.5 uM	0	0.09 fl
R2C2-cAMP	0 uM	0	0.09 fl
R2C2-cAMP2	0 uM	0	0.09 fl
R2C2-cAMP3	0 uM	0	0.09 fl
R2C2-cAMP4	0 uM	0	0.09 fl
R2C-cAMP4	0 uM	0	0.09 fl
R2-cAMP4	0 uM	0	0.09 fl
PKA-inhibitor	0.25926 uM	0	0.09 fl
inhibited-PKA	0 uM	0	0.09 fl
ATP	2000 uM	1	0.09 fl
AC1-CaM	0 uM	0	0.09 fl
AC1	0.074074 uM	0	0.09 fl
AC2*	0 uM	0	0.09 fl
AC2	0.074074 uM	0	0.09 fl
AMP	0 uM	0	0.09 fl
cAMP-PDE	0.55556 uM	0	0.09 fl

cAMP-PDE*	0 uM	0	0.09 fl
PDE1	2.5926 uM	0	0.09 fl
CaM.PDE1	0 uM	0	0.09 fl
cAMP_in_dend	0 uM	0	5 fl
I1	4 uM	0	0.01 fl
I1*	0 uM	0	0.01 fl
PP1-I1*	0 uM	0	0.01 fl
PP1-I1	0 uM	0	0.01 fl
GluR23_M	3.5 uM	0	0.01 fl
GluR23_I	0.092593 uM	0	0.09 fl
total_mem	3.4667 uM	0	0.01 fl
AMPAR_deg	0 uM	1	0.09 fl
A_B	0 uM	0	0.09 fl
A831*_B	0 uM	0	0.09 fl
A845*_B	0 uM	0	0.09 fl
A831*845*_B	0 uM	0	0.09 fl
A_B845*	0 uM	0	0.09 fl
AA831*_B845*	0 uM	0	0.09 fl
A845*_B845*	0 uM	0	0.09 fl
A831*845*_B845*	0 uM	0	0.09 fl
A845*_B831*845*	0 uM	0	0.09 fl
A835*845*_B835*845*	0 uM	0	0.09 fl
A_B831*845*	0 uM	0	0.09 fl
A831*_B831*845*	0 uM	0	0.09 fl
A_B831*	0 uM	0	0.09 fl
A845*_B831*	0 uM	0	0.01 fl
A831*_B831*	0 uM	0	0.09 fl
A831*845*_B831*	0 uM	0	0.09 fl
A_B	0 uM	0	0.01 fl
A831*_B	0 uM	0	0.01 fl
A_B831*	0 uM	0	0.01 fl
A831*_B831*	0 uM	0	0.01 fl
A845*_B	0 uM	0	0.01 fl
A831*845*_B	0 uM	0	0.01 fl
A831*845*_B831*	0 uM	0	0.01 fl
A_B845*	0 uM	0	0.01 fl
A845*_B831*	0 uM	0	0.01 fl
A845*_B845*	0 uM	0	0.01 fl
A831*_B845*	0 uM	0	0.01 fl
A_B831*845*	0 uM	0	0.01 fl
A831*_B831*845*	0 uM	0	0.01 fl
A831*845*_B845*	0 uM	0	0.01 fl
A845*_B831*845*	0 uM	0	0.01 fl
A835*845*_B835*845*	0 uM	0	0.01 fl
